# Formal Enone α-Arylation via I(III)-Mediated Aryl Migration/Elimination

**DOI:** 10.1021/acs.orglett.1c00251

**Published:** 2021-02-26

**Authors:** Bruna S. Martins, Daniel Kaiser, Adriano Bauer, Irmgard Tiefenbrunner, Nuno Maulide

**Affiliations:** University of Vienna, Institute of Organic Chemistry, Währinger Strasse 38, 1090 Vienna, Austria

## Abstract

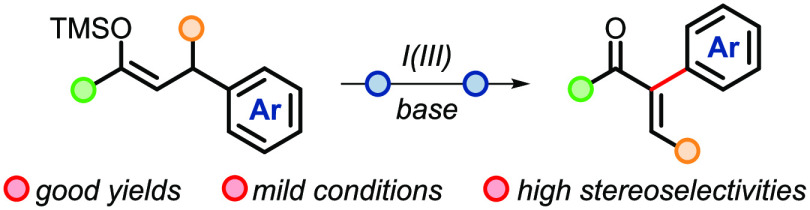

A formal enone α-arylation is described. This metal-free transformation relies on the I(III)-mediated skeletal reorganization of silyl enol ethers and features mild conditions, good yields, and high stereoselectivities for β-substituted enones.

The development of novel approaches for the synthesis of α-arylated carbonyl compounds remains a topic of interest in synthetic chemistry. Whereas classical approaches rely on transition-metal-catalyzed couplings of carbonyl-derived enolates with aryl halides or pseudohalides (Scheme 1A),^1^ complementary transition-metal-free methods based on electrophilic aromatic derivatives such as sulfur(IV),^2^ bismuth(V),^3^ iodine(III),^4^ and arynes^5^ have emerged in the last years. Alternative methodologies employing *N*-alkoxyenamines,^6^ enolonium equivalents,^7^ oxy-allyl cations,^8^ and radical-mediated arylations^9^ have also been developed.

**Scheme 1:**
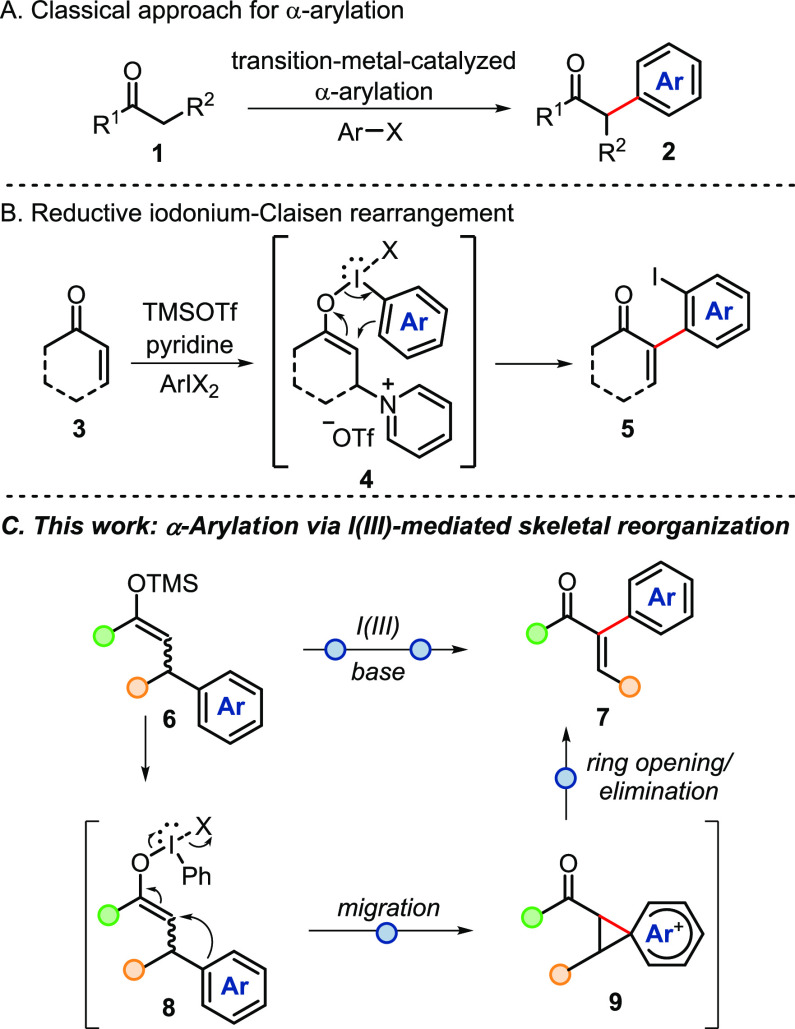
(A) Transition-Metal-Catalyzed Approach for α-Arylation of Carbonyl Compounds, (B) Enone Arylation *via* β-Pyridinium Enolonium Species, and (C) This Work: Enone α-Arylation *via* Iodine(III)-Mediated Aryl Migration/Elimination.

Despite the great progress achieved with transition-metal-free approaches, limitations in achievable substitution patterns and low atom economy still remain as drawbacks. In a recently disclosed elegant contribution by Wengryniuk and coworkers on the transition-metal-free α-arylation of enones, the direct C–H α-arylation occurs via the reductive iodonium Claisen rearrangement of *in-situ*-generated β-pyridinium silyl enol ethers (**4**) and ArI(O_2_CCF_3_)_2_ reagents (Scheme 1B).^10^ Although this method features high atom economy, comparably expedient substrate synthesis, and a broad arene scope, its modularity is limited by the inevitable presence of an *ortho*-iodo substituent and accompanying restrictions in the substitution pattern of the aromatic in addition to the need to prepare the iodoarenes.

As part of our long-standing interest in the rearrangements of high-energy intermediates, we have established methodologies for the iodine(III)-mediated α-arylation^11^ and α-cyclopropanation^12^ of carbonyl compounds through oxidative C–C bond activation and carbocationic rearrangements, respectively. Inspired by the outstanding ability of iodine(III)^13^ in promoting oxidative rearrangements,^14^ we wondered whether this class of reagents could evoke an intramolecular α-arylation of enones. Herein we report a practical transition-metal-free protocol for the formal α-arylation of enones via iodine(III)-mediated aryl migration/elimination.

Encouraged by our previous work on I(III)-mediated rearrangements,^11,12^ we envisioned a process in which intermediate **9** (Scheme 1C), the product of aryl migration on enolonium species **8**, would undergo elimination to form **7**. We therefore started our investigations with the silyl enol ether **6a** as a model substrate and TMSOTf (trimethylsilyl trifluoromethanesulfonate) as the activator for a range of I(III) reagents (Table 1). The commercially available I(III) reagent PIDA (diacetoxyiodo)benzene, PhI(OAc)_2_) gave a promising 50% yield of the desired α-arylated enone (entry 1). Increasing the amount of Et_3_N (required for elimination) from 2 to 4 equiv led to the formation of **7a** in 74% yield (entry 2), and a screening of I(III) reagents revealed PIDA as the optimum oxidant for this transformation (entries 3–5).

**Table 1:**
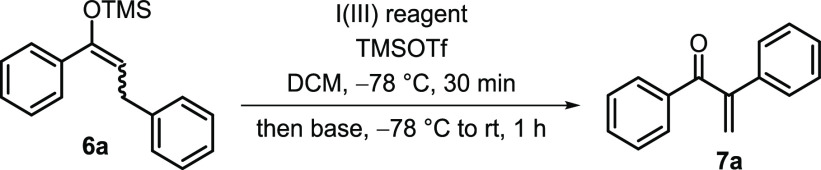
Optimization of the Reaction Conditions^a^

entry	I(III) reagent^b^	TMSOTf (equiv)	base	yield (%)^g^
1	PhI(OAc)_2_	1.2	Et_3_N^c^	50
2	PhI(OAc)_2_	1.2	Et_3_N^d^	74
3	PhIO	1.2	Et_3_N^d^	70
4	PhI(OPiv)_2_	1.2	Et_3_N^d^	56
5	PhI(OCOCF_3_)_2_	1.2	Et_3_N^d^	24
6	PhI(OAc)_2_	1.2	DIPEA^d^^,^^e^	38
7	PhI(OAc)_2_	1.2	NaOH^f^	12
8	PhI(OAc)_2_	1.4	Et_3_N^d^	82^h^

a All screening was performed on 0.2 mmol of **6a** (1 equiv) in 2 mL of DCM (0.1 M).

b 1.2 equiv.

c 2 equiv.

d 4 equiv.

e Alongside 35% β-trifate.

f 1 M, 4 equiv.

g NMR yields.

h Isolated yield. TMSOTf = trimethylsilyl trifluoromethanesulfonate. DIPEA = *N*,*N*-diisopropylethylamine. DCM = dichloromethane.

Switching from Et_3_N to either DIPEA (*N*,*N*-diisopropylethylamine) or NaOH did not lead to any improvement (entries 6 and 7); rather, through the presence of 35% β-triflated, α-arylated ketone, we were able to determine that the observed process likely proceeds *via* the capture of **9** by a triflate anion rather than direct elimination. It is additionally worth mentioning that during the examination of the conditions employing PIDA as the I(III) reagent, we observed the formation of distinct side products (α**-** and β-acetates), generated by competitive reactions with intermediates **8** and **9**.^11,12,15^ Attempting to avoid the formation of these byproducts, we increased the amount of the activator TMSOTf, which led to an improved isolated yield (82%) of the α-arylated enone **7a** (entry 8).

With the optimized conditions in hand, we investigated the impact of electronics and sterics on the employed silyl enol ethers (Scheme 2). We first evaluated different substituents on the benzoyl moiety (Scheme 2a). Electron-donating, -withdrawing, and -neutral groups were all well tolerated, and the products **7a**–**j** were obtained in good yields regardless of the position of the substituent. Furthermore, a silyl enol ether bearing a disubstituted aromatic ring was also susceptible to rearrangement in high yield (**7i**). Substrates bearing more elaborate aromatic rings such as 2-naphthyl (**7k**) and 1,3-benzodioxol-5-yl (**7l**) were also successfully transformed, as was 2-thienoyl-derived substrate (**7m**). In addition, an alkyl α-arylated enone **7n** and an enal **7o** were formed in 86 and 60% yield, respectively.^16^

**Scheme 2:**
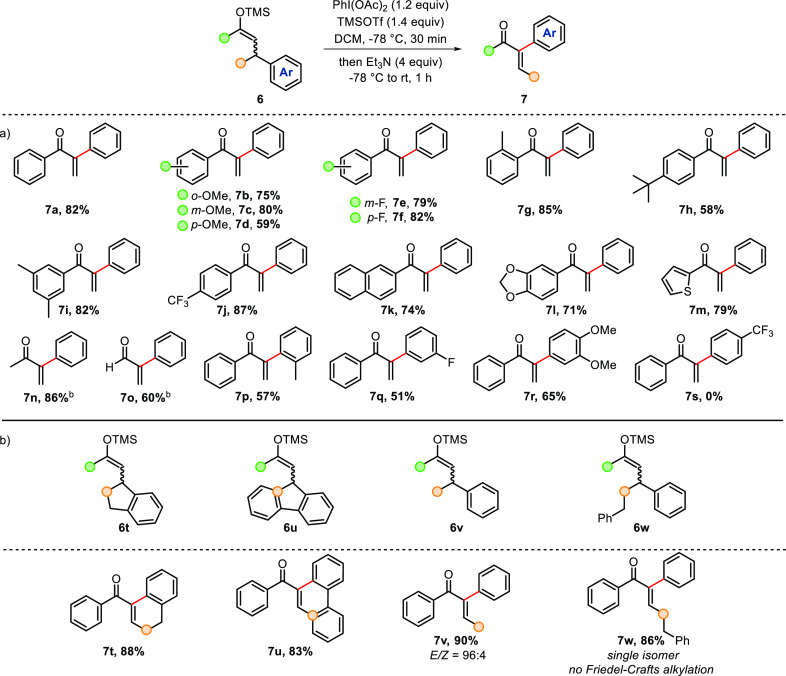
Scope of I(III)-Mediated α-Arylation of Enones All yields refer to pure, isolated products, unless otherwise stated. NMR yields were determined using mesitylene as an internal standard.

Next, we evaluated the migration of substituted aryl groups. The rearrangement of an *o*-tolyl moiety afforded the product **7p** in 57% yield, whereas the migration of a *m*-fluoro derivative provided the enone **7q** in 51% yield. Additionally, the migration of a disubstituted aromatic group was accomplished effectively, yielding enone **7r** in 65%. Unfortunately, the shift of an electron-poor aryl group such as *p*-CF_3_C_6_H_4_ proved fruitless as a consequence of its diminished migratory ability. Instead, we obtained the corresponding β-arylated, α,β-unsaturated ketone as a major product under standard conditions.

Interestingly, we found that substrates with fused rings underwent I(III)-mediated aryl migration/ring expansion/elimination, leading to α-arylated enones **7t** and **7u** in high yields (Scheme 2b). Gratifyingly, the formal α-arylation of β-substituted enones proceeded readily, giving the desired products **7v** and **7w** in excellent yields and with excellent stereoselectivities. (See the Supporting Information for a rationalization of this selectivity.) It is worth highlighting that the product **7w** proved more reactive to aryl migration/elimination than to a competitive intramolecular Friedel–Crafts reaction. In the case of an *iso*-propyl-substituted silyl enol ether (**6x**), we found that 2,3-dihydrofuran **7x** was formed in 62% yield (Scheme 3a). We presume that this heterocycle was generated through a phenyl migration/Wagner–Meerwein rearrangement/cyclization sequence.

**Scheme 3:**
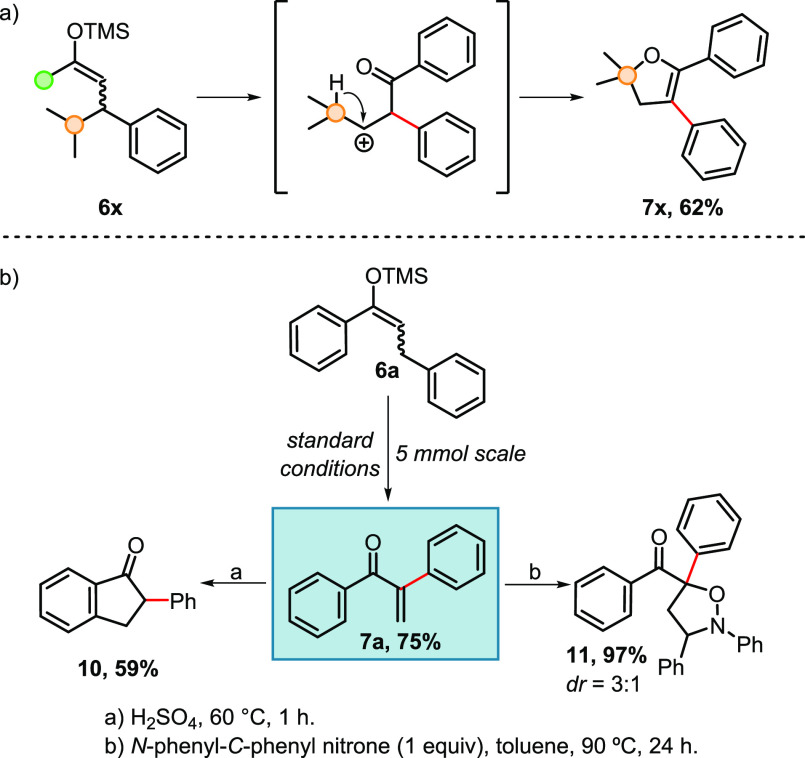
Occurrence of a Cyclization Product and Functionalizations of α-Arylated Enone **7a**

To explore the synthetic utility of the new method, we chose product **7a** for further derivatization (Scheme 3b). Scale up (5 mmol) of its preparation could be achieved without a significant decrease in yield. The enone **7a** subsequently readily underwent Nazarov cyclization or a (3 + 2)-cycloaddition reaction. The synthesis of 2-phenyl-1-indanone (**10**) was accomplished in 59% yield, whereas the cycloaddition of **7a** with *N*-phenyl-*C*-phenyl nitrone furnished the desired isoxazolidine **11** in an excellent yield of 97%.

In summary, we have developed a skeletal rearrangement-based methodology for the α-arylation of enones. The use of I(III) to mediate the aryl migration/elimination enabled the formation of a series of α-arylated enones in good yields under mild conditions. Furthermore, we demonstrated that our method is suitable for substrates bearing fused rings, promoting aryl migration/ring expansion/elimination, as well as for β-substituted silyl enol ethers, giving high yields and excellent stereoselectivities.

## Supporting Information Available

Experimental procedures, ^1^H and ^13^C NMR spectra, and characterization data of compounds (PDF)
