# Supplementary material for: Formal Enone α-Arylation via I(III)-Mediated Aryl Migration/Elimination
Source: Org Lett. 2021 Feb 26;23(6):2094–8. doi: 10.1021/acs.orglett.1c00251 (PMC7985840; doi:10.1021/acs.orglett.1c00251)
Supplement: Supplementary file 1 — ol1c00251_si_001.pdf [file ol1c00251_si_001.pdf]

Formal Enone α-Arylation *via* I(III)-mediated Aryl Migration/Elimination

Bruna S. Martins,‡ Daniel Kaiser,‡ Adriano Bauer, Irmgard Tiefenbrunner, and Nuno Maulide*
Institute of Organic Chemistry, University of Vienna, Währinger Straße 38, 1090 Vienna (Austria)
E-Mail: nuno.maulide@univie.ac.at, Homepage: http://maulide.univie.ac.at

Table of Contents

1. General Information S2
2. Experimental Procedures S3
2.1. Preparation of aryl ketones S3
2.2. Synthesis of silyl enol ethers S5
2.3. General procedure for the aryl migration/elimination S15
2.4. General procedure for 5 mmol scale of **7a** S26
2.5. Nazarov reaction S26
2.6. (3+2)-Cycloaddition reaction S27
3. Rationalization of stereochemical outcome (7v, 7w) S28
4. NMR Spectra S29
5. References S67

1. General Information

Unless otherwise stated, all glassware was flame-dried before use and all reactions were performed under an atmosphere of argon. All solvents were distilled from appropriate drying agents prior to use or, if purchased in anhydrous form, used as received. All reagents were used as received from commercial suppliers, unless otherwise stated. Reaction progress was monitored by thin layer chromatography (TLC) performed on aluminum plates coated with silica gel F254 with 0.2 mm thickness. Flash column chromatography was performed using silica gel 60 (230-400 mesh, Merck and co.). Neat infrared spectra were recorded using a Perkin-Elmer Spectrum 100 FT-IR spectrometer. Wavenumbers (v_{max}) are reported in cm^{-1}. Mass spectra were obtained using a Finnigan MAT 8200 or (70 eV) or an Agilent 5973 (70 eV) spectrometer, using electrospray ionization (ESI). All ^{1}H NMR, ^{13}C NMR, ^{19}F NMR spectra were recorded using a Bruker AV-400, AV-500, AV-600 or AV-700 spectrometer at 300K. Chemical shifts are given in parts per million (ppm, δ), referenced to the solvent peak of $CDCl_3$, defined at δ = 7.26 ppm (^{1}H NMR) and δ = 77.16 (^{13}C NMR). Coupling constants *J* are quoted in Hz. ^{1}H NMR splitting patterns are designated as singlet (s), doublet (d), triplet (t), quartet (q) as they appeared in the spectrum. If the appearance of a signal differs from the expected splitting pattern, the observed pattern is designated as apparent (app). Splitting patterns that could not be interpreted or easily visualized are designated as multiplet (m) or broad (br).

2. Experimental Procedures

2.1. Preparation of aryl ketones

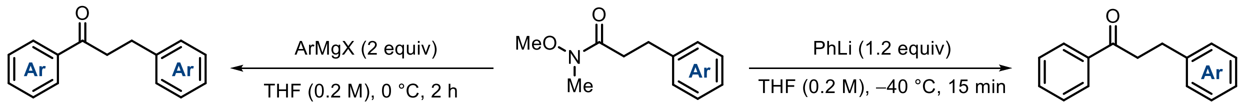

The precursors for the synthesis of the silyl enol ethers were prepared following either of the procedures shown above. The Weinreb amides were synthesized according to a known procedure.[1]

A) *Grignard addition*: To a solution of the appropriate Weinreb amide in anhydrous tetrahydrofuran (0.2 M) at 0 °C was added the aryl Grignard reagent (2 equiv) dropwise. The reaction was kept stirring at 0 °C for 2 h. After this time, a saturated aqueous solution of ammonium chloride was cautiously added to the reaction mixture and the layers were separated. The aqueous layer was extracted with ethyl acetate (three times). The combined organic layers were dried over anhydrous magnesium sulfate, filtered and the solvent was evaporated under reduced pressure. The crude mixture was purified by flash column chromatography on silica gel to afford the desired products.

B) *Phenyllithium addition*: To a solution of the appropriate Weinreb amide in anhydrous tetrahydrofuran (0.2 M) at –40 °C was added phenyllithium (1.9 M in dibutyl ether, 1.2 equiv) dropwise. The reaction was kept stirring at –40 °C for a further 15 min. After this time, an excess of methanol was added dropwise, so as not to raise the temperature of the solution. The resulting mixture was poured onto a saturated aqueous solution of ammonium chloride and the layers were separated. The aqueous layer was extracted with ethyl acetate (three times). The combined organic layers were dried over anhydrous magnesium sulfate, filtered and the solvent was evaporated under reduced pressure. The crude mixture was purified by flash column chromatography on silica gel to afford the desired products.

*With the exception of ketone **1u** (see below), all ketones have been reported in the literature, for which reason the data is not reproduced here.*

2-(9H-fluoren-9-yl)-1-phenylethan-1-one (1u)

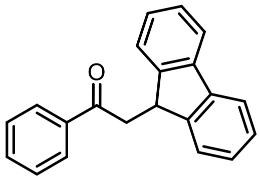

$C_{21}H_{16}O$
MW: 284.36
Yield: 54%
yellowish oil

Synthesized following general procedure **B**, from 2-(9H-fluoren-9-yl)-*N*-methoxy-*N*-methylacetamide using phenyllithium (3 equiv). Purification on silica gel (heptane/ethyl acetate 90:10) gave the title compound in 54% yield.

^{1}H NMR (600 MHz, $CDCl_3$): δ 8.00 (d, *J* = 7.2 Hz, 2H), 7.79 (d, *J* = 7.8 Hz, 2H), 7.59 – 7.57 (m, 1H), 7.52 (d, 7.8 Hz, 2H), 7.48 – 7.46 (m, 2H), 7.40 – 7.37 (m, 2H), 7.30 – 7.26 (m, 2H), 4.80 (t, *J* = 6.6 Hz, 1H), 3.47 (d, *J* = 6.6 Hz, 2H) ppm.

^{13}C NMR (151 MHz, $CDCl_3$): δ 199.0, 147.3 (2C), 141.0 (2C), 137.1, 133.4, 128.8 (2C), 128.3 (2C), 127.4 (2C), 127.3 (2C), 124.8 (2C), 120.0 (2C), 43.6, 42.7 ppm.

IR (neat) ν_{max}: 3063, 3038, 2956, 2892, 2854, 1683, 1597, 1580, 1476, 1447, 1352, 1275, 1198, 1179, 1159, 982, 749, 739, 689, 589 cm^{-1}.

HRMS (ESI$^+$) *m/z*: $[M+Na]^+$ calcd for $C_{21}H_{16}ONa$ 307.1093; found 307.1090.

2.2. Synthesis of silyl enol ethers

General Procedure A

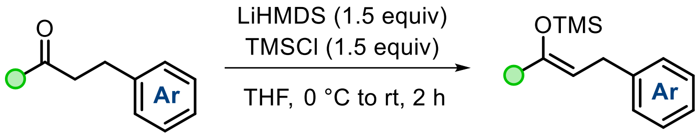

To a cooled solution of lithium bis(trimethylsilyl)amide (1 M in tetrahydrofuran, 1.5 equiv) at 0 °C in a flame-dried Schlenk flask, the ketone in anhydrous tetrahydrofuran (0.5 M) was added dropwise. After stirring at the same temperature for 30 min, chlorotrimethylsilane (1.5 equiv) was added dropwise and the resulting solution was allowed to warm to ambient temperature and stirred for another 2 h. After this time, the reaction mixture was transferred to a round-bottom flask using dichloromethane and the solvents were removed under reduced pressure. The crude residue was subsequently filtered through a short column of silica gel. Inseparable TMSOTMS was then removed under high vacuum over the course of several hours to afford the pure product.

General Procedure B

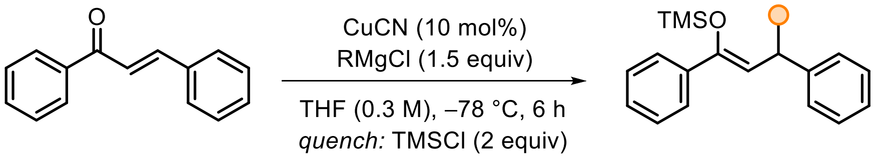

Copper cyanide (10 mol%) was added to a flame-dried Schlenk flask followed by anhydrous tetrahydrofuran (0.3 M) and the mixture was stirred at room temperature for 10 min. The mixture was cooled to –78°C and subsequently the appropriate Grignard reagent (1.5 equiv) was added dropwise. The reaction mixture was stirred at –78°C for an additional 10 min. After this time, a solution of *trans*-chalcone (1.0 equiv) in anhydrous tetrahydrofuran (1 M) was added dropwise over 1 h. The reaction mixture was stirred for 6 h and the resulting enolate was quenched by the addition of chlorotrimethylsilane (2 equiv), allowing the system to reach room temperature while stirring for additional 1 h. After this time, saturated aqueous solution of ammonium chloride was added to the reaction mixture and the layers were separated. The aqueous layer was extracted with ethyl acetate (three times). The combined organic layers were dried over magnesium sulfate, filtered and the solvent was evaporated under reduced pressure.

(*Z*)-((1,3-diphenylprop-1-en-1-yl)oxy)trimethylsilane (6a)

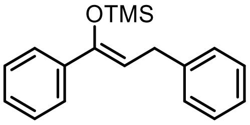

$C_{18}H_{22}OSi$
MW: 282.46

Synthesized following general procedure **A**, from commercially available 1,3-diphenylpropan-1-one. Purification by filtration over silica gel (heptane/ethyl acetate 95:5) gave the title compound in quantitative yield. All analytical data were in good accordance with those reported in the literature.[2]

***(Z)*-((1-(2-methoxyphenyl)-3-phenylprop-1-en-1-yl)oxy)trimethylsilane (6b)**

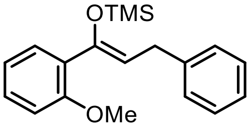

$C_{19}H_{24}O_2Si$
MW: 312.48

Synthesized following general procedure **A**, from 1-(2-methoxyphenyl)-3-phenylpropan-1-one.[3] Purification by filtration over silica gel (toluene/dichloromethane 80:20) gave the title compound in 56% yield. All analytical data were in good accordance with those reported in the literature.[4]

(*Z*)-((1-(3-methoxyphenyl)-3-phenylprop-1-en-1-yl)oxy)trimethylsilane (6c)

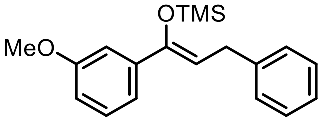

$C_{19}H_{24}O_2Si$
MW: 312.48
Yield: 83%
yellowish oil

Synthesized following general procedure **A**, from 1-(3-methoxyphenyl)-3-phenylpropan-1-one. Purification by filtration over silica gel (heptane/ethyl acetate 97:3) gave the title compound in 83% yield.

^{1}H NMR (700 MHz, $CDCl_3$): δ 7.31 – 7.29 (m, 2H), 7.26 (d, *J* = 8.0 Hz, 2H), 7.23 – 7.19 (m, 2H), 7.10 (d, *J* = 7.7 Hz, 1H), 7.06 – 7.05 (m, 1H), 6.81 (dd, *J* = 8.0, 2.4 Hz, 1H), 5.44 (t, *J* = 7.2 Hz, 1H), 3.82 (s, 3H), 3.57 (d, *J* = 7.2 Hz, 2H), 0.18 (s, 9H) ppm.

^{13}C NMR (176 MHz, $CDCl_3$): δ 159.6, 149.7, 141.5, 140.6, 129.2, 128.6 (2C), 128.5 (2C), 126.0, 118.2, 113.5, 111.1, 110.2, 55.3, 32.5, 0.82 (3C) ppm.

IR (neat) ν_{max}: 3082, 3061, 3027, 3001, 2956, 2901, 2833 cm^{-1}.

HRMS (ESI$^+$) *m/z*: $[M+H]^+$ calcd for $C_{19}H_{25}O_2Si$ 313.1618; found 313.1623.

(*Z*)-((1-(4-methoxyphenyl)-3-phenylprop-1-en-1-yl)oxy)trimethylsilane (6d)

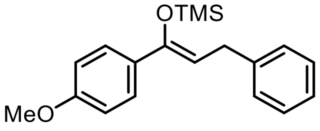

$C_{19}H_{24}O_2Si$
MW: 312.48

Synthesized following general procedure **A**, from 1-(4-methoxyphenyl)-3-phenylpropan-1-one.[5] Purification by flash column chromatography over silica gel (toluene/dichloromethane 80:20) gave the title compound in 87% yield. All analytical data were in good accordance with those reported in the literature.[4]

(*Z*)-((1-(3-fluorophenyl)-3-phenylprop-1-en-1-yl)oxy)trimethylsilane (6e)

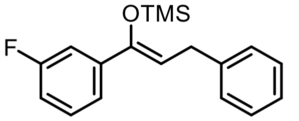

$C_{18}H_{21}FOSi$
MW: 300.45

Synthesized following general procedure **A**, from 1-(3-fluorophenyl)-3-phenylpropan-1-one.[6] Purification by flash column chromatography over silica gel (heptane/ethyl acetate 95:5) gave the title compound in quantitative yield. All analytical data were in good accordance with those reported in the literature.[4]

(*Z*)-((1-(4-fluorophenyl)-3-phenylprop-1-en-1-yl)oxy)trimethylsilane (6f)

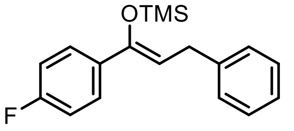

$C_{18}H_{21}OFSi$
MW: 300.45

Synthesized following general procedure **A**, from 1-(4-fluorophenyl)-3-phenylpropan-1-one. Purification by flash column chromatography over silica gel (heptane/ethyl acetate 98:2) gave the title compound in 92% yield. All analytical data were in good accordance with those reported in the literature.[4]

trimethyl((3-phenyl-1-(*o*-tolyl)prop-1-en-1-yl)oxy)silane (6g)

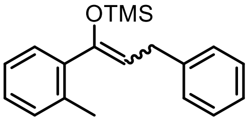

$C_{19}H_{24}OSi$
MW: 296.48
Yield: >95%, *Z*/*E*: 3:1
yellowish oil

Synthesized following general procedure **A**, from 3-phenyl-1-(*o*-tolyl)propan-1-one. Purification by filtration over silica gel (heptane/ethyl acetate 98:2) gave the title compound in quantitative yield.

^{1}H NMR (500 MHz, $CDCl_3$): *Z*/*E* = 3:1. δ 7.33 – 7.26 (m, 6H total), 7.24 – 7.18 (m, 4H total), 7.17 – 7.12 (m, 3H total), 5.25 (t, *J* = 7.8 Hz, 0.3H, minor isomer), 5.01 (t, *J* = 7.2 Hz, 1H, major isomer), 3.59

(d, J = 7.2 Hz, 2H, major isomer), 3.19 (d, J = 7.8 Hz, 0.6H, minor isomer), 2.41 (s, 3H, major isomer), 2.38 (s, 1H, minor isomer), 0.13 (s, 3H, minor isomer), 0.03 (s, 9H, major isomer) ppm.

^{13}C NMR (126 MHz, CDCl$_3$): *Z*/*E* = 3:1. δ 151.1, 150.6, 142.0, 141.9, 139.5, 137.1, 137.0, 136.4, 130.3, 130.31, 129.7, 129.2, 128.5 (2C), 128.46 (2C), 128.4, 128.26, 128.2, 128.0, 125.9, 125.8, 125.4, 125.39, 112.0, 109.0, 33.7, 31.9, 20.5, 19.8, 0.5, 0.4 (3C) ppm.

IR (neat) ν$_{max}$: 3083, 3062, 3026, 2958, 2925, 2900, 2858 cm^{-1}.

HRMS (ESI$^+$) *m/z*: [M+H]$^+$ calcd for $C_{19}H_{25}OSi$ 297.1669; found 297.1668.

(*Z*)-((1-(4-(*tert*-butyl)phenyl)-3-phenylprop-1-en-1-yl)oxy)trimethylsilane (6h)

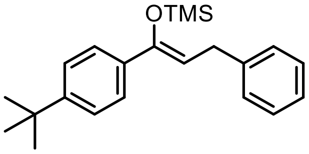

$C_{22}H_{30}OSi$
MW: 338.57

Synthesized following general procedure **A**, from 1-(4-(*tert*-butyl)phenyl)-3-phenylpropan-1-one. Purification by flash column chromatography over silica gel (heptane/ethyl acetate 98:2) gave the title compound in quantitative yield. All analytical data were in good accordance with those reported in the literature.[4]

(*Z*)-((1-(3,5-dimethylphenyl)-3-phenylprop-1-en-1-yl)oxy)trimethylsilane (6i)

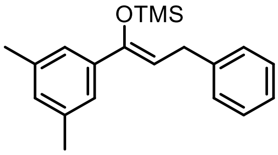

$C_{20}H_{26}OSi$
MW: 310.51
Yield: 61%
colourless oil

Synthesized following general procedure **A**, from 1-(3,5-dimethylphenyl)-3-phenylpropan-1-one. Purification by flash column chromatography over silica gel (heptane/ethyl acetate 98:2) gave the title compound in 61% yield.

^{1}H NMR (600 MHz, CDCl$_3$): δ 7.31 – 7.25 (m, 4H), 7.20 – 7.18 (m, 1H), 7.12 (s, 2H), 6.90 (s, 1H), 5.40 (t, J = 7.1 Hz, 1H), 3.55 (d, J = 7.1 Hz, 2H), 2.30 (s, 6H), 0.16 (s, 9H) ppm.

^{13}C NMR (151 MHz, CDCl$_3$): δ 150.0, 141.8, 138.9, 137.6, 129.4, 128.6 (2C), 128.5 (3C), 125.9, 123.6 (2C), 109.6, 32.5, 21.5 (2C), 0.8 (3C) ppm.

IR (neat) ν$_{max}$: 3084, 3005, 1730, 1645, 1323, 1251, 1108, 1004, 838, 755, 729, 696, 648 cm^{-1}.

HRMS (ESI$^+$) *m/z*: [M+H]$^+$ calcd for $C_{20}H_{27}OSi$ 311.1826; found 311.1828.

(*Z*)-trimethyl((3-phenyl-1-(4-(trifluoromethyl)phenyl)prop-1-en-1-yl)oxy)silane (6j)

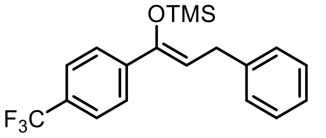

$C_{19}H_{21}F_3OSi$
MW: 350.46

Synthesized following general procedure **A**, from 3-phenyl-1-(4-(trifluoromethyl)phenyl)propan-1-one.[7] Purification by flash column chromatography over silica gel (toluene/dichloromethane 80:20) gave the title compound in 28% yield. All analytical data were in good accordance with those reported in the literature.[4]

(*Z*)-trimethyl((1-(naphthalen-2-yl)-3-phenylprop-1-en-1-yl)oxy)silane (6k)

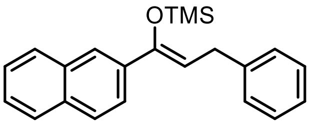

$C_{22}H_{24}OSi$
MW: 332.52
Yield: >95%
white solid

Synthesized following general procedure **A**, from 1-(naphthalen-2-yl)-3-phenylpropan-1-one. Purification by flash column chromatography over silica gel (heptane/ethyl acetate 98:2) gave the title compound in quantitative yield.

^{1}H NMR (700 MHz, $CDCl_3$): δ 7.75 (s, 1H), 7.63 – 7.60 (m, 2H), 7.56 (d, *J* = 8.6 Hz, 1H), 7.44 (dd, *J* = 8.6, 1.7 Hz, 1H), 7.28 – 7.23 (m, 2H), 7.12 – 7.10 (m, 4H), 7.08 – 7.00 (m, 1H), 5.39 (t, *J* = 7.1 Hz, 1H), 3.44 (d, *J* = 7.1 Hz, 2H), 0.00 (s, 9H) ppm.

^{13}C NMR (151 MHz, $CDCl_3$): δ 149.9, 141.6, 136.4, 133.3, 133.1, 128.61 (2C), 128.6 (2C), 128.4, 127.8, 127.7, 126.3, 126.1, 126.0, 124.4, 124.0, 110.7, 32.7, 0.8 (3C) ppm.

IR (neat) v_{max}: 3082, 3059, 3026, 2958, 2928, 2899, 2838 cm^{-1}.

MP: 38 – 41 °C.

HRMS (ESI$^+$) *m/z*: $[M+H]^+$ calcd for $C_{22}H_{25}OSi$ 333.1669; found 333.1659.

(*Z*)-((1-(benzo[d][1,3]dioxol-5-yl)-3-phenylprop-1-en-1-yl)oxy)trimethylsilane (6l)

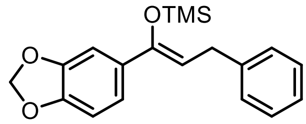

$C_{19}H_{22}O_3Si$
MW: 326.47

Synthesized following general procedure **A**, from 1-(benzo[d][1,3]dioxol-5-yl)-3-phenylpropan-1-one. Purification by flash column chromatography over silica gel (heptane/ethyl acetate 98:2) gave the title compound in 75% yield. All analytical data were in good accordance with those reported in the literature.[4]

***(Z)*-trimethyl((3-phenyl-1-(thiophen-2-yl)prop-1-en-1-yl)oxy)silane (6m)**

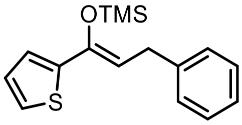

$C_{16}H_{20}OSSi$
MW: 288.48

Synthesized following general procedure **A**, from 3-phenyl-1-(thiophen-2-yl)propan-1-one.[8] Purification by flash column chromatography over silica gel (toluene/dichloromethane 80:20) gave the title compound in 97% yield. All analytical data were in good accordance with those reported in the literature.[4]

trimethyl((4-phenylbut-2-en-2-yl)oxy)silane (6n)

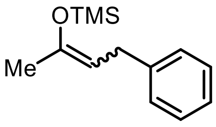

$C_{13}H_{20}OSi$
MW: 220.39
Z/E ~ 1.6:1

Synthesized following a reported procedure,[9] from commercially available 4-phenyl-3-buten-2-one. Purification by filtration over silica gel (heptane/toluene 50:50) gave the title compound in 37% yield. All analytical data were in good accordance with those reported in the literature.[9]

***(E)*-trimethyl((3-phenylprop-1-en-1-yl)oxy)silane (6o)**

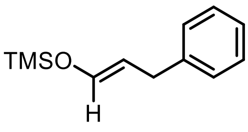

$C_{12}H_{18}OSi$
MW: 206.36

Synthesized following a reported procedure,[9] from commercially available *trans*-cinnamaldehyde. Purification by filtration over silica gel (heptane/toluene 50:50) gave the title compound in 47% yield. All analytical data were in good accordance with those reported in the literature.[9]

(*Z*)-trimethyl((1-phenyl-3-(*o*-tolyl)prop-1-en-1-yl)oxy)silane (6p)

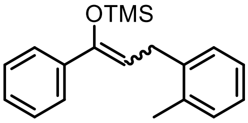

$C_{19}H_{24}OSi$
MW: 296.48
Yield: >95%, *Z*:*E* = 14:1
colourless oil

Synthesized following general procedure **A**, from 1-phenyl-3-(*o*-tolyl)propan-1-one. Purification by flash column chromatography over silica gel (heptane/ethyl acetate 98:2) gave the title compound in quantitative yield.

^{1}H NMR (500 MHz, CDCl$_3$): *Z*:*E* 14:1 (data for major isomer) δ 7.46 (d, *J* = 7.5 Hz, 2H), 7.27 – 7.24 (m, 2H), 7.20 (t, *J* = 7.3 Hz, 2H), 7.12 – 7.07 (m, 3H), 5.32 (t, *J* = 7.1 Hz, 1H), 3.49 (d, *J* = 7.1 Hz, 2H), 2.30 (s, 3H), 0.13 (s, 9H) ppm.

^{13}C NMR (126 MHz, CDCl$_3$): *Z*:*E* 14:1 (data for major isomer) δ 149.8, 139.7, 139.1, 136.5, 130.2, 128.7, 128.2 (2C), 127.7, 126.2, 126.1, 125.6 (2C), 109.3, 30.4, 19.6, 0.8 (3C) ppm.

IR (neat) ν_{max}: 3083, 3062, 3026, 2958, 2925, 2900 cm^{-1}.

HRMS (ESI$^+$) *m/z*: [M+Na]$^+$ calcd for $C_{19}H_{24}ONaSi$ 319.1489; found 319.1483.

(*Z*)-((3-(3-fluorophenyl)-1-phenylprop-1-en-1-yl)oxy)trimethylsilane (6q)

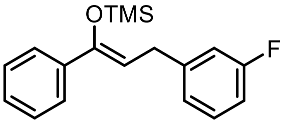

$C_{18}H_{21}OFSi$
MW: 300.45
Yield: 94%
colourless oil

Synthesized following general procedure **A**, from 3-(3-fluorophenyl)-1-phenylpropan-1-one. Purification by flash column chromatography over silica gel (heptane/ethyl acetate 98:2) gave the title compound in 94% yield.

^{1}H NMR (500 MHz, CDCl$_3$): δ 7.35 (d, *J* = 7.6 Hz, 2H), 7.18 – 7.15 (m, 2H), 7.13 – 7.07 (m, 2H), 6.89 (d, *J* = 7.6 Hz, 1H), 6.82 (d, *J* = 9.8 Hz, 1H), 6.76 – 6.71 (m, 1H), 5.24 (t, *J* = 7.2 Hz, 1H), 3.41 (d, *J* = 7.2 Hz, 2H), 0.00 (s, 9H) ppm.

^{13}C NMR (126 MHz, CDCl$_3$): δ 163.1 (d, *J* = 245.0 Hz), 150.5, 144.2 (d, *J* = 7.0 Hz), 138.9, 129.8 (d, *J* = 8.3 Hz), 128.3 (2C), 128.0, 125.8 (2C), 124.1 (d, *J* = 2.7 Hz), 115.4 (d, *J* = 21.0 Hz), 112.9 (d, *J* = 21.0 Hz), 109.0, 32.2, 0.8 (3C) ppm.

^{19}F NMR (471 MHz, CDCl$_3$): δ –113.8 ppm.

IR (neat) ν_{max}: 3083, 3058, 3036, 2959, 2899, 2841 cm^{-1}.

HRMS (ESI+) *m/z*: [M+Na]+ calcd for $C_{18}H_{21}OFNaSi$ 323.1238; found 323.1232.

(Z)-((3-(3,4-dimethoxyphenyl)-1-phenylprop-1-en-1-yl)oxy)trimethylsilane (6r)

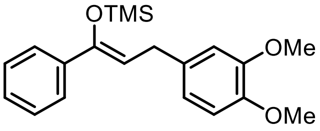

$C_{20}H_{26}O_3Si$
MW: 342.51

Synthesized following general procedure **A**, from 3-(3,4-dimethoxyphenyl)-1-phenylpropan-1-one. Purification by flash column chromatography over silica gel (heptane/ethyl acetate 98:2) gave the title compound in quantitative yield. All analytical data were in good accordance with those reported in the literature.[4]

(*Z*)-trimethyl((1-phenyl-3-(4-(trifluoromethyl)phenyl)prop-1-en-1-yl)oxy)silane (6s)

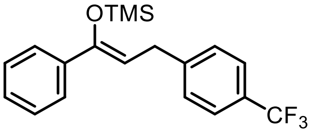

$C_{19}H_{21}OF_3Si$
MW: 350.46

Synthesized following general procedure **A**, from 1-phenyl-3-(4-(trifluoromethyl)phenyl)propan-1-one. Purification by flash column chromatography over silica gel (heptane/ethyl acetate 98:2) gave the title compound in 63% yield. All analytical data were in good accordance with those reported in the literature.[4]

***(Z)*-((2-(2,3-dihydro-1H-inden-1-yl)-1-phenylvinyl)oxy)trimethylsilane (6t)**

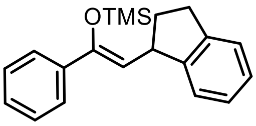

$C_{20}H_{24}OSi$
MW: 308.50
Yield: 93%
colourless oil

Synthesized following general procedure **A**, from 2-(2,3-dihydro-1H-inden-1-yl)-1-phenylethan-1-one.[10] No purification was necessary (product decomposed on silica) and the title compound was obtained in 93% yield.

^{1}H NMR (400 MHz, CDCl$_3$): δ 7.54 – 7.52 (m, 2H), 7.34 – 7.16 (m, 7H), 5.30 (d, *J* = 9.7 Hz, 1H), 4.30 (td, *J* = 9.8, 9.2 Hz, 1H), 3.01 – 2.90 (m, 2H), 2.47 – 2.39 (m, 1H), 1.88 – 1.76 (m, 1H), 0.18 (s, 9H) ppm.

^{13}C NMR (101 MHz, CDCl$_3$): δ 150.0, 147.2, 144.1, 139.2, 128.2 (2C), 127.8, 126.5, 126.4, 125.8 (2C), 124.5, 124.5, 114.5, 42.5, 34.2, 32.0, 0.8 (3C) ppm.

IR (neat) ν_{max}: 3064, 3021, 2956, 1644, 1445, 1252, 1053, 840, 754 cm^{-1}.

HRMS (ESI$^+$) *m/z*: $[M+Na]^+$ calcd for $C_{20}H_{24}OSiNa$ 331.1494; found 331.1483.

(*Z*)-((2-(9H-fluoren-9-yl)-1-phenylvinyl)oxy)trimethylsilane (6u)

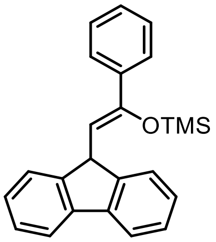

$C_{24}H_{24}OSi$
MW: 356.54
Yield: 83%
white solid

Synthesized following general procedure **A**, from 2-(9H-fluoren-9-yl)-1-phenylethan-1-one using Et_3N (10 equiv) instead of LiHMDS. Purification by flash column chromatography over silica gel (heptane/ethyl acetate 97:3) gave the title compound in 83% yield.

^{1}H NMR (600 MHz, $CDCl_3$): δ 7.77 (d, *J* = 7.5 Hz, 2H), 7.56 – 7.52 (m, 4H), 7.38 (t, *J* = 7.4 Hz, 2H), 7.32 – 7.30 (m, 4H), 7.28 – 7.25 (m, 1H), 5.15 (d, *J* = 9.8 Hz, 1H), 5.09 (d, *J* = 9.8 Hz, 1H), 0.28 (s, 9H) ppm.

^{13}C NMR (151 MHz, $CDCl_3$): δ 152.6, 147.7 (2C), 141.2 (2C), 139.0, 128.3 (2C), 128.0, 127.3 (2C), 127.2 (2C), 125.9 (2C), 125.4 (2C), 119.9 (2C), 110.1, 45.9, 0.99 (3C) ppm.

IR (neat) ν_{max}: 3063, 3035, 3017, 2970, 2929, 2894 cm^{-1}.

MP: 87 – 90 °C.

HRMS (ESI$^+$) *m/z*: $[M+Na]^+$ calcd for $C_{24}H_{24}OSiNa$ 379.1489; found 379.1491.

(*Z*)-((1,3-diphenylbut-1-en-1-yl)oxy)trimethylsilane (6v)

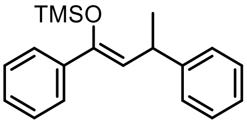

$C_{19}H_{24}OSi$
MW: 296.48
>95%, *Z*/*E* = 18:1
colourless oil

Synthesized following general procedure **A**, from 1,3-diphenylbutan-1-one. Purification by flash column chromatography over silica gel (heptane/ethyl acetate 97:3) gave the title compound in quantitative yield. All analytical data were in good accordance with those reported in the literature.[11]

(*Z*)-trimethyl((1,3,5-triphenylpent-1-en-1-yl)oxy)silane (6w)

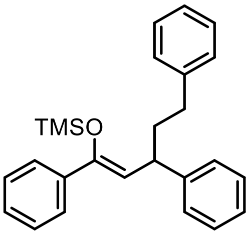

$C_{26}H_{30}OSi$
MW: 386.61
Yield: 40%
colourless oil

Synthesized following general procedure **B**, from commercially available *trans*-chalcone and phenethylmagnesium chloride. Purification by flash column chromatography over silica gel (heptane/ethyl acetate 97:3) gave the title compound in 40% yield.

^{1}H NMR (500 MHz, CDCl$_3$): δ 7.43 (d, *J* = 7.6 Hz, 2H), 7.29 – 7.19 (m, 10H), 7.16 – 7.11 (m, 3H), 5.37 (d, *J* = 9.7 Hz, 1H), 3.82 – 3.77 (m, 1H), 2.61 – 2.54 (m, 2H), 2.08 – 1.93 (m, 2H), 0.00 (s, 9H) ppm.

^{13}C NMR (126 MHz, CDCl$_3$): δ 149.5, 145.6, 142.6, 139.4, 128.7 (2C), 128.6 (2C), 128.4 (2C), 128.2 (2C), 127.8, 127.7 (2C), 126.1, 126.0 (2C), 125.8, 115.0, 41.9, 39.5, 34.0, 0.79 (3C) ppm.

IR (neat) ν_{max}: 3083, 3060, 3026, 2954, 2939, 2856 cm^{-1}.

HRMS (ESI$^+$) *m/z*: [M+H]$^+$ calcd for $C_{26}H_{31}OSi$ 387.2139; found 387.2140.

(*Z*)-trimethyl((4-methyl-1,3-diphenylpent-1-en-1-yl)oxy)silane (6x)

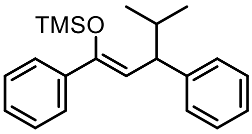

$C_{21}H_{28}OSi$
MW: 324.54
Yield: 38%
colourless oil

Synthesized following general procedure **B**, from commercially available *trans*-chalcone and *iso*propylmagnesium chloride. Purification by flash column chromatography over silica gel (heptane/ethyl acetate 97:3) gave the title compound in 38% yield.

^{1}H NMR (600 MHz, CDCl$_3$): δ 7.42 – 7.40 (m, 2H), 7.26 – 7.22 (m, 4H), 7.20 – 7.17 (m, 3H), 7.13 – 7.10 (m, 1H), 5.41 (d, *J* = 10.0 Hz, 1H), 3.46 (dd, *J* = 10.0, 8.0 Hz, 1H), 1.92 – 1.84 (m, 1H), 0.92 (d, *J* = 6.7 Hz, 3H), 0.77 (d, *J* = 6.7 Hz, 3H), 0.00 (s, 9H) ppm.

^{13}C NMR (151 MHz, CDCl$_3$): δ 149.8, 145.1, 139.7, 128.3 (2C), 128.2 (2C), 128.1 (2C), 127.7, 126.1 (2C), 125.8, 113.7, 49.4, 34.8, 21.2, 20.4, 0.89 (3C) ppm.

IR (neat) ν_{max}: 3083, 3059, 3026, 2956, 2929, 2902, 2869 cm^{-1}.

HRMS (ESI$^+$) *m/z*: [M+H]$^+$ calcd for $C_{21}H_{29}OSi$ 325.1982; found 325.1981.

2.3. General procedure for the aryl migration/elimination

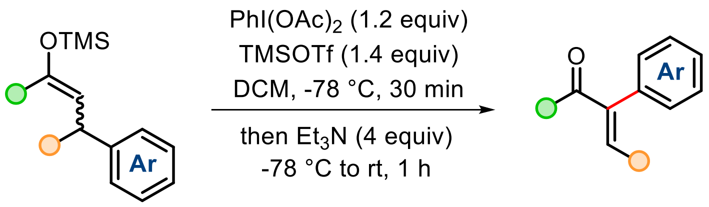

All reactions were performed on a 0.2 mmol scale

Iodosobenzene diacetate (79.7 mg, 0.24 mmol, 1.2 equiv) was placed in a flame-dried Schlenk tube and dichloromethane (0.1 M with respect to the silyl enol ether) was added. The suspension was cooled to 0 °C, after which trimethylsilyl trifluoromethanesulfonate (51.7 µL, 0.28 mmol, 1.4 equiv) was added in one portion (addition was performed swiftly, while ensuring that no TMSOTf adhered to the wall of the Schlenk tube). After a clear solution had formed (ca. 5 min), the reaction mixture was cooled to –78 °C. After cooling, the silyl enol ether substrate (0.2 mmol, 1 equiv) was added as a solution in dichloromethane (2 x 1 M solution) and the resulting colourless solution was stirred at the same temperature for 30 min. After this time, triethylamine (112 µL, 0.8 mmol, 4 equiv) was added in one portion and the resulting solution was brought to ambient temperature after 5 min. Stirring was continued for 1 h, after which time the reaction mixture was transferred to a round-bottom flask using dichloromethane, followed by direct concentration using a rotary evaporator. The crude material was subsequently directly purified by flash column chromatography on silica gel to afford the desired products.

1,2-diphenylprop-2-en-1-one (7a)

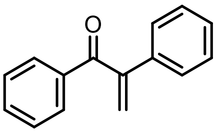

$C_{15}H_{12}O$
MW: 208.26
Yield: 82%
colourless oil

Synthesized following the general procedure, using *(Z)*-((1,3-diphenylprop-1-en-1-yl)oxy)trimethylsilane (**6a**, 56.5 mg, 0.20 mmol, 1 equiv). Purification by flash column chromatography over silica gel (heptane/toluene 50:50) gave the title compound (34.0 mg, 0.16 mmol, 82%). All analytical data were in good accordance with those reported in the literature.[12]

1-(2-methoxyphenyl)-2-phenylprop-2-en-1-one (7b)

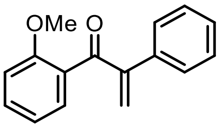

$C_{16}H_{14}O_2$
MW: 238.29
Yield: 75%
light yellow oil

Synthesized following the general procedure, using *(Z)*-((1-(2-methoxyphenyl)-3-phenylprop-1-en-1-yl)oxy)trimethylsilane (**6b**, 62.5 mg, 0.2 mmol, 1 equiv). Purification by flash column chromatography (toluene) gave the title compound (35.8 mg, 0.15 mmol, 75%).

^{1}H NMR (600 MHz, $CDCl_3$): δ 7.50 (dd, *J* = 7.5, 1.7 Hz, 1H), 7.44 – 7.39 (m, 3H), 7.35 – 7.30 (m, 3H), 7.01 (td, *J* = 7.5, 0.7 Hz, 1H), 6.89 (d, *J* = 8.3 Hz, 1H), 5.99 (s, 1H), 5.78 (s, 1H), 3.71 (s, 3H) ppm.

^{13}C NMR (151 MHz, $CDCl_3$): δ 197.6, 157.9, 150.5, 137.4, 132.6, 130.1, 129.3, 128.3 (2C), 128.13, 128.09 (2C), 124.9, 120.6, 111.6, 55.7 ppm.

IR (neat) ν_{max}: 2933, 1695, 1597, 1580, 1489, 1462, 1434, 1286, 1274, 1074, 753, 699 cm^{-1}.

HRMS (ESI^+) *m/z*: $[M+Na]^+$ calcd for $C_{16}H_{14}O_2Na$ 261.0886; found 261.0889.

1-(3-methoxyphenyl)-2-phenylprop-2-en-1-one (7c)

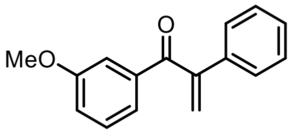

$C_{16}H_{14}O_2$
MW: 238.29
Yield: 80%
light yellow oil

Synthesized following the general procedure, using (*Z*)-((1-(3-methoxyphenyl)-3-phenylprop-1-en-1-yl)oxy)trimethylsilane (**6c**, 62.5 mg, 0.2 mmol, 1 equiv). Purification by flash column chromatography (heptane/toluene 50:50) gave the title compound (38 mg, 0.16 mmol, 80%).

^{1}H NMR (600 MHz, $CDCl_3$): δ 7.48 – 7.46 (m, 2H), 7.44 – 7.41 (m, 2H), 7.37 – 7.31 (m, 4H), 7.10 (d, *J* = 8.2 Hz, 1H), 6.06 (s, 1H), 5.65 (s, 1H), 3.83 (s, 3H) ppm.

^{13}C NMR (151 MHz, $CDCl_3$): δ 197.4, 157.8, 148.4, 138.5, 137.2, 129.5, 128.7 (2C), 128.5, 127.1 (2C), 123.1, 120.9, 119.9, 113.9, 55.5 ppm.

IR (neat) ν_{max}: 2931, 1663, 1595, 1579, 1483, 1462, 1447, 1427, 1322, 1284, 1252, 1216, 1195, 1042, 994, 777, 698, 683 cm^{-1}.

HRMS (ESI^+) *m/z*: $[M+H]^+$ calcd for $C_{16}H_{15}O_2$ 239.1067; found 239.1066.

1-(4-methoxyphenyl)-2-phenylprop-2-en-1-one (7d)

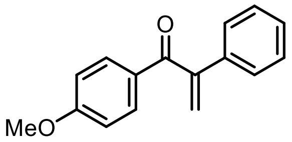

$C_{16}H_{14}O_2$
MW: 238.29
Yield: 59%
colourless oil

Synthesized following the general procedure, using *(Z)*-((1-(4-methoxyphenyl)-3-phenylprop-1-en-1-yl)oxy)trimethylsilane (**6d**, 62.5 mg, 0.2 mmol, 1 equiv). Purification by flash column chromatography (heptane/toluene 50:50) gave the title compound (28.0 mg, 0.12 mmol, 59%). All analytical data were in good accordance with those reported in the literature.[13]

1-(3-fluorophenyl)-2-phenylprop-2-en-1-one (7e)

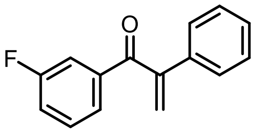

$C_{15}H_{11}FO$
MW: 226.25
Yield: 79%
colourless oil

Synthesized following the general procedure, using *(Z)*-((1-(3-fluorophenyl)-3-phenylprop-1-en-1-yl)oxy)trimethylsilane (**6e**, 60.1 mg, 0.2 mmol, 1 equiv). Purification by flash column chromatography (heptane/toluene 50:50) gave the title compound (35.5 mg, 0.16 mmol, 79%).

^{1}H NMR (500 MHz, $CDCl_3$): δ 7.67 (d, *J* = 7.7 Hz, 1H), 7.64 – 7.56 (m, 1H), 7.43 – 7.39 (m, 3H), 7.39 – 7.33 (m, 3H), 7.25 (td, *J* = 8.2, 2.3 Hz, 1H), 6.10 (s, 1H), 5.67 (s, 1H) ppm.

^{13}C NMR (126 MHz, $CDCl_3$): δ 196.3 (d, C–F, ^{4}J C–F = 2.2 Hz), 162.8 (d, C–F, ^{1}J C–F = 248.1 Hz), 148.1, 139.4 (d, C–F, ^{3}J C–F = 6.3 Hz), 136.8, 130.2 (d, C–F, ^{3}J C–F = 7.5 Hz), 128.8 (2C), 128.7, 127.2 (2C), 125.9 (d, C–F, ^{2}J C–F = 2.8 Hz), 121.7, 120.3 (d, C–F, ^{2}J C–F = 21.4 Hz), 116.7 (d, C–F, ^{2}J C–F = 22.7 Hz) ppm.

^{19}F NMR (471 MHz, $CDCl_3$): δ –111.9 ppm.

IR (neat) ν_{max}: 2917, 1690, 1611, 1585, 1268, 1131, 739, 699 cm^{-1}.

HRMS (ESI^{+}) *m/z*: $[M+H]^{+}$ calcd for $C_{15}H_{12}FO$ 227.0867; found 227.0870.

1-(3,5-dimethylphenyl)-2-phenylprop-2-en-1-one (7f)

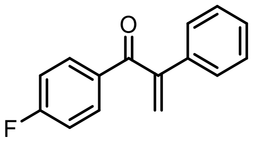

$C_{15}H_{11}OF$
MW: 226.25
Yield: 82%
light yellow oil

Synthesized following the general procedure, using (*Z*)-((1-(4-fluorophenyl)-3-phenylprop-1-en-1-yl)oxy)trimethylsilane (**6f**, 60.1 mg, 0.2 mmol, 1 equiv). Purification by flash column chromatography (heptane/toluene 50:50) gave the title compound (37 mg, 0.16 mmol, 82%). All analytical data were in good accordance with those reported in the literature.[14]

2-phenyl-1-(*o*-tolyl)prop-2-en-1-one (7g)

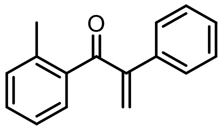

$C_{16}H_{14}O$
MW: 222.29
Yield: 85%
white solid

Synthesized following the general procedure, using (*Z*)-trimethyl((3-phenyl-1-(o-tolyl)prop-1-en-1-yl)oxy)silane (**6g**, 59.3 mg, 0.2 mmol, 1 equiv). Purification by flash column chromatography (heptane/toluene 50:50) gave the title compound (38 mg, 0.17 mmol, 85%). All analytical data were in good accordance with those reported in the literature.[15]

1-(4-(*tert*-butyl)phenyl)-2-phenylprop-2-en-1-one (7h)

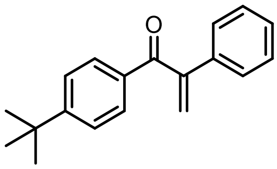

$C_{19}H_{20}O$
MW: 263.37
Yield: 58%
light yellow oil

Synthesized following the general procedure, using (*Z*)-((1-(4-(*tert*-butyl)phenyl)-3-phenylprop-1-en-1-yl)oxy)trimethylsilane (**6h**, 67.7 mg, 0.2 mmol, 1 equiv). Purification by flash column chromatography (heptane/toluene 50:50) gave the title compound (30 mg, 0.11 mmol, 58%).

^{1}H NMR (600 MHz, $CDCl_3$): δ 7.88 (d, *J* = 8.5 Hz, 2H), 7.48 – 7.43 (m, 4H), 7.37 – 7.31 (m, 3H), 6.04 (s, 1H), 5.61 (s, 1H), 1.34 (s, 9H) ppm.

^{13}C NMR (151 MHz, $CDCl_3$): δ 197.4, 157.1, 148.5, 137.3, 134.5, 130.2 (2C), 128.7 (2C), 128.5, 128.1, 127.1 (2C), 125.5 (2C), 120.0, 31.2 (3C) ppm.

IR (neat) ν_{max}: 2962, 2927, 1665, 1603, 1446, 1221, 1185, 981, 775, 700 cm^{-1}.

HRMS (ESI^+) *m/z*: $[M+Na]^+$ calcd for $C_{19}H_{20}ONa$ 287.1406; found 287.1406.

1-(3,5-dimethylphenyl)-2-phenylprop-2-en-1-one (7i)

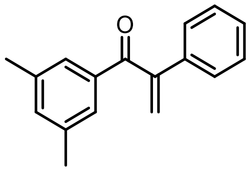

$C_{17}H_{16}O$
MW: 236.31
Yield: 82%
light yellow oil

Synthesized following the general procedure, using (*Z*)-((1-(3,5-dimethylphenyl)-3-phenylprop-1-en-1-yl)oxy)trimethylsilane (**6i**, 62.1 mg, 0.2 mmol, 1 equiv). Purification by flash column chromatography (heptane/toluene 50:50) gave the title compound (39 mg, 0.16 mmol, 82%).

¹H NMR (700 MHz, CDCl₃): δ 7.54 (s, 2H), 7.45 – 7.42 (m, 2H), 7.38 – 7.34 (m, 2H), 7.34 – 7.31 (m, 1H), 7.20 (s, 1H), 6.06 (s, 1H), 5.60 (s, 1H), 2.35 (s, 6H) ppm.

¹³C NMR (176 MHz, CDCl₃): δ 198.2, 148.4, 138.2, 137.4, 137.2, 135.0, 128.7 (2C), 128.5, 127.9 (2C), 127.1 (3C), 120.5, 21.3 (2C) ppm.

IR (neat) ν_{max}: 2919, 1663, 1597, 1494, 1444, 1324, 1308, 1173, 1161, 792, 779, 693, 605 cm^{-1}.

HRMS (ESI⁺) *m/z*: $[M+Na]^+$ calcd for $C_{17}H_{16}ONa$ 259.1093; found 259.1101.

2-phenyl-1-(4-(trifluoromethyl)phenyl)prop-2-en-1-one (7j)

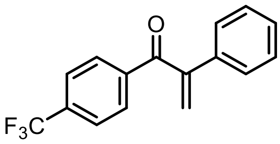

$C_{16}H_{11}F_3O$
MW: 276.26
Yield: 87%
colourless oil

Synthesized following the general procedure, using *(Z)*-trimethyl((3-phenyl-1-(4-(trifluoromethyl)phenyl)prop-1-en-1-yl)oxy)silane (**6j**, 70.1 mg, 0.2 mmol, 1 equiv). Purification by flash column chromatography (heptane/toluene 50:50) gave the title compound (48.1 mg, 0.17 mmol, 87%). All analytical data were in good accordance with those reported in the literature.[16]

1-(naphthalen-2-yl)-2-phenylprop-2-en-1-one (7k)

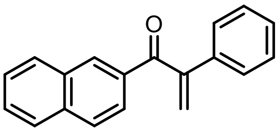

$C_{19}H_{14}O$
MW: 258.32
Yield: 74%
white solid

Synthesized following the general procedure, using (*Z*)-trimethyl((1-(naphthalen-2-yl)-3-phenylprop-1-en-1-yl)oxy)silane (**6k**, 66.5 mg, 0.2 mmol, 1 equiv). Purification by flash column chromatography (heptane/toluene 50:50) gave the title compound (38 mg, 0.15 mmol, 74%). All analytical data were in good accordance with those reported in the literature.[17]

1-(benzo[d][1,3]dioxol-5-yl)-2-phenylprop-2-en-1-one (7l)

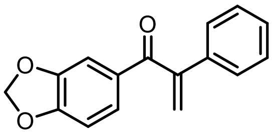

$C_{16}H_{12}O_3$
MW: 252.27
Yield: 71%
light yellow oil

Synthesized following the general procedure, using (*Z*)-((1-(benzo[d][1,3]dioxol-5-yl)-3-phenylprop-1-en-1-yl)oxy)trimethylsilane (**6l**, 65.3 mg, 0.2 mmol, 1 equiv). Purification by flash column chromatography (heptane/toluene 50:50) gave the title compound (36 mg, 0.14 mmol, 71%).

^{1}H NMR (600 MHz, CDCl$_3$): δ 7.43 (dd, *J* = 8.2, 1.7 Hz, 1H), 7.36 (d, *J* = 1.7 Hz, 1H), 7.34 – 7.31 (m, 2H), 7.27 – 7.21 (m, 3H), 6.72 (d, *J* = 8.2 Hz, 1H), 5.94 (s, 2H), 5,90 (s, 1H), 5.47 (s, 1H) ppm.

^{13}C NMR (151 MHz, CDCl$_3$): δ 196.0, 152.2, 148.4, 148.2, 137.1, 131.7, 128.8 (2C), 128.5, 127.2, 126.9 (2C), 119.1, 109.4, 107.9, 102.0 ppm.

IR (neat) v$_{max}$: 2904, 1657, 1602, 1502, 1486, 1440, 1357, 1247, 1037, 933, 701 cm^{-1}.

HRMS (ESI$^+$) *m/z*: [M+H]$^+$ calcd for $C_{16}H_{13}O_3$ 253.0859; found 253.0850.

2-phenyl-1-(thiophen-2-yl)prop-2-en-1-one (7m)

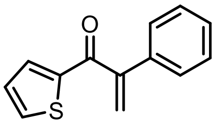

$C_{13}H_{10}OS$
MW: 214.28
Yield: 79%
light yellow oil

Synthesized following the general procedure, using *(Z)*-trimethyl((3-phenyl-1-(thiophen-2-yl)prop-1-en-1-yl)oxy)silane (**6m**, 57.7 mg, 0.2 mmol, 1 equiv). Purification by flash column chromatography (heptane/toluene 50:50) gave the title compound (34.2 mg, 0.16 mmol, 79%).

^{1}H NMR (600 MHz, CDCl$_3$): δ 7.68 (dd, *J* = 4.9, 1.1 Hz, 1H), 7.60 (dd, *J* = 3.8, 1.1 Hz, 1H), 7.48 – 7.44 (m, 2H), 7.38 – 7.33 (m, 3H), 7.09 (dd, *J* = 4.9, 3.8 Hz, 1H), 6.00 (s, 1H), 5.78 (s, 1H) ppm.

^{13}C NMR (151 MHz, CDCl$_3$): δ 189.5, 148.3, 144.3, 137.0, 135.2, 135.0, 128.8 (2C), 128.7, 128.2, 127.3 (2C), 120.3 ppm.

IR (neat) v$_{max}$: 1653, 1447, 1410, 1354, 1238, 1119, 1053, 725, 699 cm^{-1}.

HRMS (ESI$^+$) *m/z*: [M+Na]$^+$ calcd for $C_{13}H_{10}SONa$ 237.0345; found 237.0337.

3-phenylbut-3-en-2-one (7n)

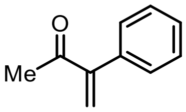

$C_{10}H_{10}O$
MW: 146.19
NMR Yield: 86%

Synthesized following the general procedure, using trimethyl((4-phenylbut-2-en-2-yl)oxy)silane (**6n**, 44.1 mg, 0.2 mmol, 1 equiv), reaction performed using dichloromethane-d2 (2 mL). Following completion of the reaction, mesitylene (27.8 µL, 0.2 mmol, 1 equiv) was added and the yield was determined to be 86% (0.17 mmol) by NMR using the internal standard. The observed signals were in good accordance with those reported in the literature.[18]

2-phenylacrylaldehyde (7o)

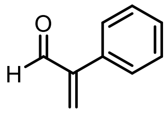

C_9H_8O
MW: 132.16
NMR Yield: 60%

Synthesized following the general procedure, using *(E)*-trimethyl((3-phenylprop-1-en-1-yl)oxy)silane (**6o**, 41.3 mg, 0.2 mmol, 1 equiv), reaction performed using dichloromethane-d2 (2 mL). Following completion of the reaction, mesitylene (27.8 µL, 0.2 mmol, 1 equiv) was added and the yield was determined to be 60% (0.12 mmol) by NMR using the internal standard. The observed signals were in good accordance with those reported in the literature.[18]

1-phenyl-2-(*o*-tolyl)prop-2-en-1-one (7p)

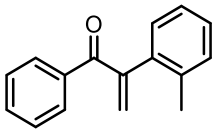

$C_{16}H_{14}O$
MW: 222.29
Yield: 57%
white solid

Synthesized following the general procedure, using (*Z*)-trimethyl((1-phenyl-3-(o-tolyl)prop-1-en-1-yl)oxy)silane (**6p**, 59.3 mg, 0.2 mmol, 1 equiv). Purification by flash column chromatography (heptane/ethyl acetate 98:2) gave the title compound (25 mg, 0.11 mmol, 57%).

^{1}H NMR (700 MHz, CDCl$_3$): δ 7.90 (d, *J* = 8.1 Hz, 2H), 7.55 (t, *J* = 7.4 Hz, 1H), 7.45 (t, *J* = 7.8 Hz, 2H), 7.30 – 7.23 (m, 3H), 7.18 (d, *J* = 7.2 Hz, 1H), 6.03 (s, 1H), 6.00 (s, 1H), 2.22 (s, 3H) ppm.

^{13}C NMR (176 MHz, CDCl$_3$): δ 196.6, 149.3, 138.4, 137.4, 135.7, 132.7, 130.4, 129.9 (3C), 128.5, 128.4 (2C), 128.1, 126.2, 20.6 ppm.

IR (neat) ν_{max}: 2923, 1656, 1619, 1447, 1329, 1276, 1218, 979, 741, 729, 701 cm^{-1}.

MP: 73 – 76 °C.

HRMS (ESI$^+$) *m/z*: $[M+H]^+$ calcd for $C_{16}H_{15}O$ 223.1117; found 223.1119.

2-(3-fluorophenyl)-1-phenylprop-2-en-1-one (7q)

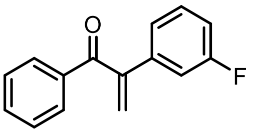

$C_{15}H_{11}OF$
MW: 226.08
Yield: 51%
light yellow oil

Synthesized following the general procedure, using (*Z*)-((3-(3-fluorophenyl)-1-phenylprop-1-en-1-yl)oxy)trimethylsilane (**6q**, 60.1 mg, 0.2 mmol, 1 equiv). Purification by flash column chromatography (heptane/toluene 50:50) gave the title compound (23 mg, 0.10 mmol, 51%).

^{1}H NMR (600 MHz, CDCl$_3$): δ 7.91 – 7.89 (m, 2H), 7.59 – 7.56 (m, 1H), 7.47 – 7.43 (m, 2H), 7.33 – 7.30 (m, 1H), 7.21 – 7.16 (m, 2H), 7.03 (td, *J* = 8.5, 0.8 Hz, 1H), 6.10 (s, 1H), 5.70 (s, 1H) ppm.

^{13}C NMR (151 MHz, CDCl$_3$): δ 197.1, 163.0 (d, *J* = 246 Hz), 147.3 (d, *J* = 1.7 Hz), 139.2 (d, *J* = 7.9 Hz), 137.0, 133.4, 130.3 (d, *J* = 8.6 Hz), 130.1 (2C), 128.6 (2C), 123.0 (d, *J* = 2.6 Hz), 122.3, 115.5 (d, *J* = 21.0 Hz), 114.2 (d, *J* = 22.4 Hz) ppm.

^{19}F NMR (565 MHz, CDCl$_3$): δ –112.7 ppm.

IR (neat) ν_{max}: 3069, 2949, 2870, 1725, 1684, 1589, 1486, 1447, 1264, 1235, 787, 692 cm^{-1}.

HRMS (ESI$^+$) *m/z*: [M+Na]$^+$ calcd for $C_{15}H_{11}OFNa$ 249.0686; found 249.0693.

2-(3,4-dimethoxyphenyl)-1-phenylprop-2-en-1-one (7r)

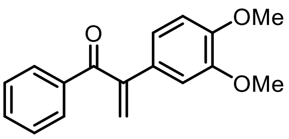

$C_{17}H_{16}O_3$
MW: 268.31
Yield: 65%
light yellow oil

Synthesized following the general procedure, using (*Z*)-((3-(3,4-dimethoxyphenyl)-1-phenylprop-1-en-1-yl)oxy)trimethylsilane (**6r**, 68.5 mg, 0.2 mmol, 1 equiv). Purification by flash column chromatography (heptane/ethyl acetate 98:2) gave the title compound (35 mg, 0.13 mmol, 65%).

^{1}H NMR (600 MHz, CDCl$_3$): δ 7.91 (d, *J* = 7.4 Hz, 2H), 7.54 (t, *J* = 7.3 Hz, 1H), 7.43 (t, *J* = 7.7 Hz, 2H), 6.98 – 6.95 (m, 2H), 6.82 (d, *J* = 8.3 Hz, 1H), 5.98 (s, 1H), 5.54 (s, 1H), 3.87 (s, 3H), 3.85 (s, 3H) ppm.

^{13}C NMR (151 MHz, CDCl$_3$): δ 197.9, 149.5, 149.0, 147.9, 137.3, 133.2, 130.1 (2C), 129.9, 128.5 (2C), 120.1, 119.4, 111.2, 110.1, 56.0 (2C) ppm.

IR (neat) ν_{max}: 2933, 1667, 1514, 1257, 1238, 1144, 1026, 728 cm^{-1}.

HRMS (ESI$^+$) *m/z*: [M+Na]$^+$ calcd for $C_{17}H_{16}O_3Na$ 291.0992; found 291.0994.

(3,4-dihydronaphthalen-1-yl)(phenyl)methanone (7t)

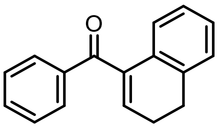

Synthesized following the general procedure, using *(Z)*-((2-(2,3-dihydro-1H-inden-1-yl)-1-phenylvinyl)oxy)trimethylsilane (**6t**, 62.3 mg, 0.2 mmol, 1 equiv). Purification by flash column chromatography (heptane/ethyl acetate 10:1) gave the title compound (41.2 mg, 0.17 mmol, 88%). All analytical data were in good accordance with those reported in the literature.[19]

phenanthren-9-yl(phenyl)methanone (7u)

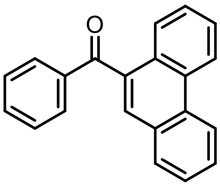

Synthesized following the general procedure, using (*Z*)-((2-(9H-fluoren-9-yl)-1-phenylvinyl)oxy)trimethylsilane (**6u**, 71.3 mg, 0.2 mmol, 1 equiv). Purification by flash column chromatography (heptane/ethyl acetate 95:5) gave the title compound (47 mg, 0.17 mmol, 83%).

^{1}H NMR (600 MHz, CDCl$_3$): δ 8.35 (d, *J* = 7.8 Hz, 1H), 8.13 – 8.11 (m, 2H), 7.78 (d, *J* = 7.6 Hz, 1H), 7.65 (dd, *J* = 7.6, 3.1 Hz, 2H), 7.63 – 7.60 (m, 2H), 7.54 – 7.51 (m, 2H), 7.43 – 7.37 (m, 2H), 7.31 (td, *J* = 7.5, 1.0 Hz, 1H), 7.23 (td, *J* = 7.6, 1.0 Hz, 1H) ppm.

^{13}C NMR (151 MHz, CDCl$_3$): δ 192.7, 146.3, 142.4, 141.0, 138.9, 138.4, 135.5, 133.5, 130.9, 130.6, 128.94 (2C), 128.9 (2C), 128.1, 127.7, 127.6, 121.2, 120.0, 119.8, 119.5 ppm.

IR (neat) v_{max}: 3058, 1657, 1595, 1585, 1449, 1234, 1014, 782, 743, 728, 695 cm^{-1}.

MP: 130 – 133 °C.

HRMS (ESI$^+$) *m/z*: [M+Na]$^+$ calcd for $C_{21}H_{14}ONa$ 305.0937; found 305.0933.

(*E*)-1,2-diphenylbut-2-en-1-one (7v)

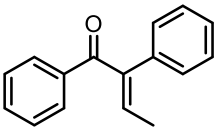

$C_{16}H_{14}O$
MW: 222.29
Yield: 90%, *E*/*Z* = 96:4
colourless oil

Synthesized following the general procedure, using (*Z*)-((1,3-diphenylbut-1-en-1-yl)oxy)trimethylsilane (**6v**, 59.3 mg, 0.2 mmol, 1 equiv). Purification by flash column chromatography (heptane/ethyl acetate 95:5) gave the title compound (40 mg, 0.18 mmol, 90%). All analytical data were in good accordance with those reported in the literature.[13]

^{1}H NMR (700 MHz, CDCl$_3$): *E*/*Z* 96:4 (data for major isomer) δ 7.78 (d, *J* = 7.5 Hz, 2H), 7.52 – 7.49 (m, 1H), 7.43 – 7.39 (m, 4H), 7.34 – 7.30 (m, 1H), 7.29 – 7.27 (m, 2H), 6.60 (q, *J* = 7.1 Hz, 1H), 1.89 (d, *J* = 7.1 Hz, 3H) ppm.

^{13}C NMR (176 MHz, CDCl$_3$): *E*/*Z* 96:4 (data for major isomer) δ 197.3, 143.0, 139.8, 138.5, 135.9, 132.0, 129.7 (4C), 128.3 (2C), 128.2 (2C), 127.6, 15.7 ppm.

(*E*)-1,2,5-triphenylpent-2-en-1-one (7w)

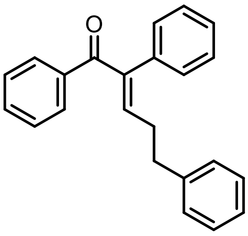

$C_{23}H_{20}O$
MW: 312.41
Yield: 86%
light yellow oil

Synthesized following the general procedure, using (*Z*)-trimethyl((1,3,5-triphenylpent-1-en-1-yl)oxy)silane (**6w**, 77.3 mg, 0.2 mmol, 1 equiv). Purification by flash column chromatography (heptane/ethyl acetate 95:5) gave the title compound (54 mg, 0.17 mmol, 86%).

^{1}H NMR (600 MHz, CDCl$_3$): δ 7.59 – 7.58 (m, 2H), 7.39 (t, *J* = 7.4 Hz, 1H), 7.30 – 7.24 (m, 4H), 7.21 – 7.17 (m, 3H), 7.13 – 7.10 (m, 1H), 7.05 – 7.04 (m, 2H), 7.01 – 7.01 (m, 2H), 6.33 (t, *J* = 7.4 Hz, 1H), 2.67 (t, *J* = 7.5 Hz, 2H), 2.49 (q, *J* = 7.5 Hz, 2H) ppm.

^{13}C NMR (151 MHz, CDCl$_3$): δ 197.3, 143.3, 142.4, 140.9, 138.3, 136.0, 132.1, 129.8 (2C), 129.5 (2C), 128.6 (2C), 128.5 (2C), 128.3 (2C), 128.2 (2C), 127.6, 126.3, 25.2, 31.3 ppm.

IR (neat) ν_{max}: 2923, 1650, 1596, 1577, 1494, 1444, 1266, 757, 697 cm^{-1}.

HRMS (ESI$^+$) *m/z*: [M+Na]$^+$ calcd for $C_{23}H_{20}ONa$ 335.1406; found 335.1409.

2,2-dimethyl-4,5-diphenyl-2,3-dihydrofuran (7x)

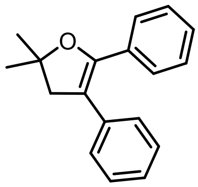

$C_{18}H_{18}O$
MW: 250.13
Yield: 62%
white solid

Synthesized following the general procedure, using (*Z*)-trimethyl((4-methyl-1,3-diphenylpent-1-en-1-yl)oxy)silane (**6x**, 64.9 mg, 0.2 mmol, 1 equiv). Purification by flash column chromatography (heptane/ethyl acetate 95:5) gave the title compound (31 mg, 0.12 mmol, 62%). All analytical data were in good accordance with those reported in the literature.[20]

2.4. General procedure for 5 mmol scale of 7a

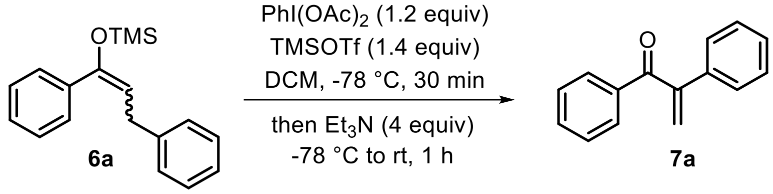

Iodosobenzene diacetate (1.99 g, 6 mmol, 1.2 equiv) was placed in a flame-dried Schlenk tube and dichloromethane (0.1 M with respect to the silyl enol ether) was added. The suspension was cooled to 0 °C, after which trimethylsilyl trifluoromethanesulfonate (1.29 mL, 7 mmol, 1.4 equiv) was added in one portion (addition was performed swiftly, while ensuring that no TMSOTf adhered to the wall of the Schlenk tube). After a clear solution had formed (ca. 5 min), the mixture was cooled to –78 °C. After cooling, **6a** (5 mmol, 1 equiv) was added as a solution in dichloromethane (2 x 1 M solution) and the resulting colourless solution was stirred at the same temperature for 30 min. After this time, triethylamine (2.79 mL, 20 mmol, 4 equiv) was added in one portion and the resulting solution was brought to ambient temperature after 5 min. Stirring was continued for 1 h, after which time the reaction mixture was transferred to a round-bottom flask using dichloromethane, followed by direct concentration using a rotary evaporator. The crude material was subsequently directly purified by flash column chromatography on silica gel (heptane/toluene 50:50) to afford **7a** in 75% yield (785 mg, 3.77 mmol).

2.5. Nazarov reaction

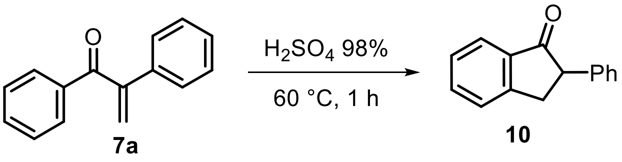

A solution of **7a** (41.7 mg, 0.2 mmol, 1 equiv) in 0.5 mL of sulfuric acid (H_2SO_4, 98%) was heated (oil bath) at 60 °C for 1 h. After allowing the system reach ambient temperature, the mixture was diluted by addition of ethyl acetate (1 mL), followed by the careful addition of a saturated aqueous solution of sodium bicarbonate (1 mL). The layers were separated and the aqueous layer was extracted with ethyl acetate (2 x 1 mL). The combined organic layers were dried over anhydrous magnesium sulfate, filtered and the solvent was evaporated under reduced pressure. The crude mixture was purified by flash column chromatography on silica gel (toluene) to give the title compound in 59% yield (24 mg, 0.12 mmol). All analytical data were in good accordance with those reported in the literature.[21]

2.6. (3+2)-Cycloaddition reaction

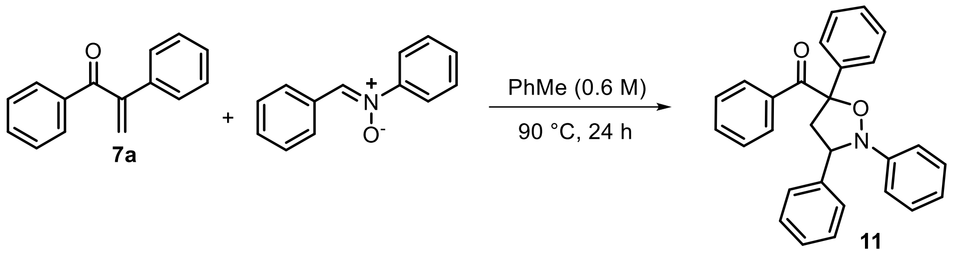

A solution of **7a** (25 mg, 0.12 mmol, 1.1 equiv) and *N*-phenyl-*C*-phenyl nitrone[22] (21.5 mg, 0.11 mmol, 1 equiv) in anhydrous toluene (0.18 mL, 0.6 M) was heated (oil bath) at 90 °C for 24 h. After this time, the reaction mixture was allowed to cool to ambient temperature, after which it was concentrated under reduced pressure. The resulting crude material was purified by flash column chromatography on silica gel (toluene/heptane 1:1) to give the title compound in 97% yield (43 mg, 0.106 mmol).

phenyl(2,3,5-triphenylisoxazolidin-5-yl)methanone (11)

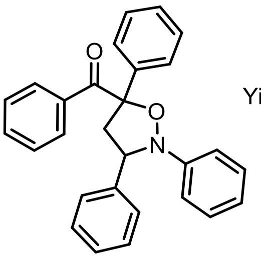

$C_{28}H_{23}NO_2$
MW: 405.50
Yield: 97%, *dr* = 3:1
yellowish oil

^{1}H NMR (700 MHz, $CDCl_3$): Mixture of diastereoisomers 3:1. δ 8.11– 8.10 (m, 0.6H, minor isomer), 8.00 – 7.98 (m, 2H, major isomer), 7.65 – 7.63 (m, 2H, major isomer), 7.60 – 7.58 (m, 0.6H, minor isomer), 7.50 – 7.47 (m, 0.3H, minor isomer), 7.47 – 7.44 (m, 1H, major isomer), 7.42 – 7.37 (m, 6H, for two isomers), 7.36 – 7.28 (m, 7H, for two isomers), 7.27 – 7.24 (m, 1H, minor isomer), 7.17 – 7.13 (m, 0.6H, minor isomer), 7.03 – 6.99 (m, 2H, major isomer), 6.98 – 6.95 (m, 0.3H, minor isomer), 6.91 – 6.88 (m, 0.6H, minor isomer), 6.84 – 6.81 (m, 1H, major isomer), 6.81 – 6.78 (m, 2H, major isomer), 4.66 (t, *J* = 8.1 Hz, 1H, major isomer), 4.55 (dd, *J* = 9.6, 6.7 Hz, 0.3 H, minor isomer), 4.18 (dd, *J* = 12.7, 7.5 Hz, 1H, major isomer), 3.84 (dd, *J* = 12.6, 6.7 Hz, 0.3 H, minor isomer), 2.88 (dd, *J* = 12.6, 6.7 Hz, 0.3 H, minor isomer), 2.63 (dd, *J* = 12.7, 8.9 Hz, 1H, major isomer) ppm.

^{13}C NMR (176 MHz, $CDCl_3$): Mixture of diastereosiomers 3:1. δ 200.5, 197.4, 150.0, 148.9, 140.9, 140.7, 140.5, 139.3, 135.2, 134.7, 132.9, 132.6, 130.8 (2C), 130.3, 129.1 (2C), 129.0, 128.9 (2C), 128.6, 128.4 (2C), 128.1 (2C), 128.11, 128.08, 128.0, 127.82, 127.8, 127.2 (2C), 127.16, 124.9, 124.6 (2C), 123.2, 122.3, 117.7, 116.2 (2C), 91.4, 90.3, 70.0, 68.7, 51.9, 51.0 ppm.

IR (neat) ν_{max}: 3087, 3061, 2954, 2926, 2869, 2855, 1681, 1597, 1489, 1448, 1260, 1181, 753, 698 cm^{-1}.

HRMS (ESI$^+$) *m/z*: $[M+Na]^+$ calcd for $C_{28}H_{23}ONNa$ 428.1621; found 428.1621.

3. Rationalization of stereochemical outcome (7v, 7w)

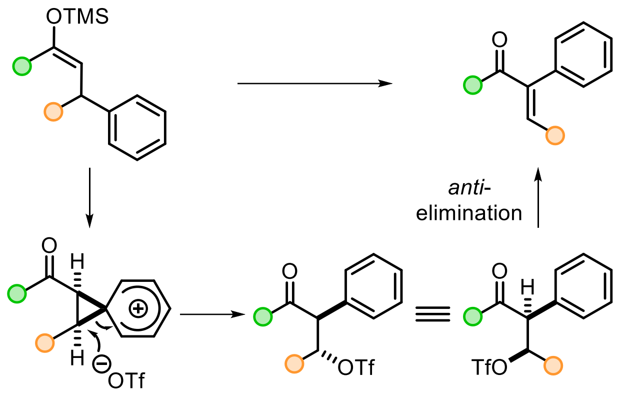

4. NMR Spectra

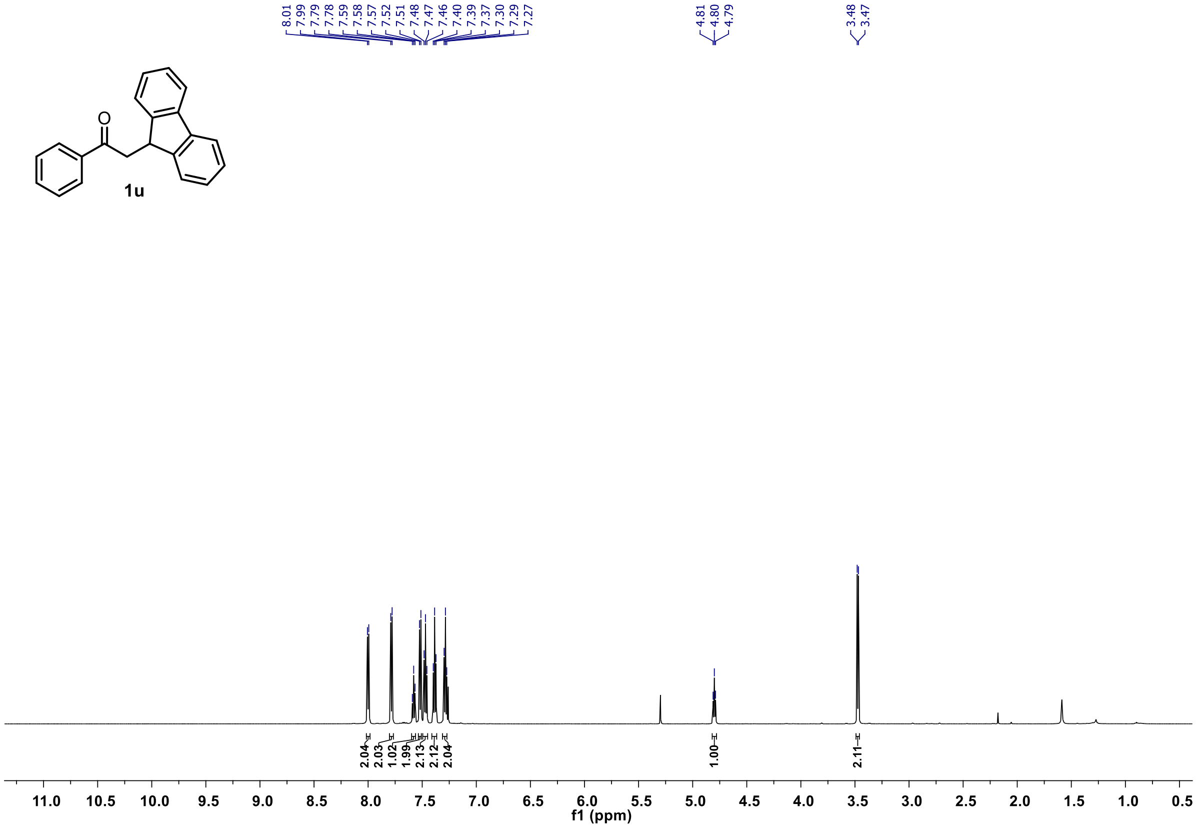

^{1}H NMR in $CDCl_3$ at 600 MHz

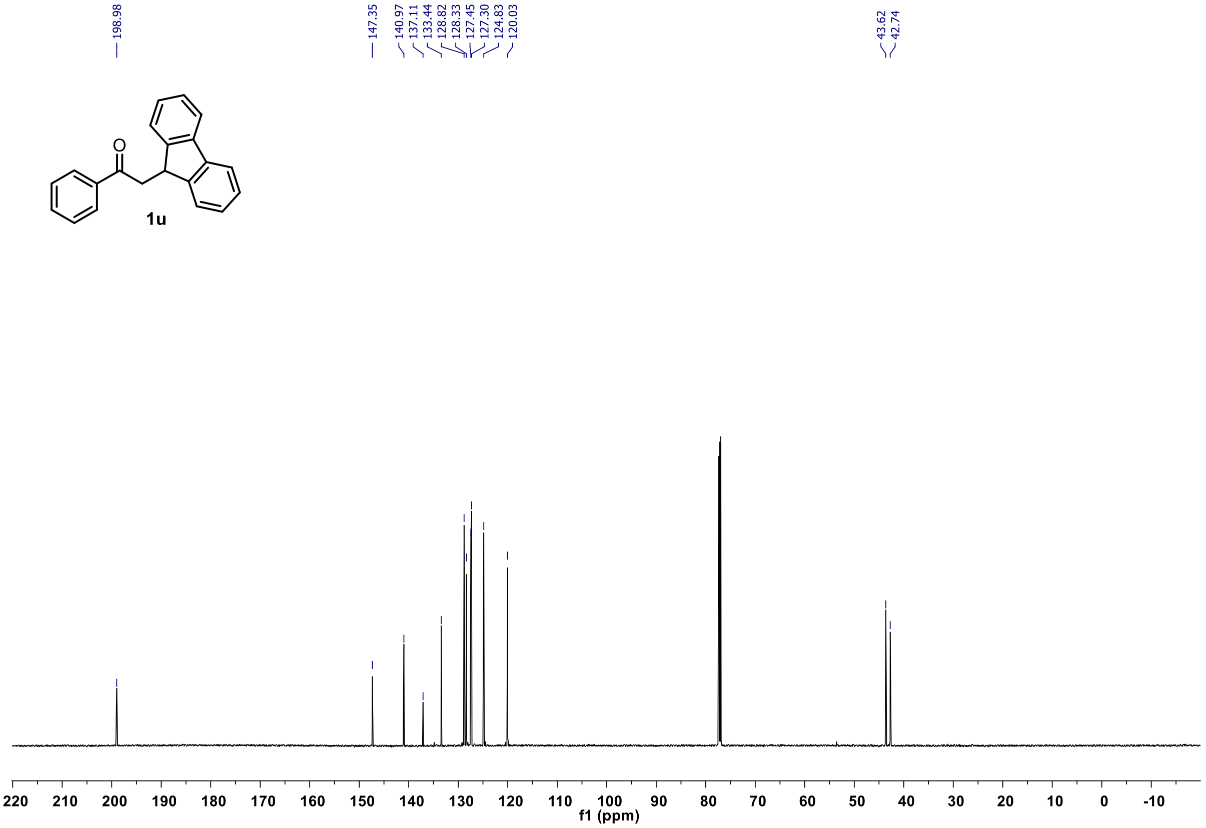

^{13}C NMR in $CDCl_3$ at 151 MHz

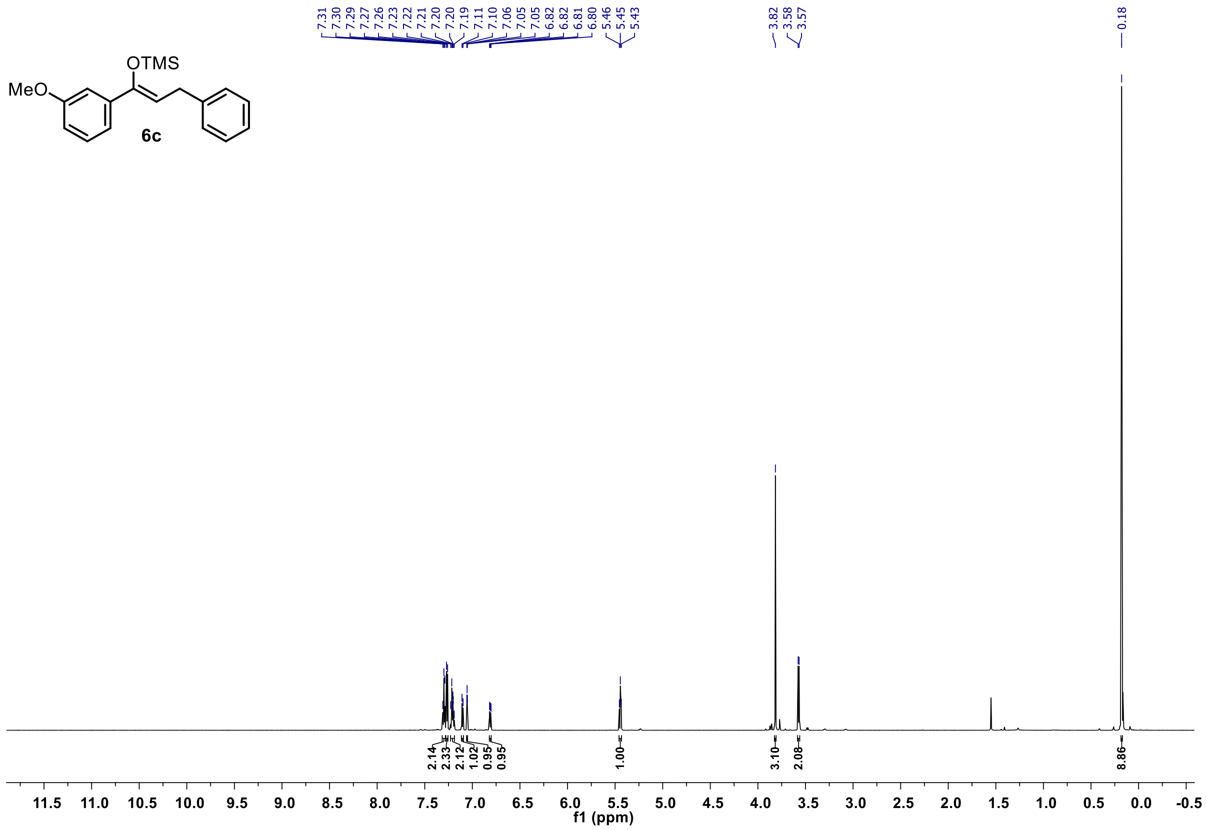

^{1}H NMR in $CDCl_3$ at 700 MHz

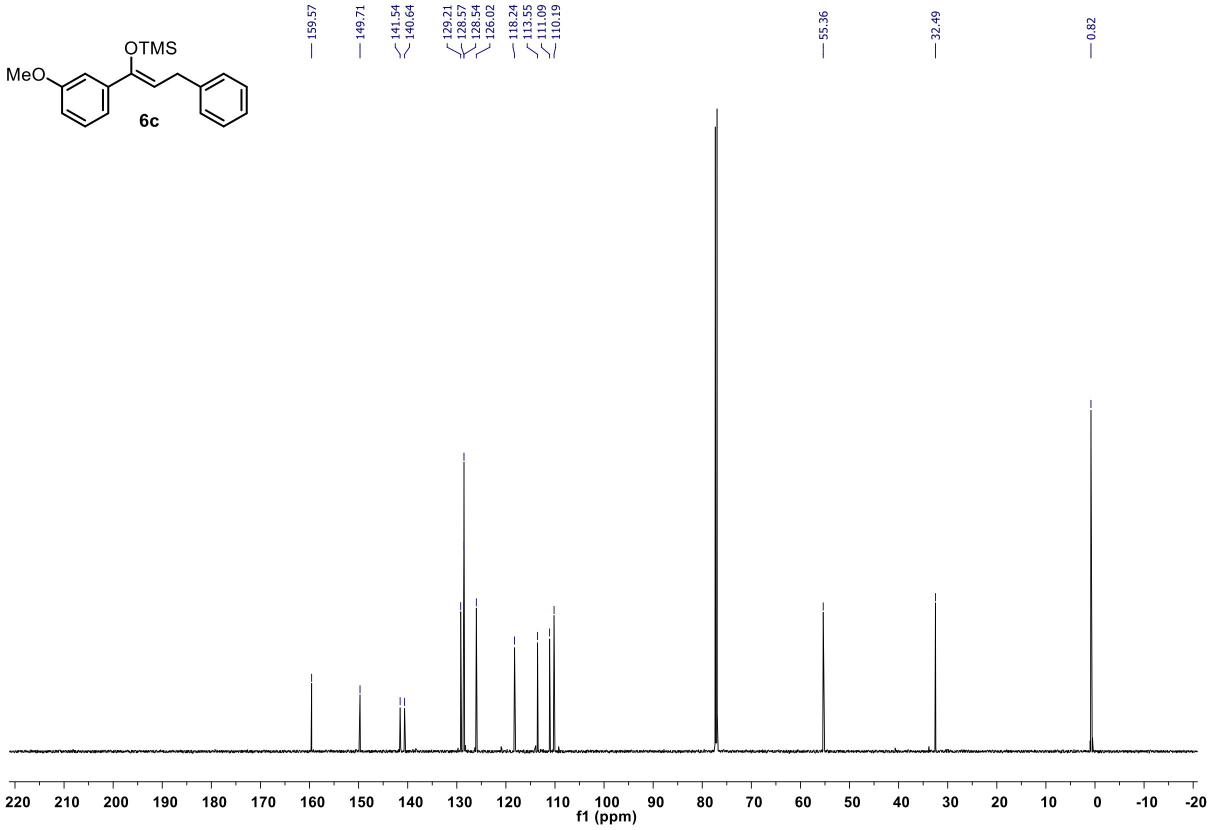

^{13}C NMR in $CDCl_3$ at 176 MHz

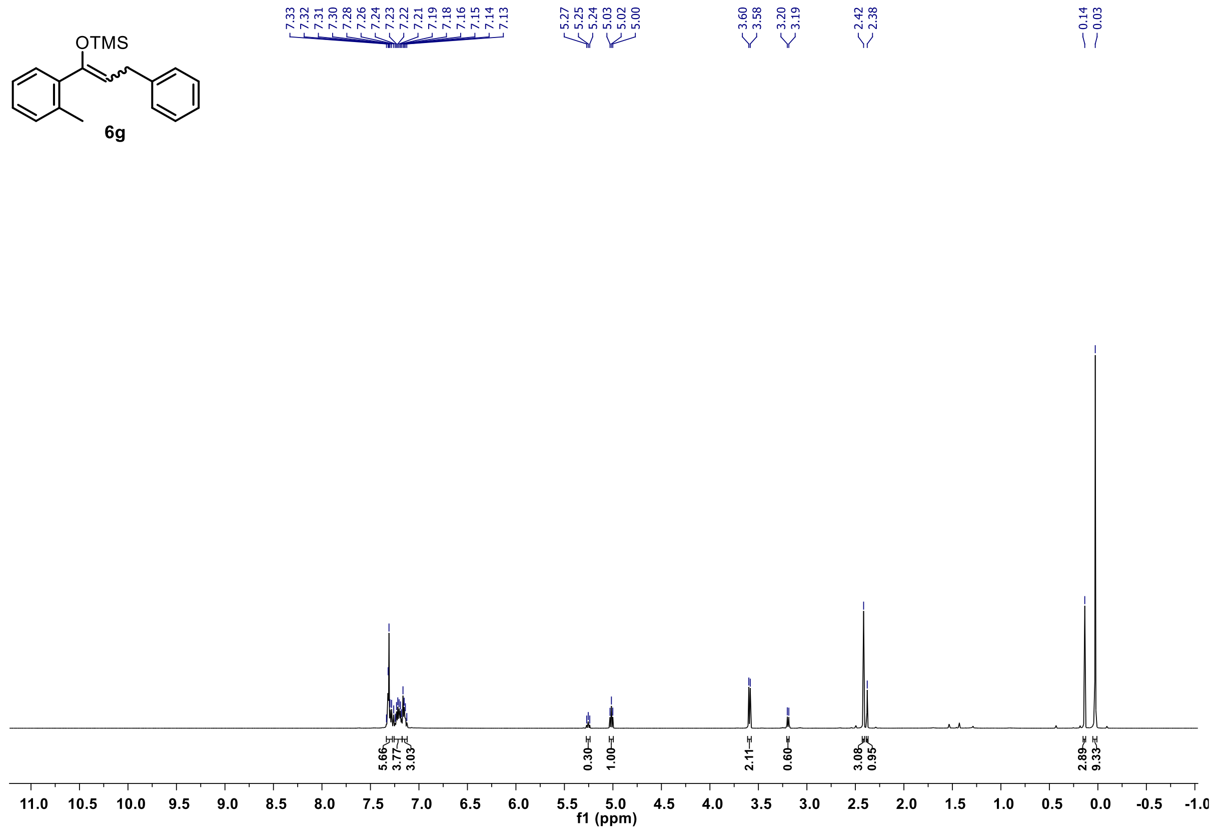

^{1}H NMR in $CDCl_3$ at 500 MHz

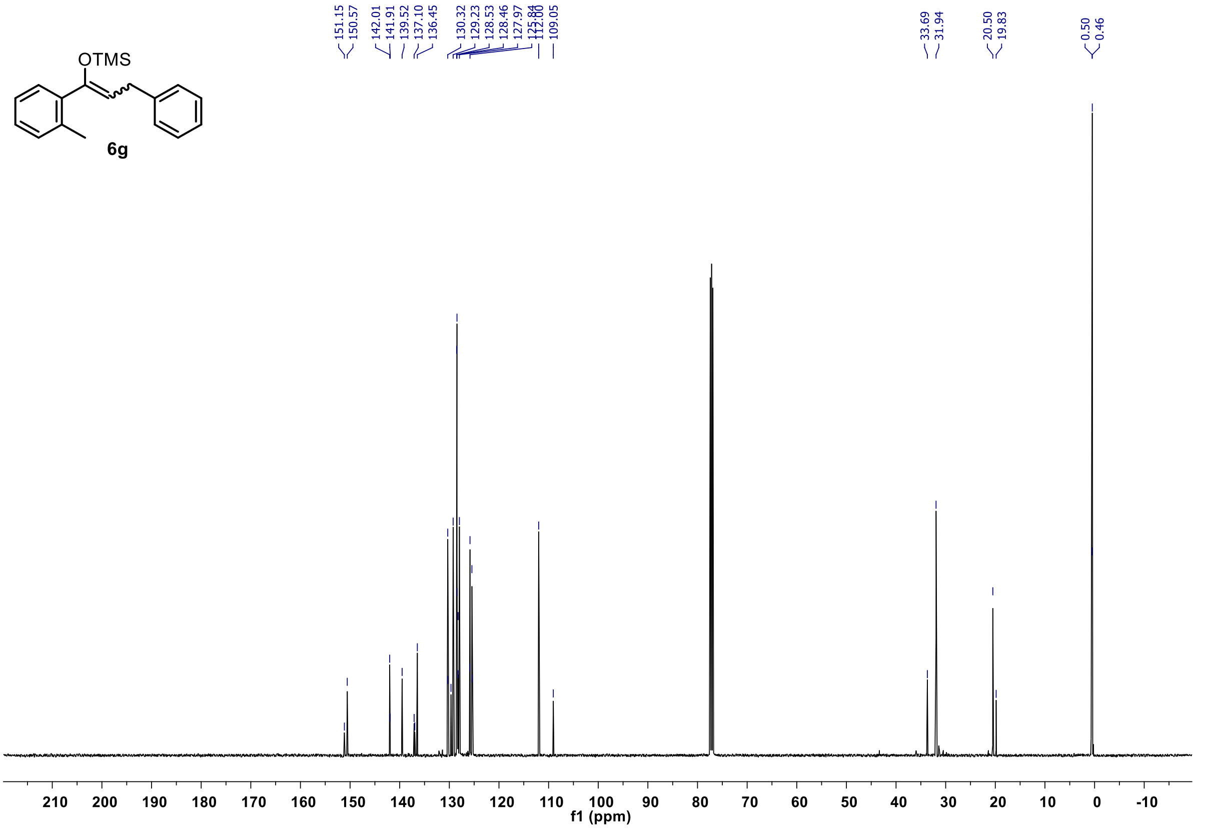

^{13}C NMR in $CDCl_3$ at 126 MHz

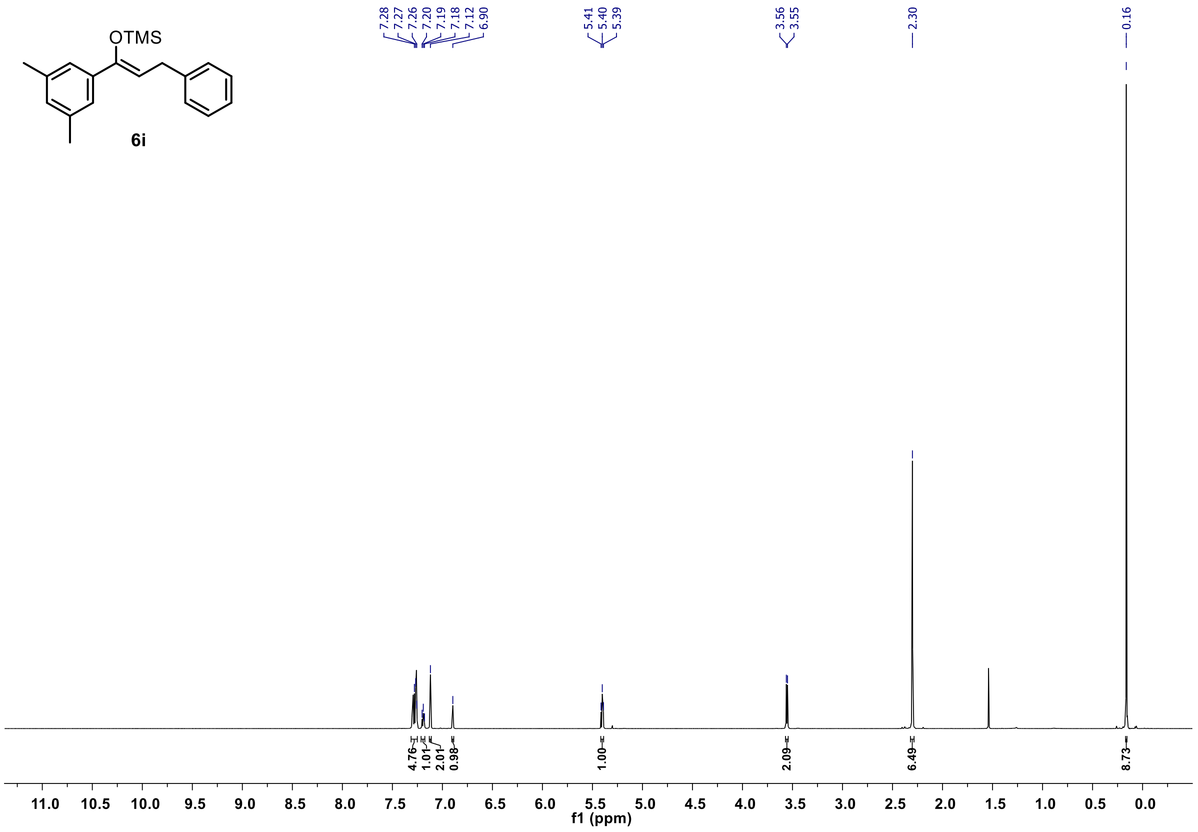

^{1}H NMR in $CDCl_3$ at 600 MHz

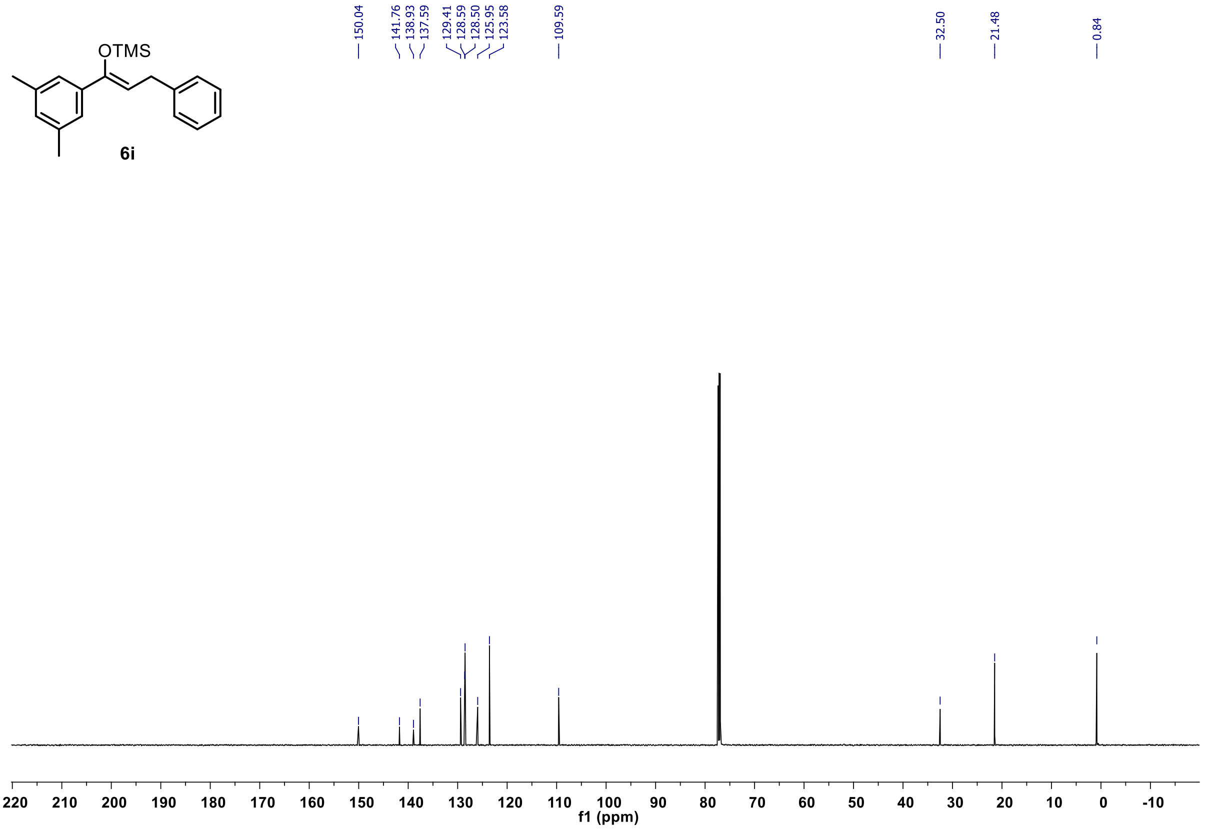

^{13}C NMR in $CDCl_3$ at 151 MHz

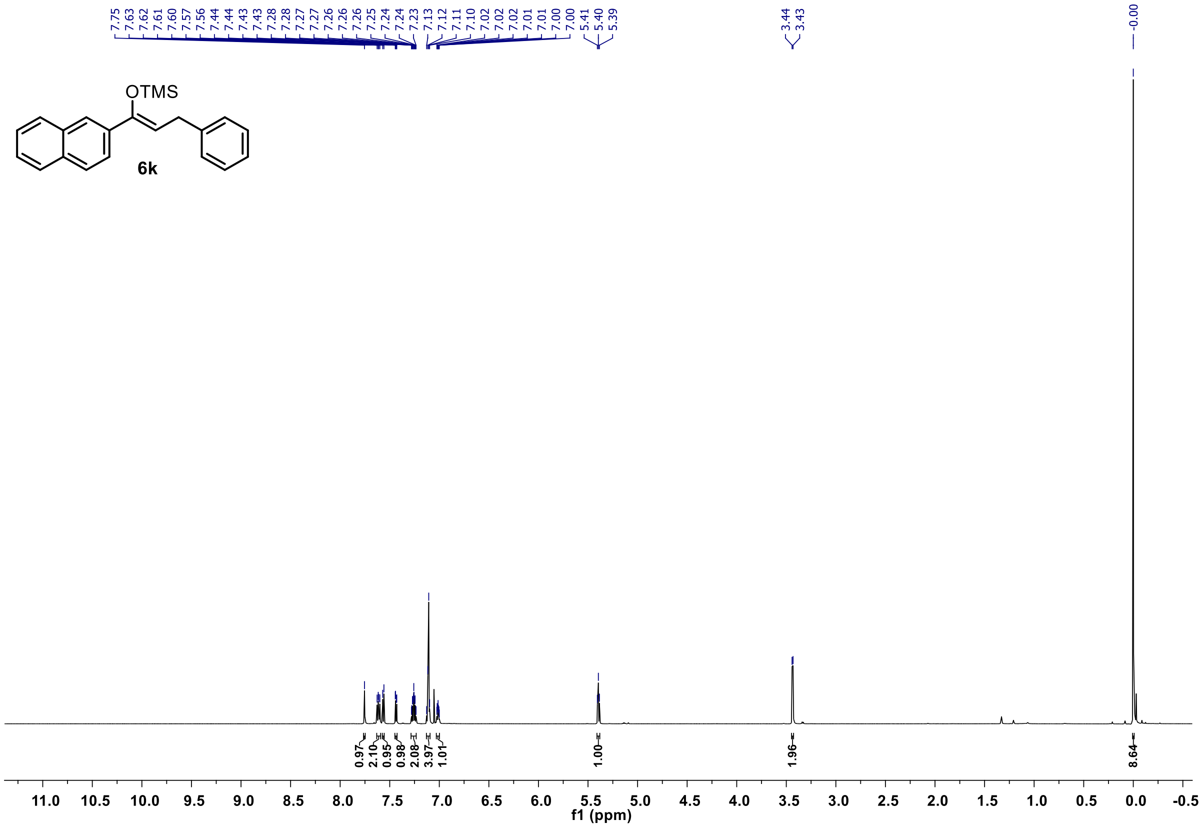

^{1}H NMR in $CDCl_3$ at 700 MHz

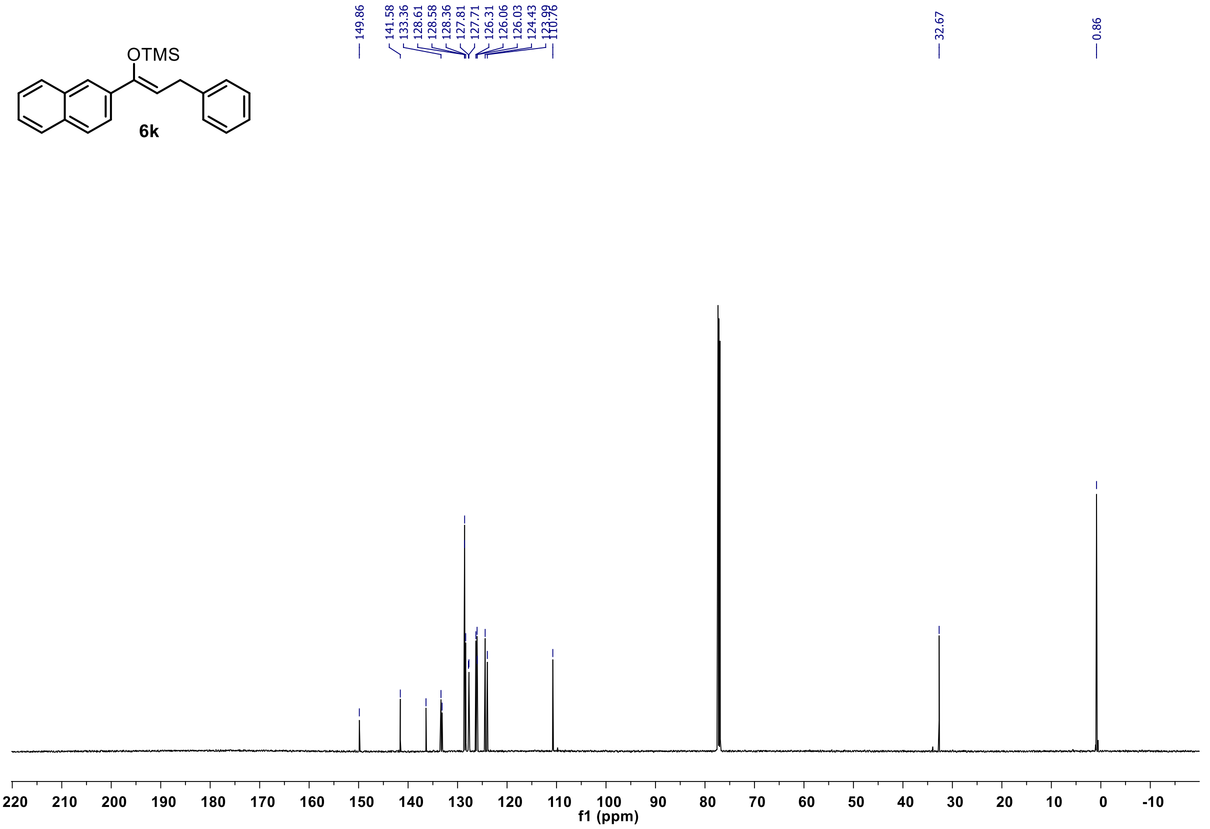

^{13}C NMR in $CDCl_3$ at 151 MHz

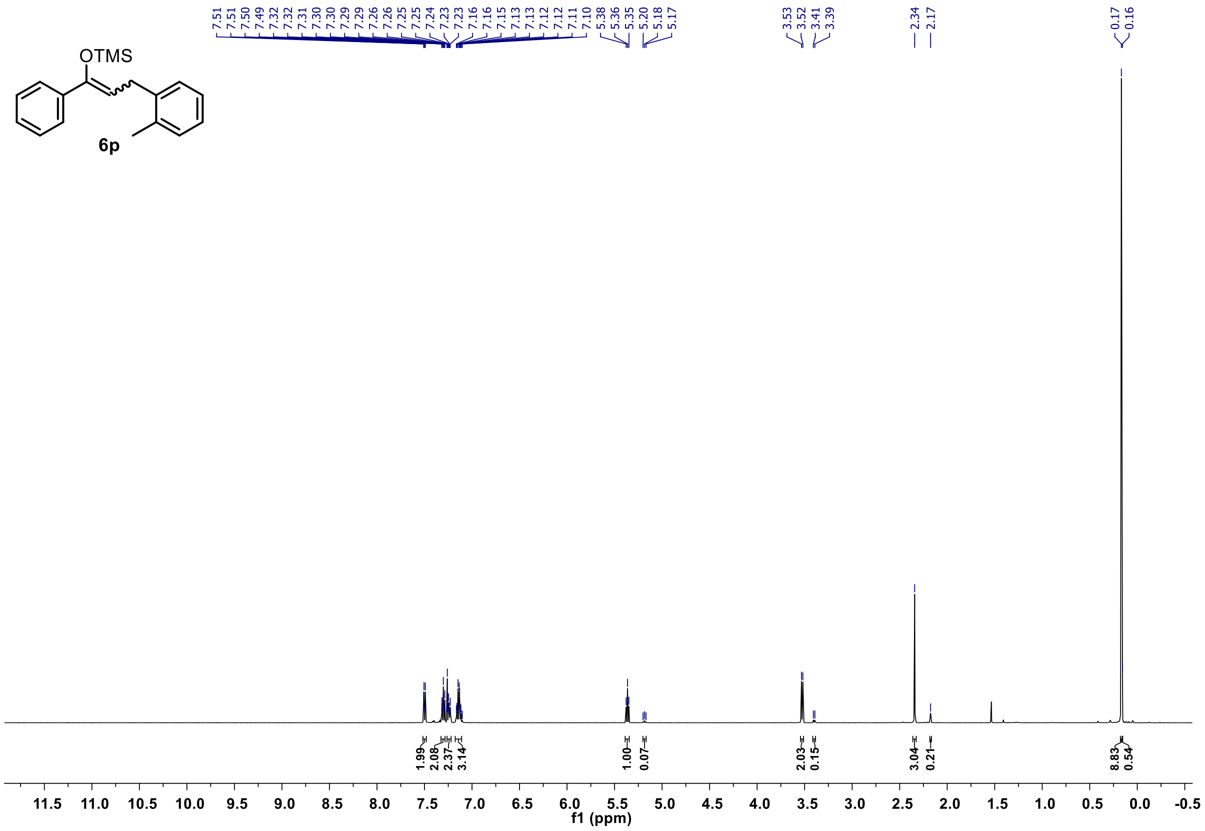

^{1}H NMR in $CDCl_3$ at 500 MHz

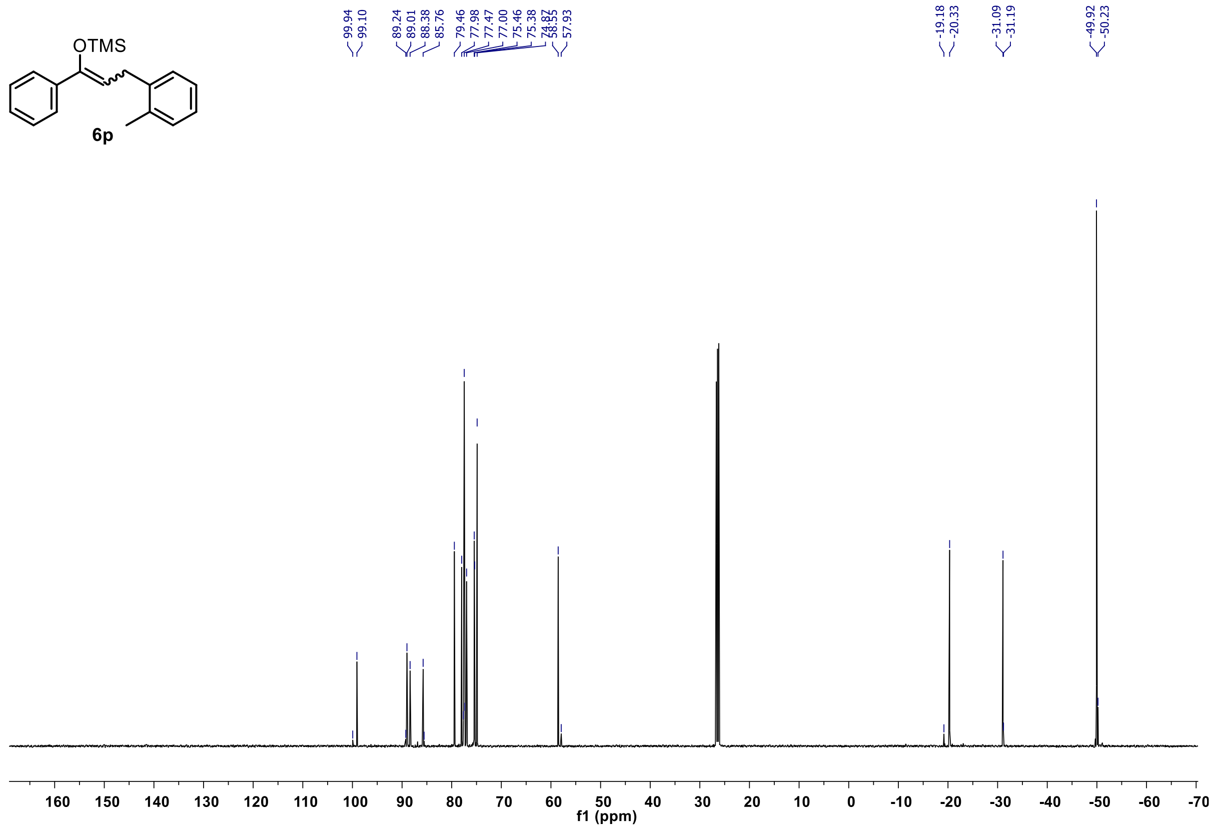

^{13}C NMR in $CDCl_3$ at 126 MHz

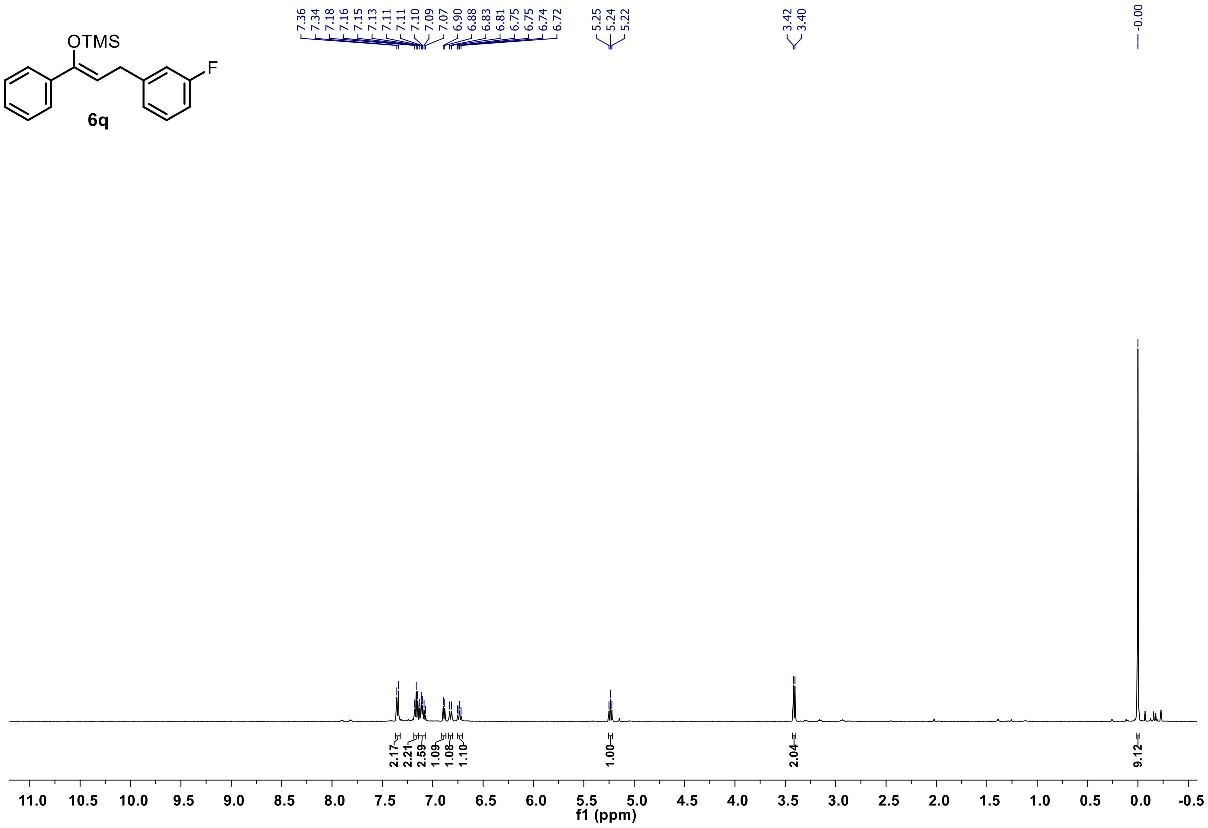

^{1}H NMR in $CDCl_3$ at 500 MHz

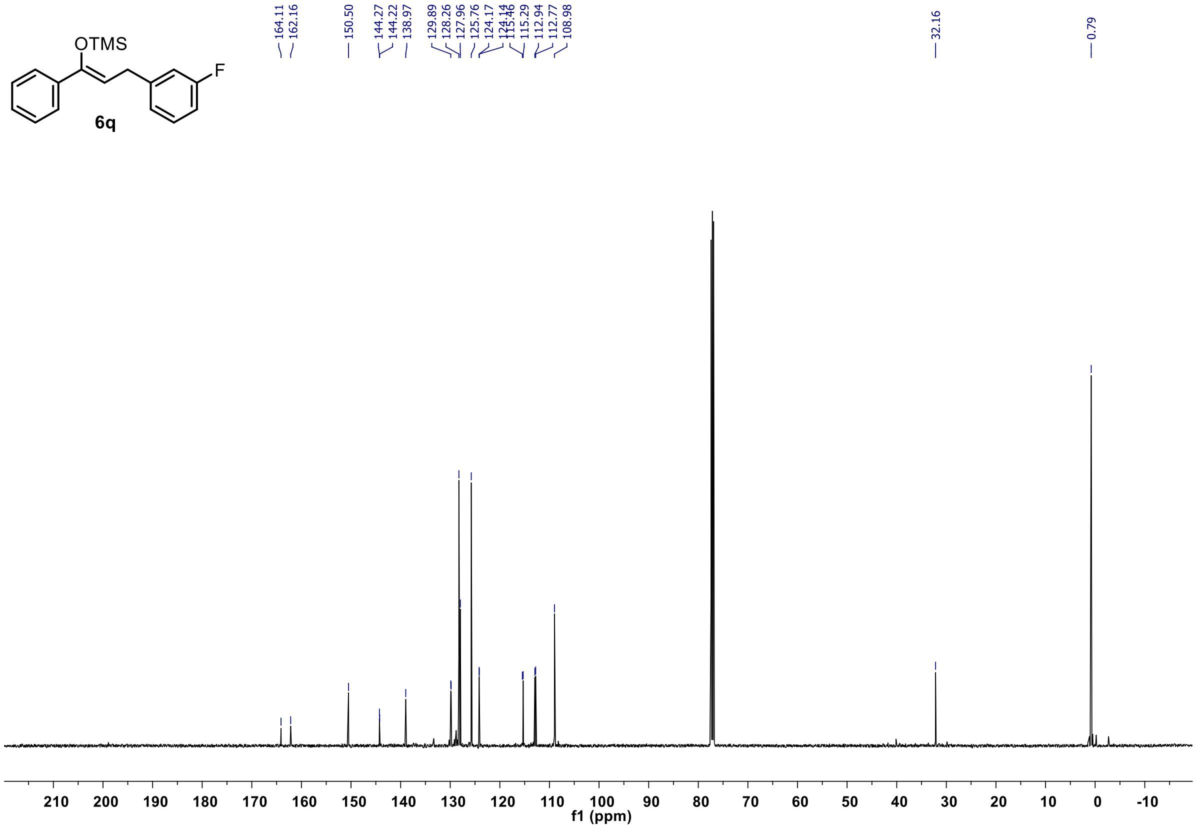

^{13}C NMR in $CDCl_3$ at 126 MHz

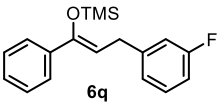

6q

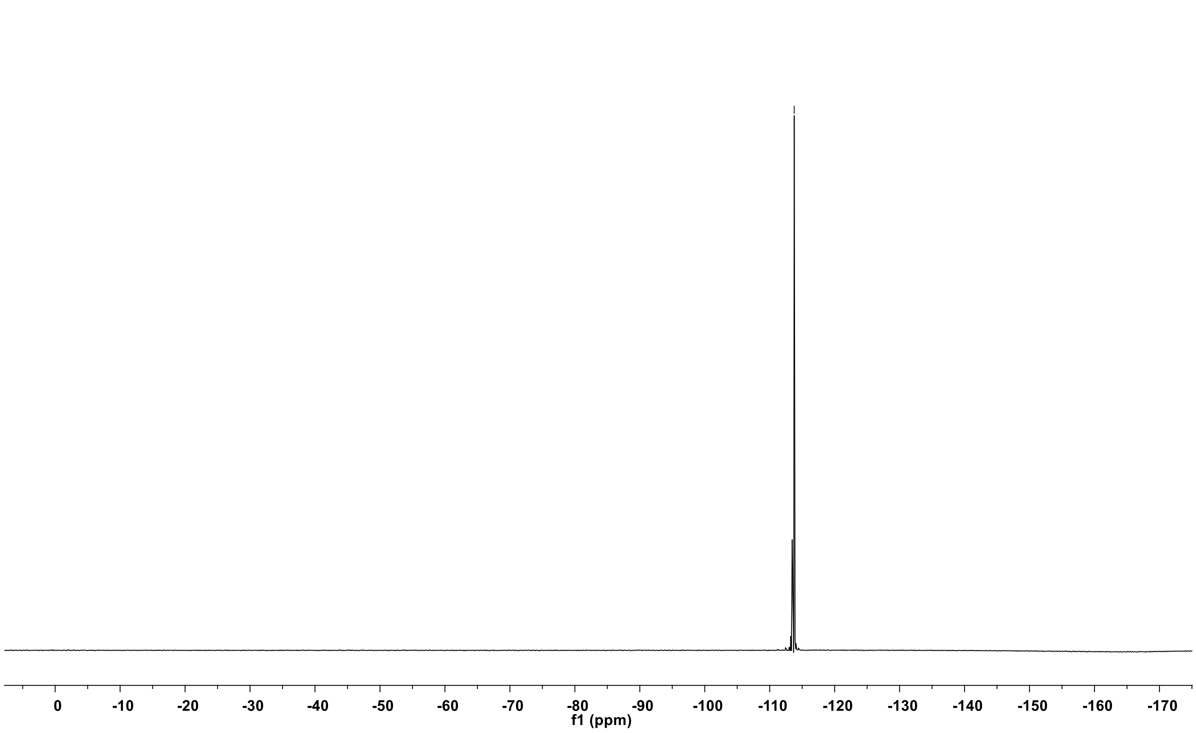

^{19}F NMR in $CDCl_3$ at 470 MHz

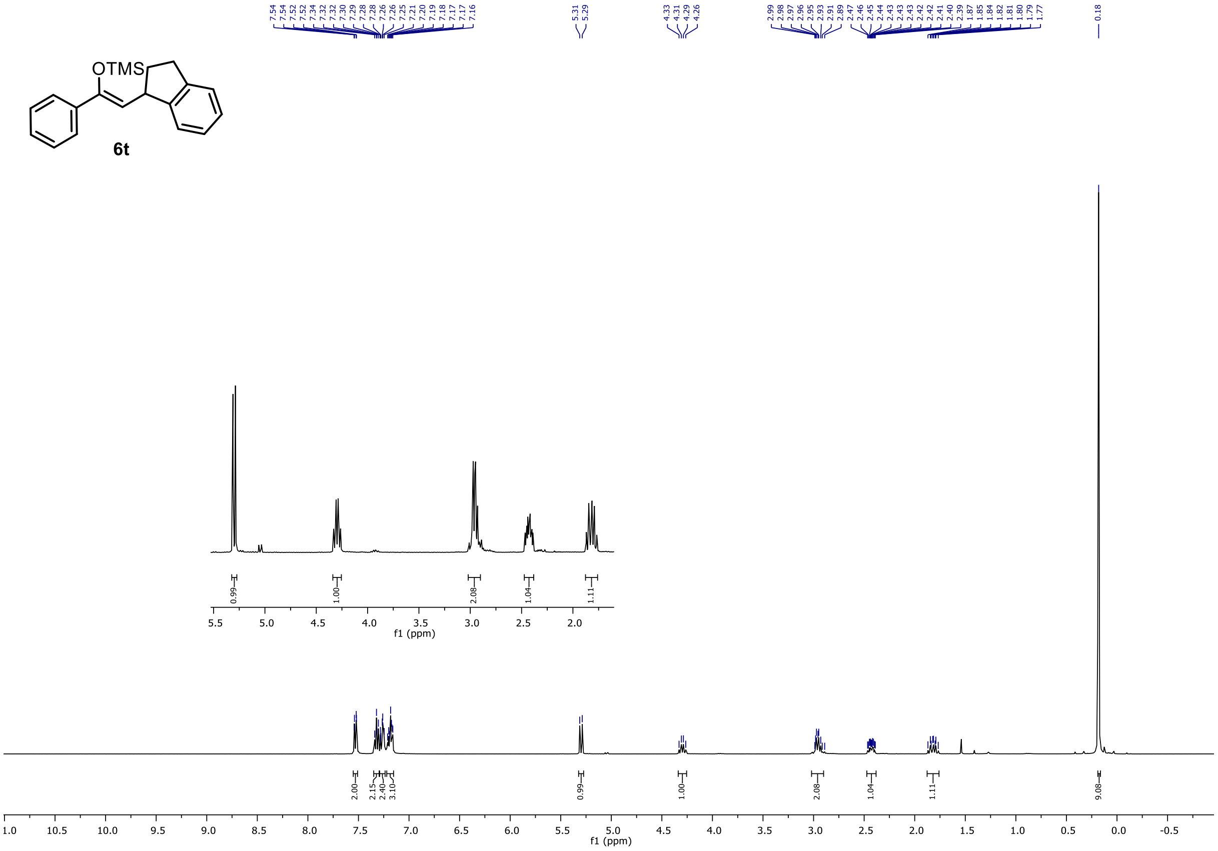

^{1}H NMR in $CDCl_3$ at 400 MHz

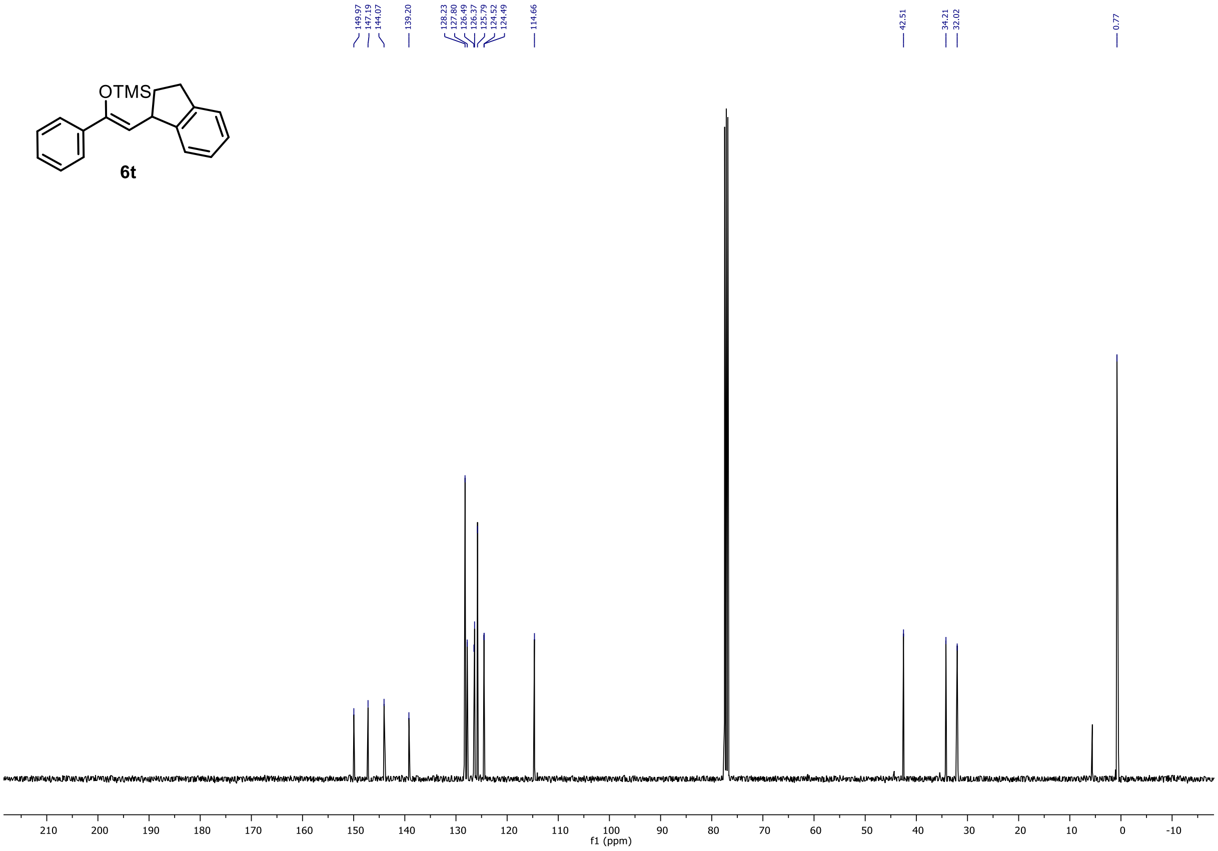

^{13}C NMR in $CDCl_3$ at 101 MHz

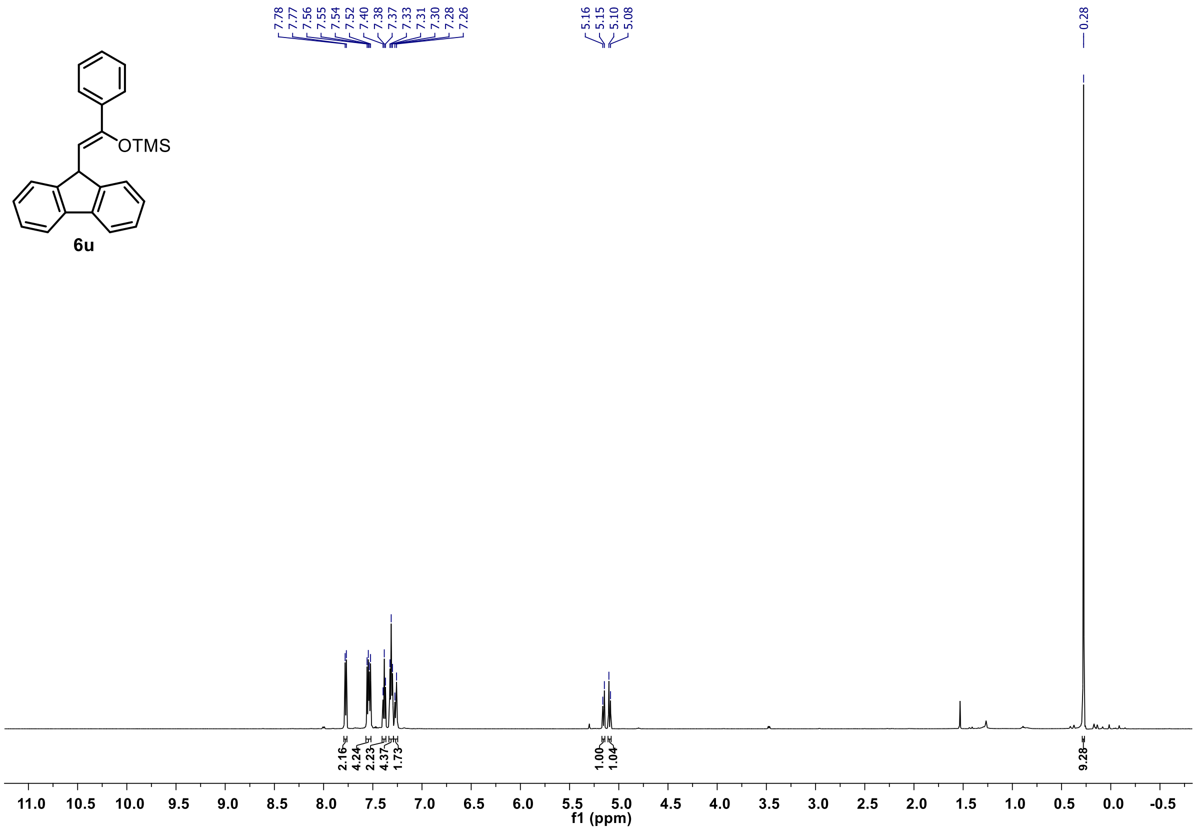

^{1}H NMR in $CDCl_3$ at 600 MHz

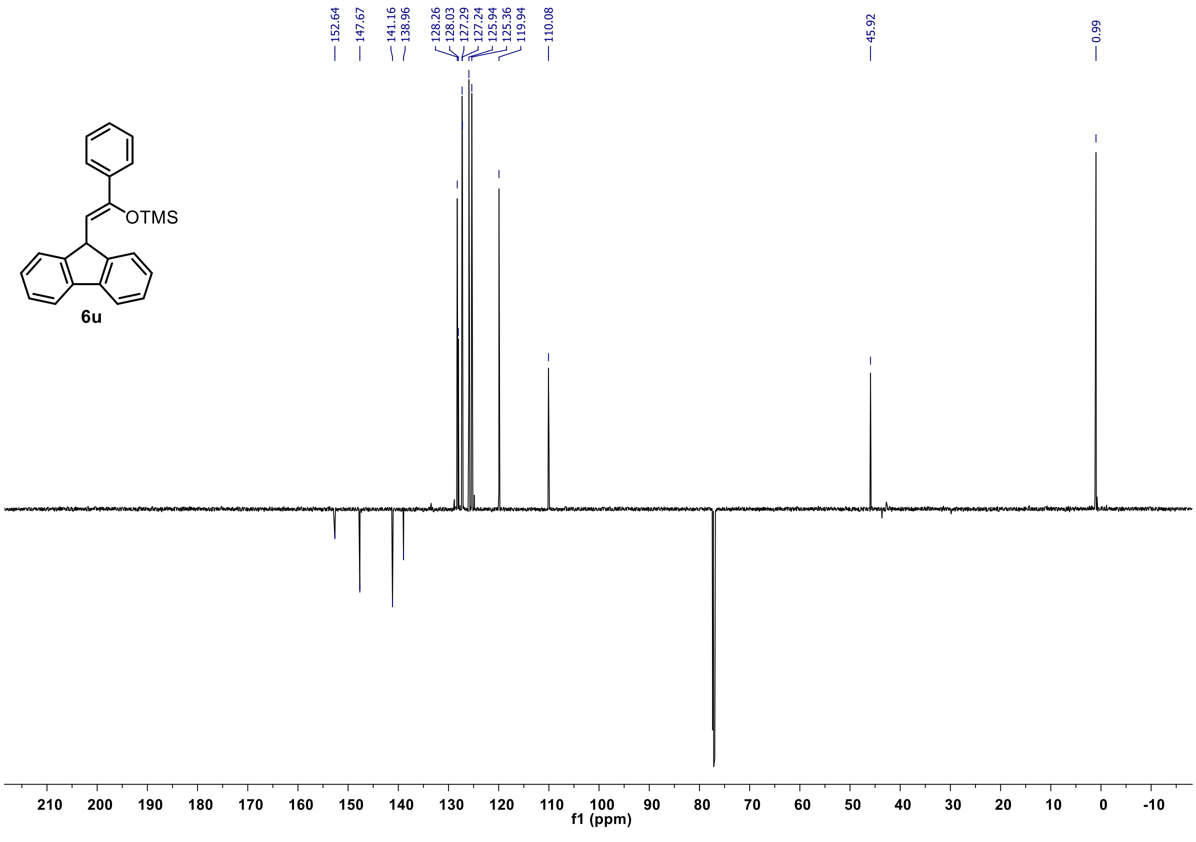

^{13}C NMR in $CDCl_3$ at 151 MHz

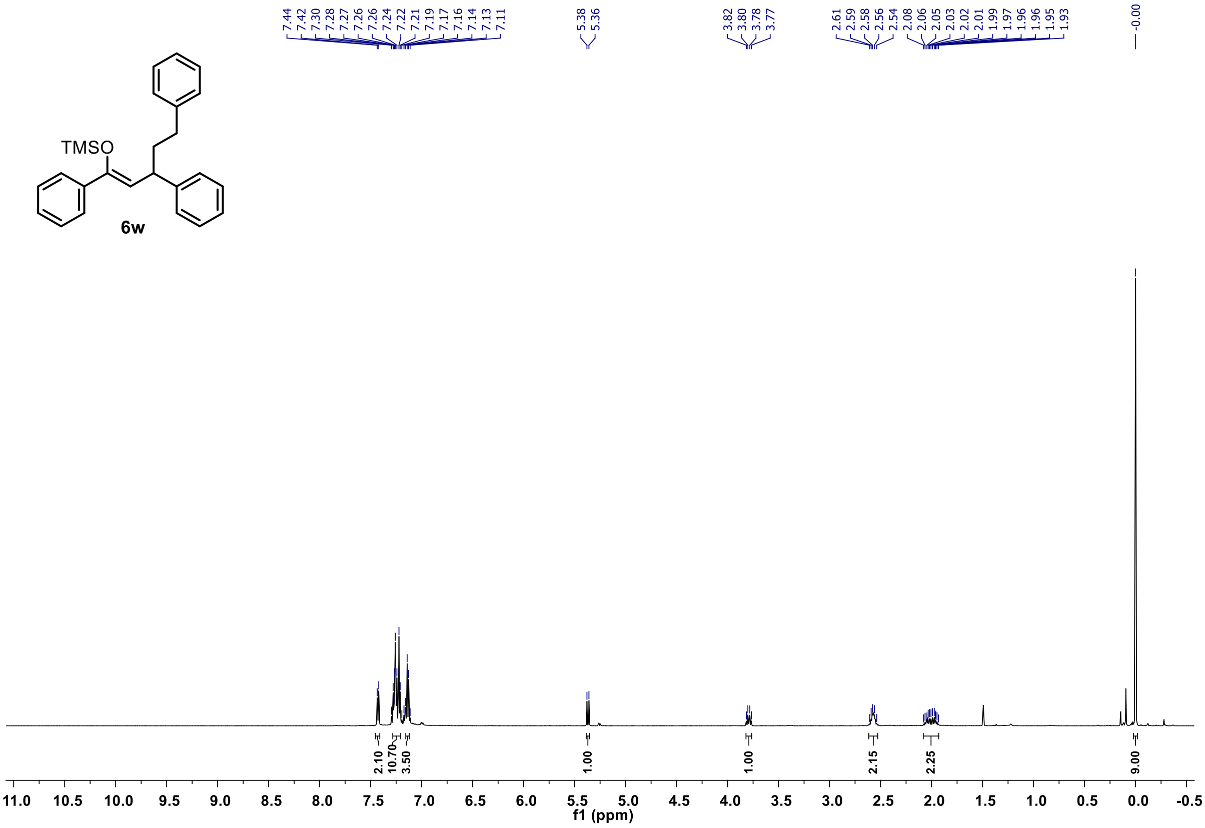

^{1}H NMR in $CDCl_3$ at 500 MHz

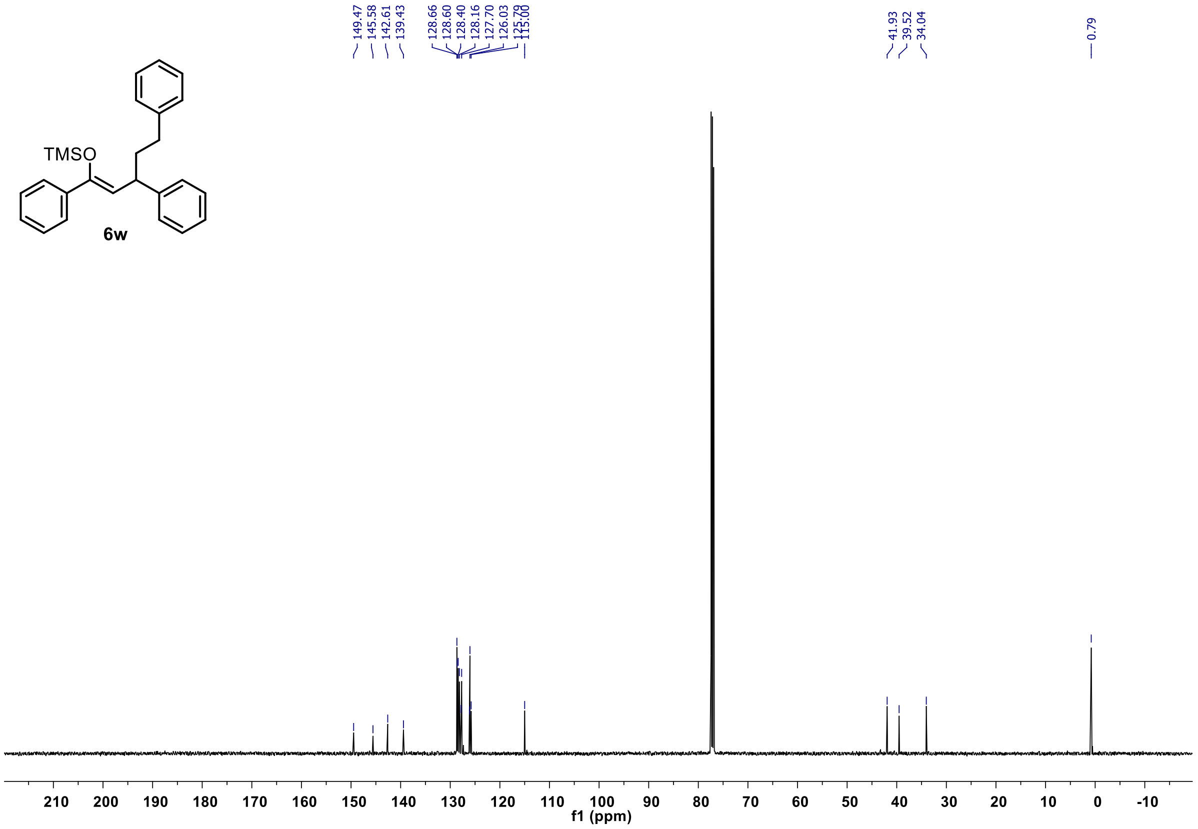

^{13}C NMR in $CDCl_3$ at 126 MHz

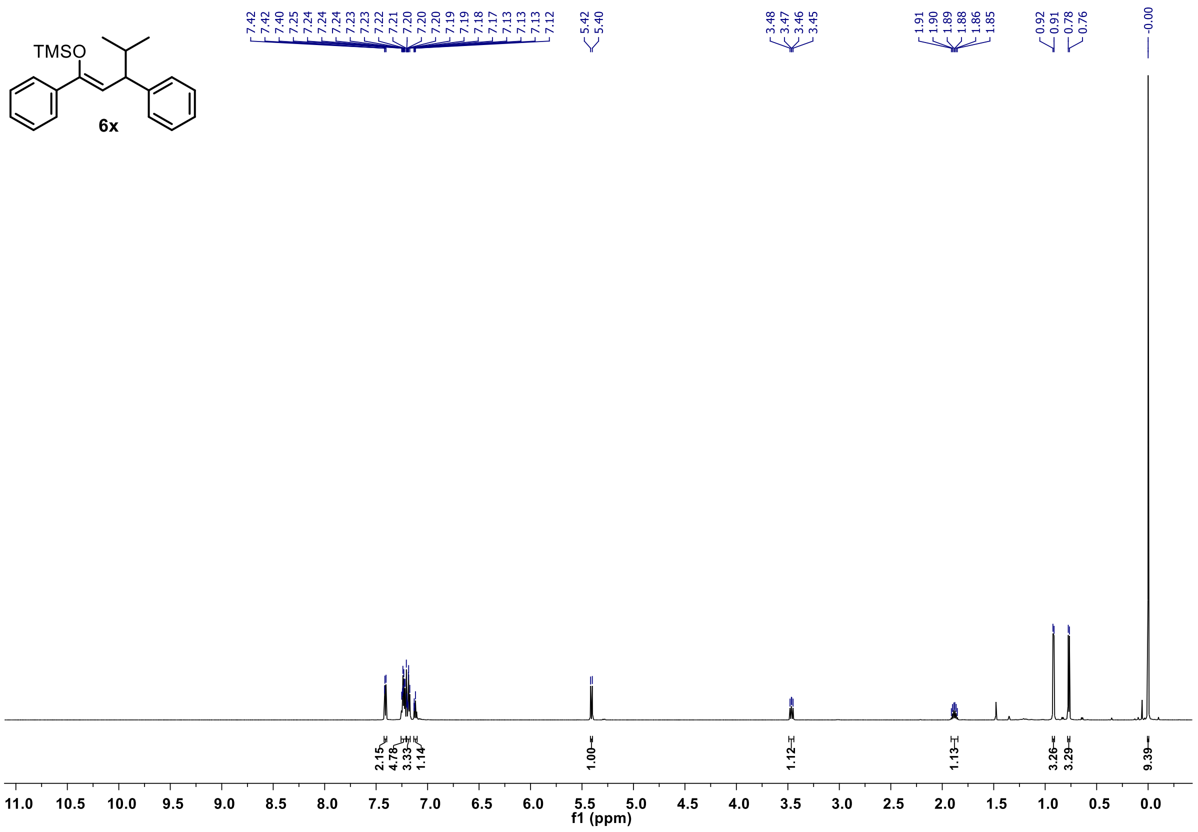

^{1}H NMR in $CDCl_3$ at 600 MHz

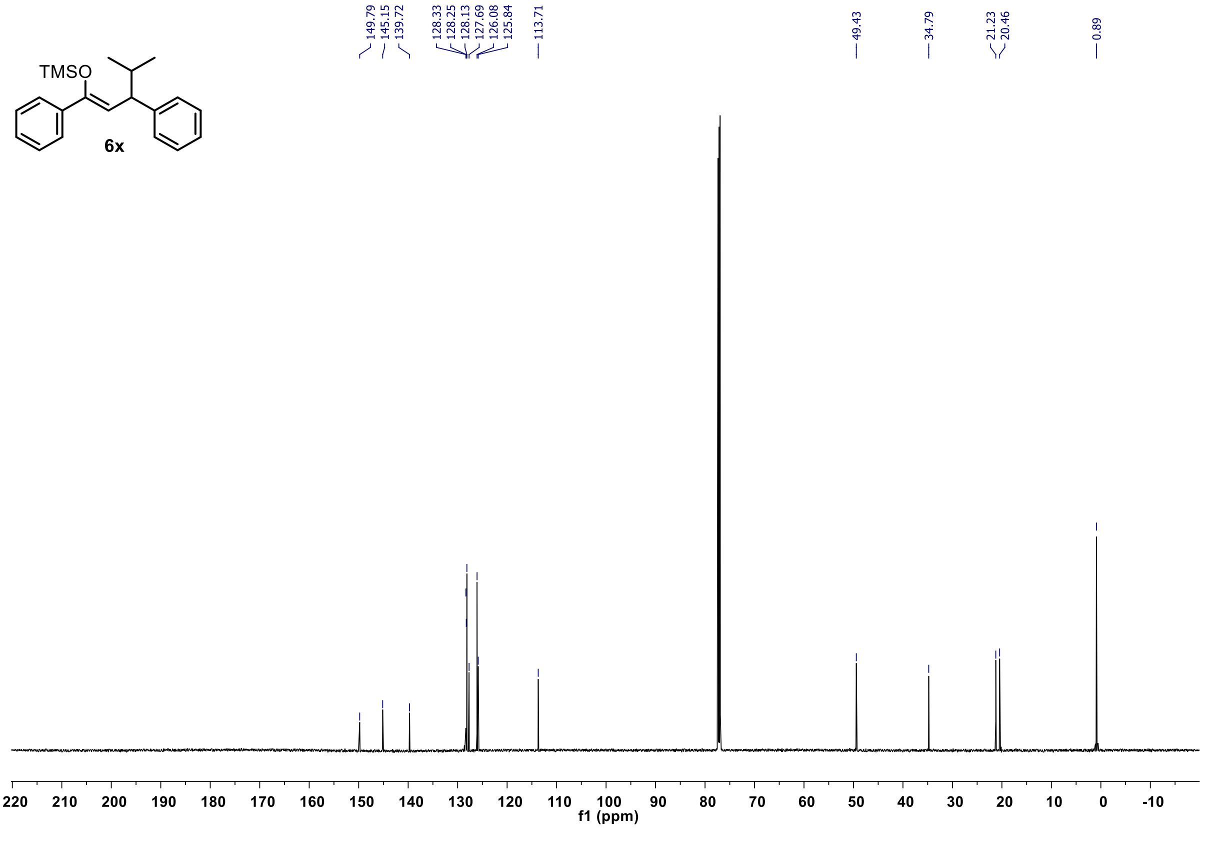

^{13}C NMR in $CDCl_3$ at 151 MHz

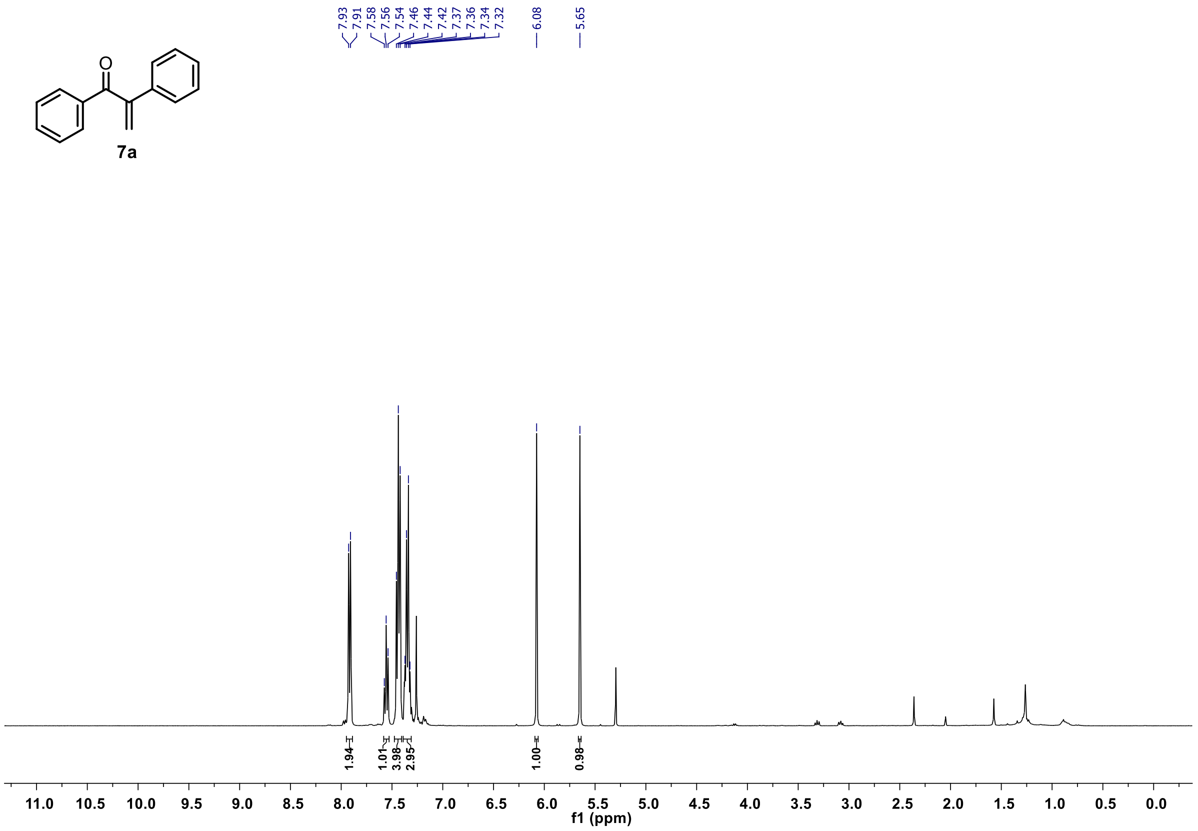

^{1}H NMR in $CDCl_3$ at 400 MHz

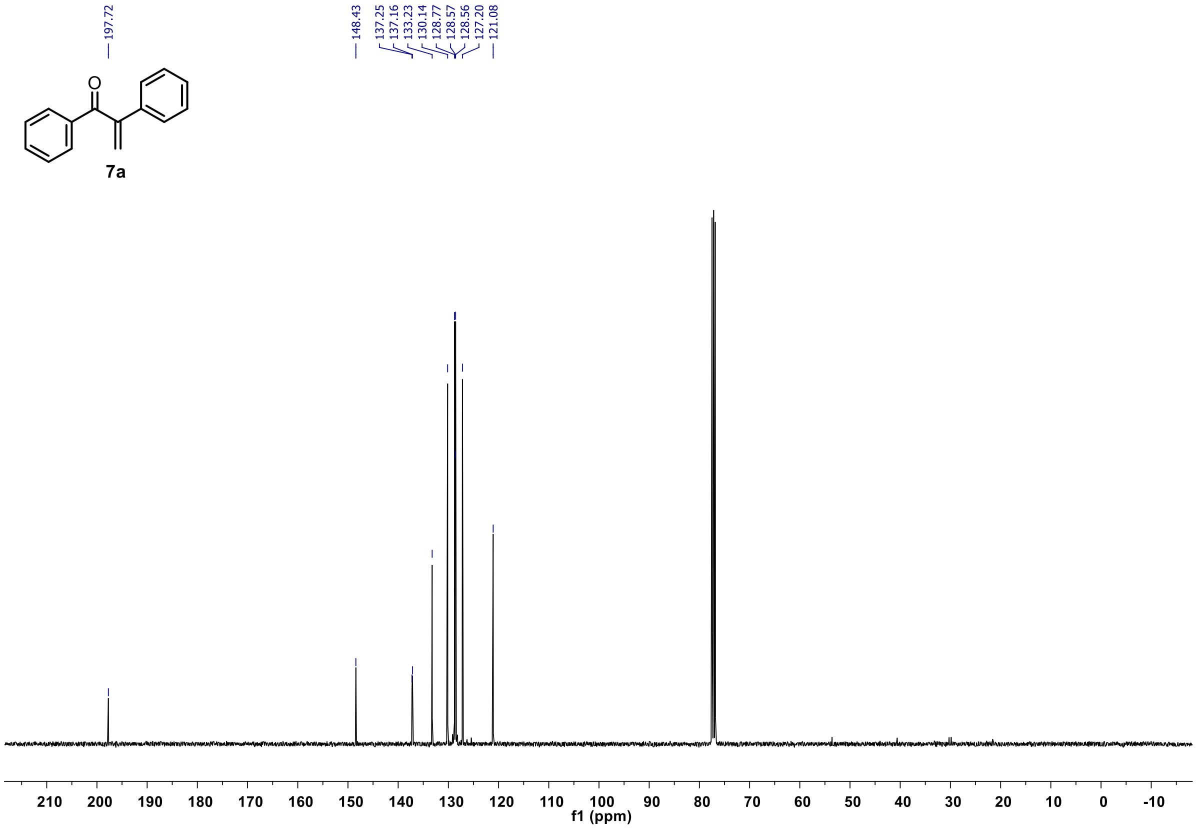

^{13}C NMR in $CDCl_3$ at 101 MHz

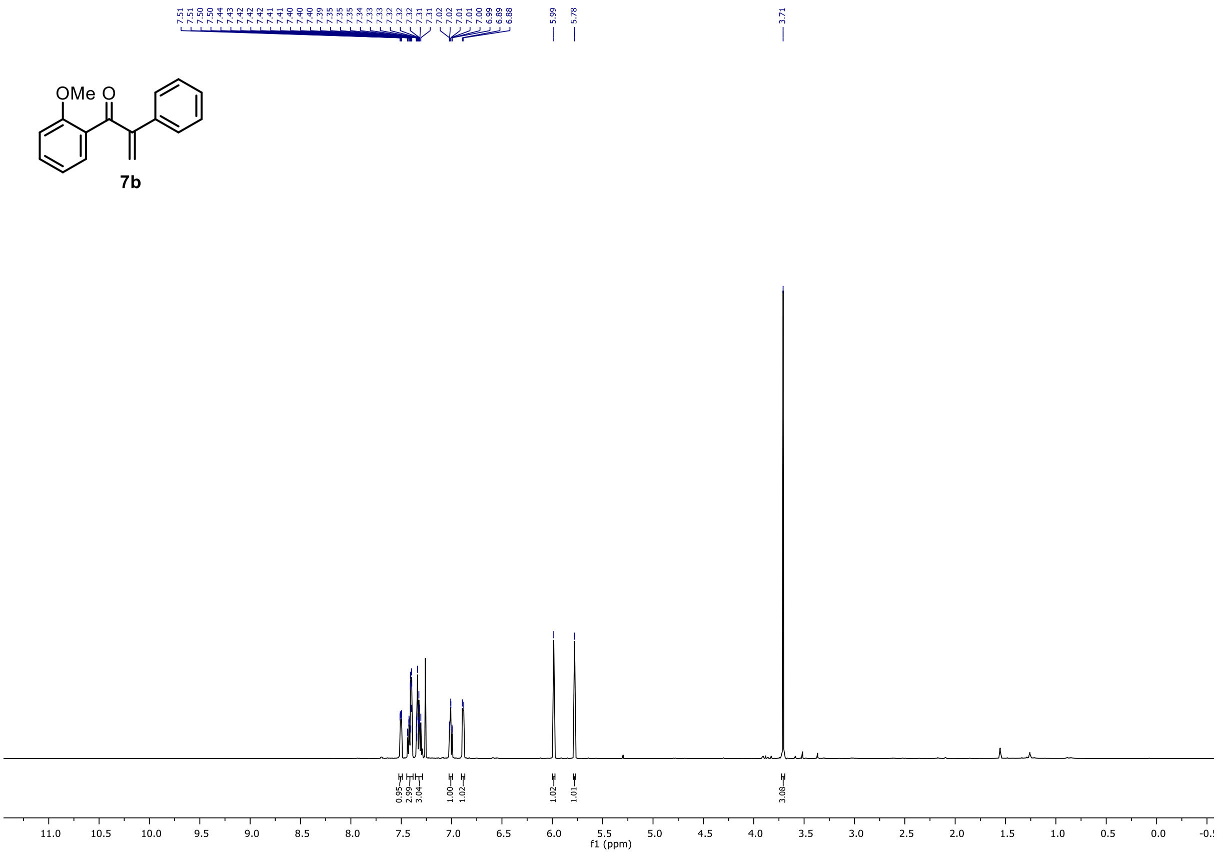

^{1}H NMR in $CDCl_3$ at 600 MHz

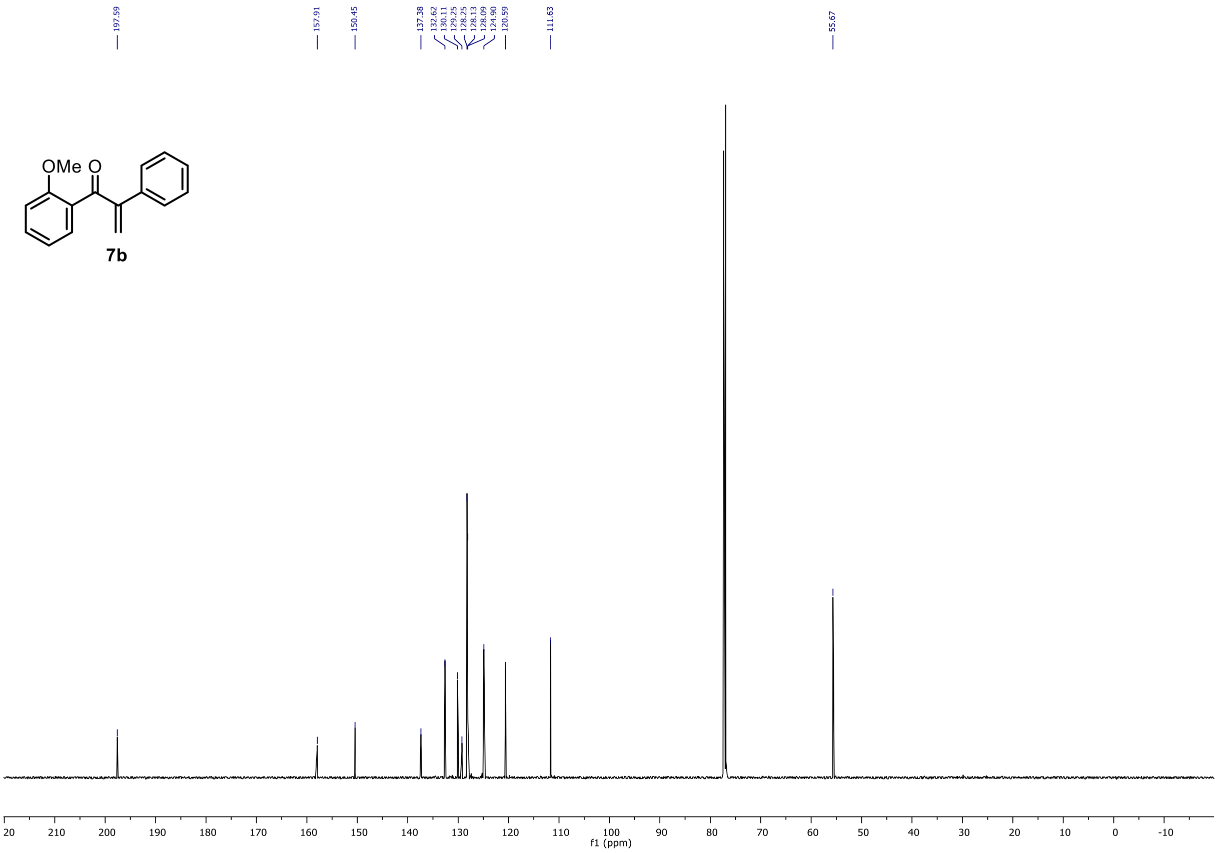

^{13}C NMR in $CDCl_3$ at 151 MHz

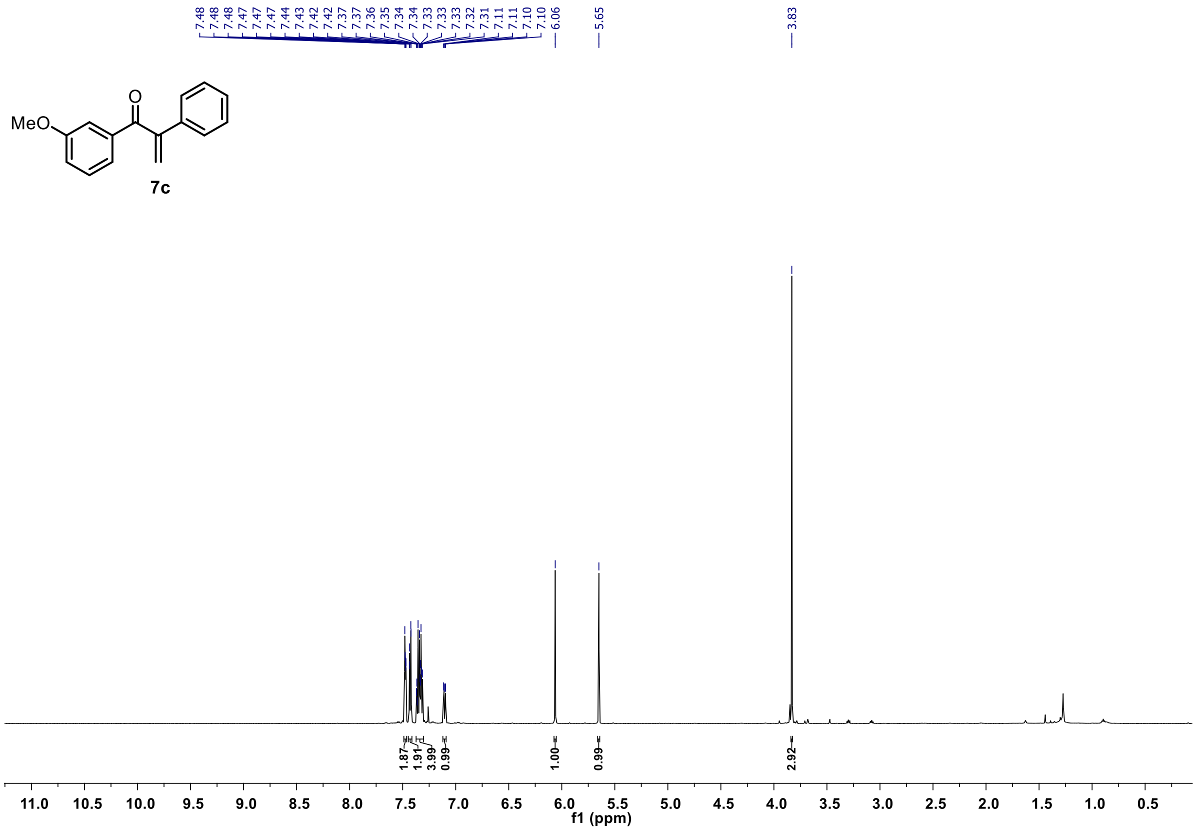

^{1}H NMR in $CDCl_3$ at 600 MHz

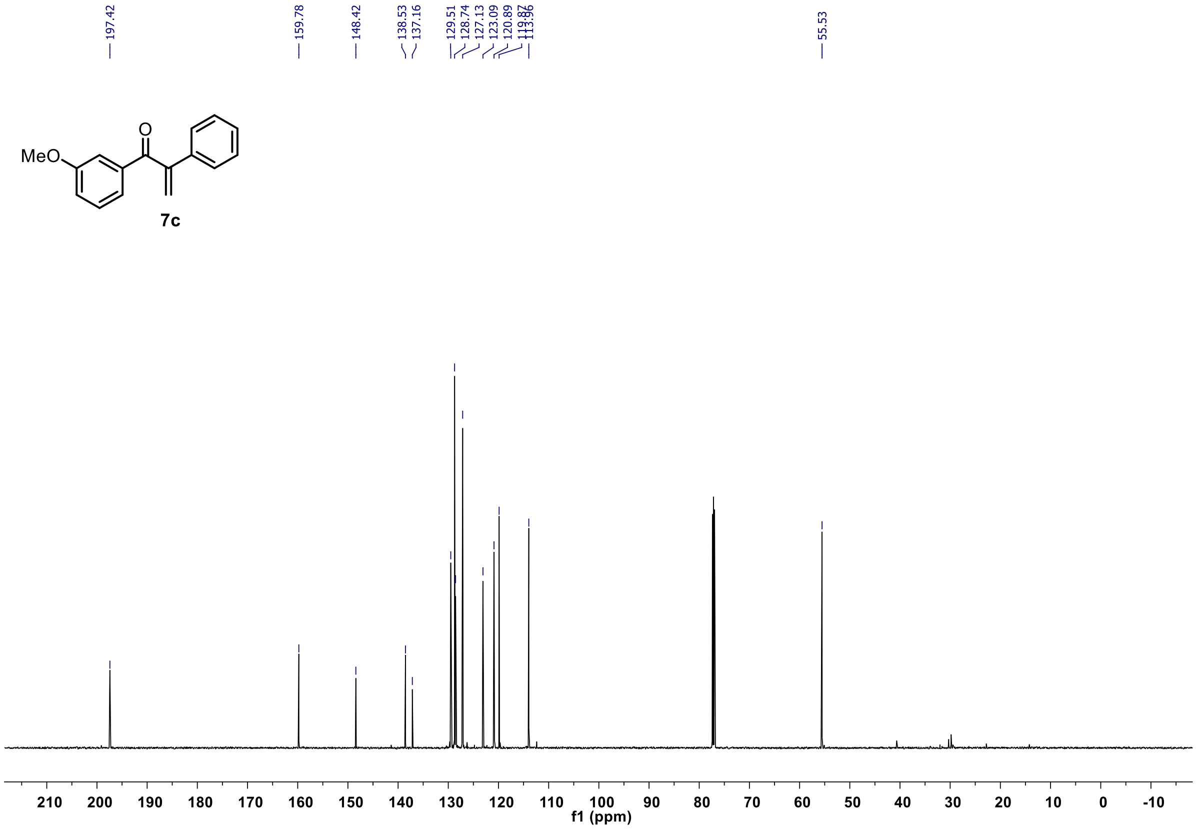

^{13}C NMR in $CDCl_3$ at 151 MHz

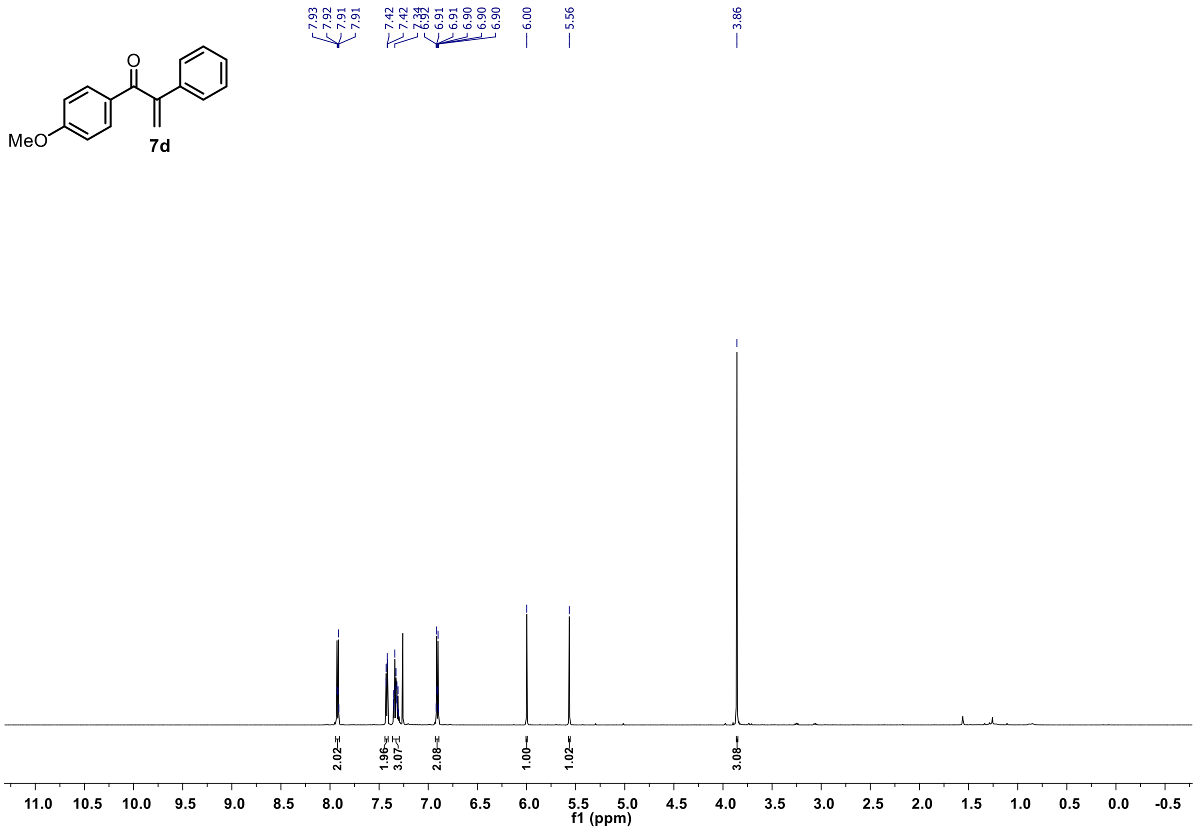

^{1}H NMR in $CDCl_3$ at 600 MHz

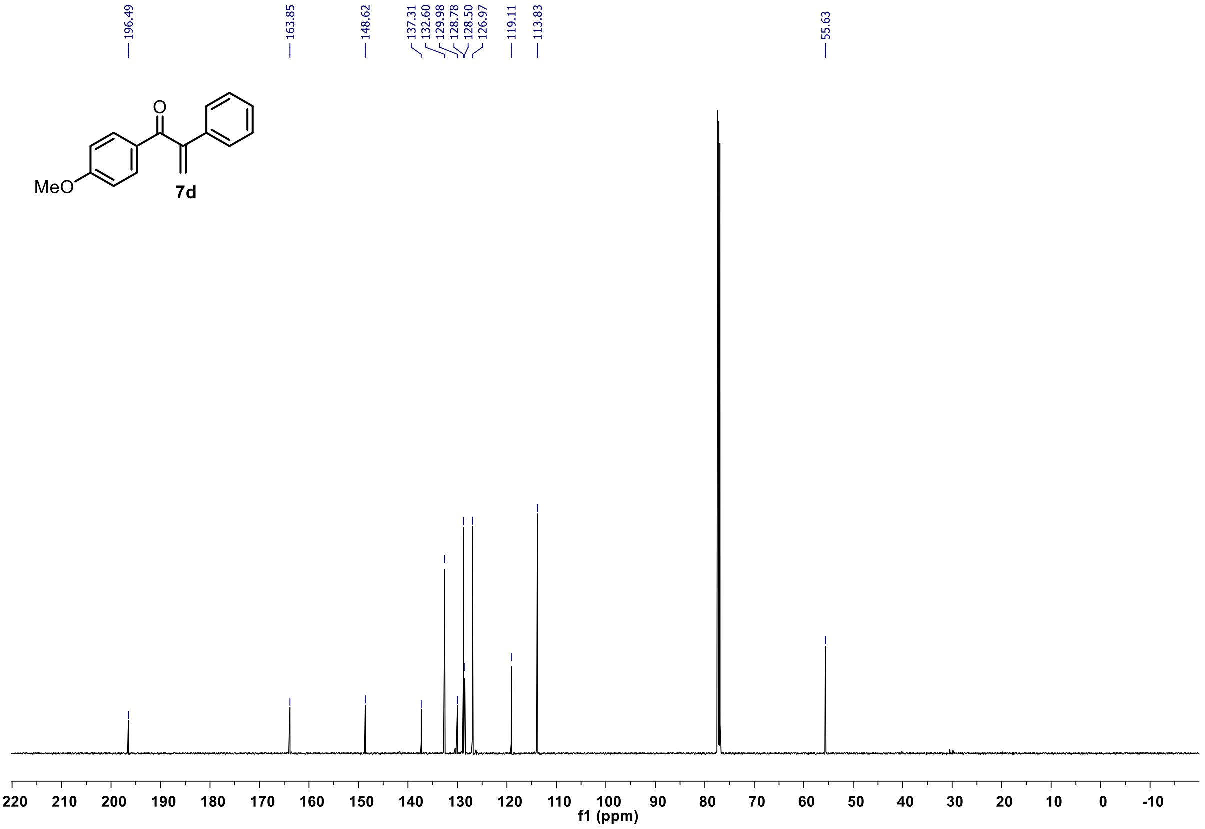

^{13}C NMR in $CDCl_3$ at 151 MHz

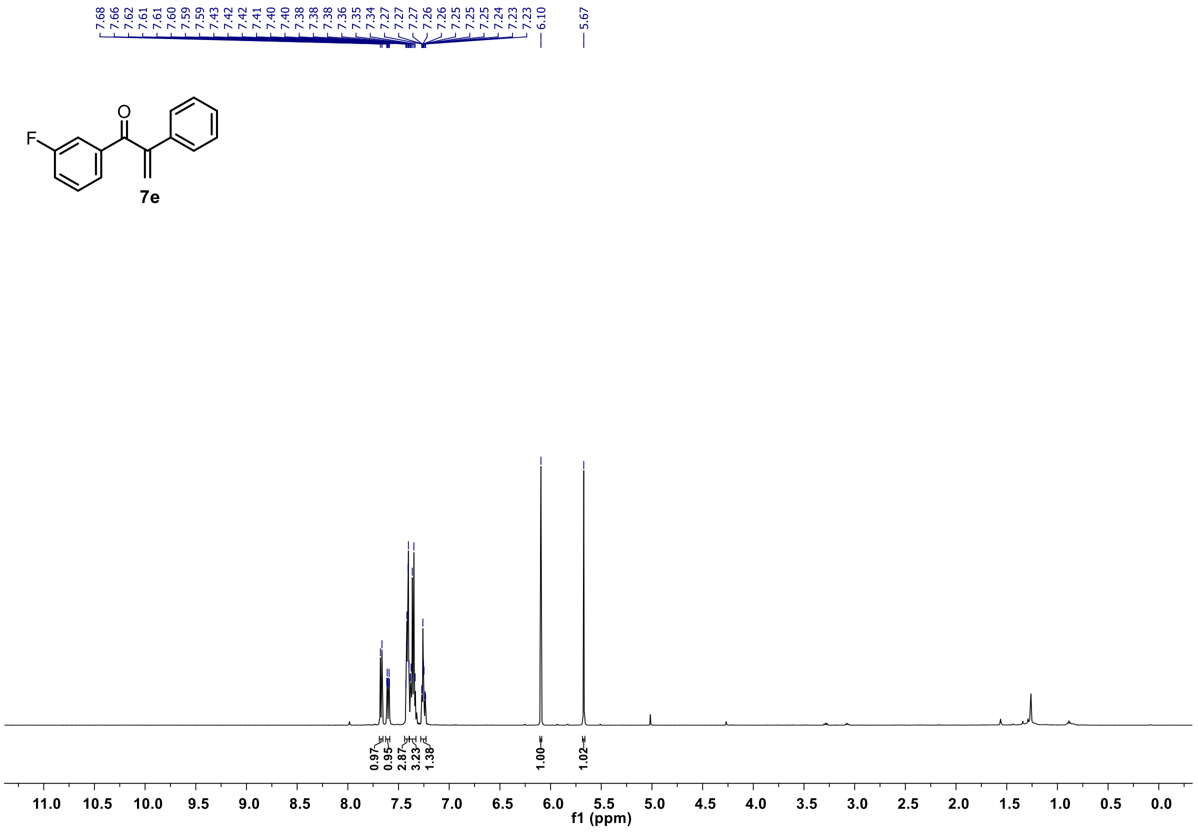

^{1}H NMR in $CDCl_3$ at 500 MHz

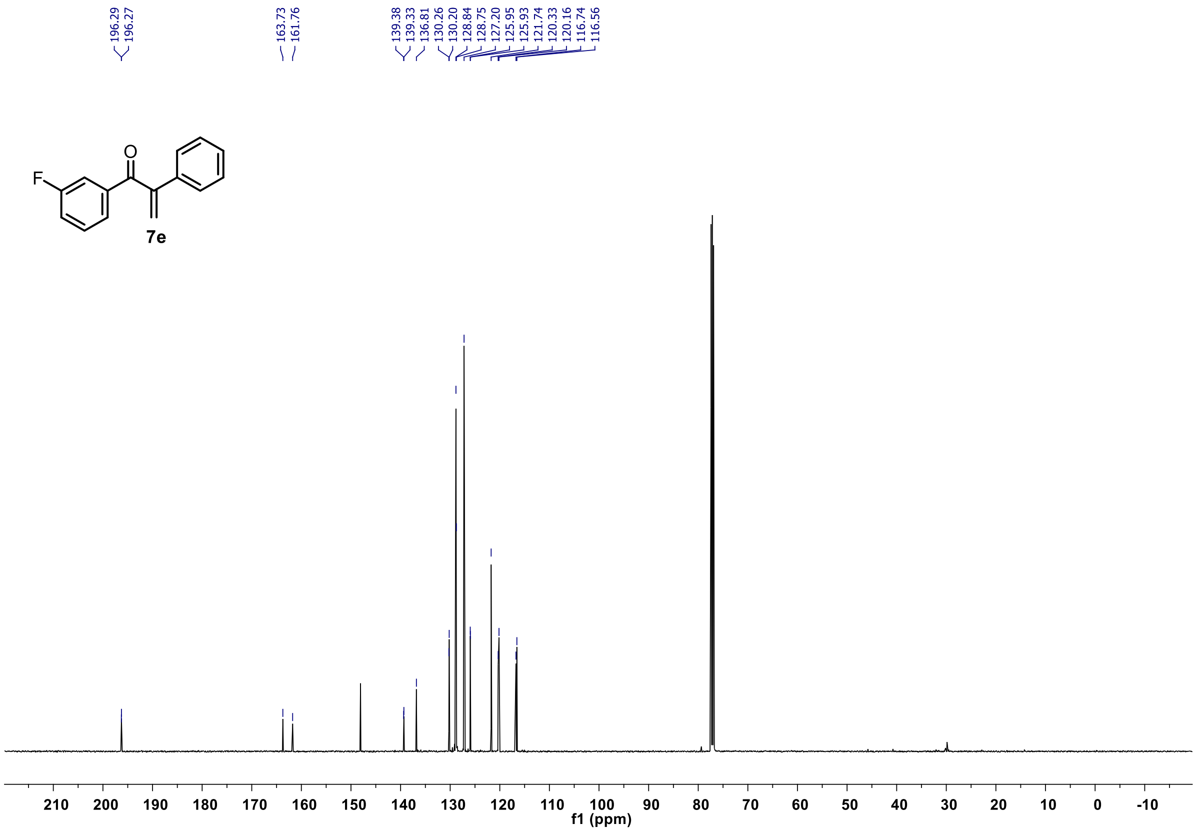

^{13}C NMR in $CDCl_3$ at 126 MHz

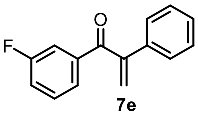

7e

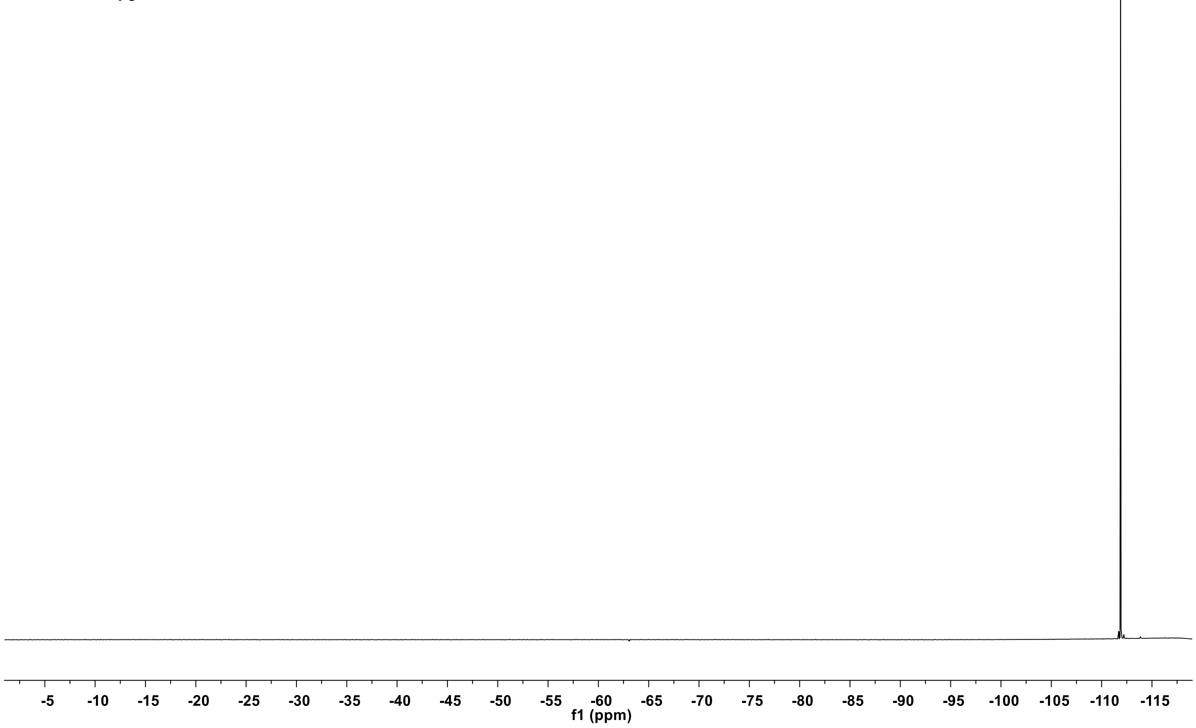

^{19}F NMR in $CDCl_3$ at 471 MHz

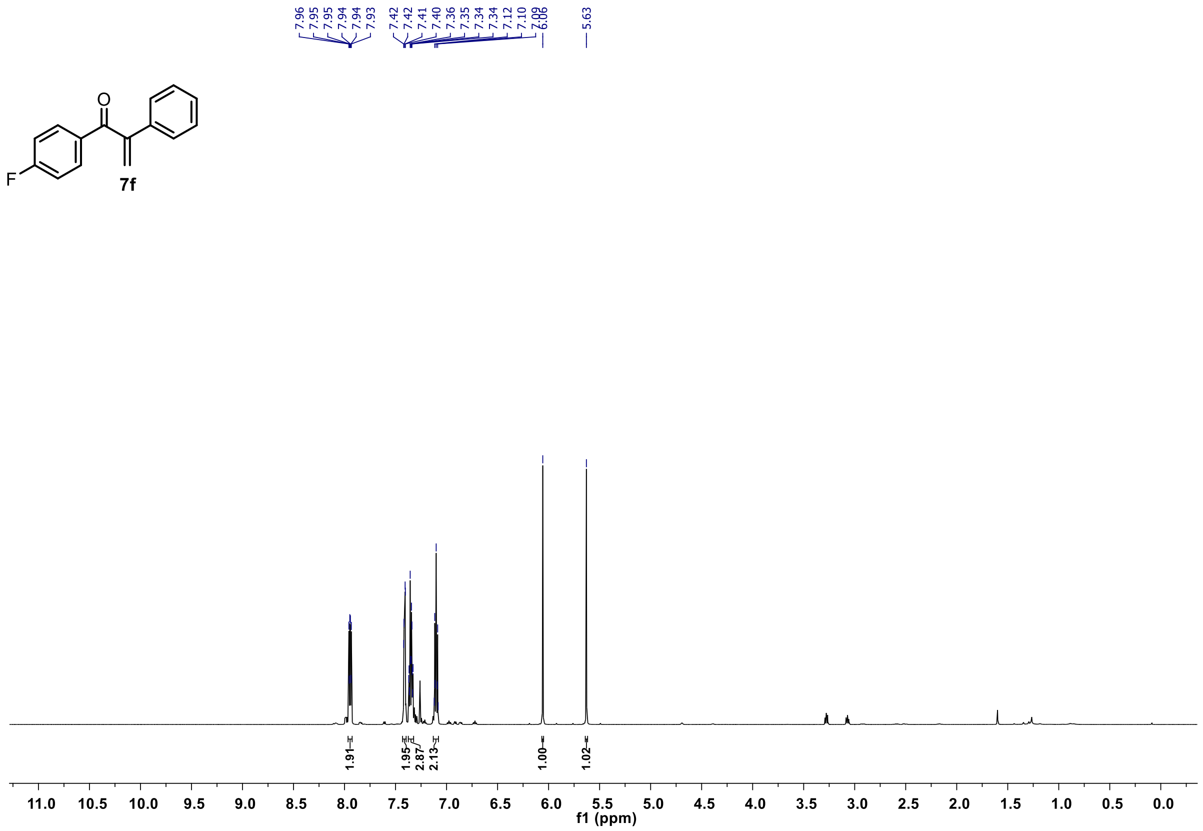

^{1}H NMR in $CDCl_3$ at 600 MHz

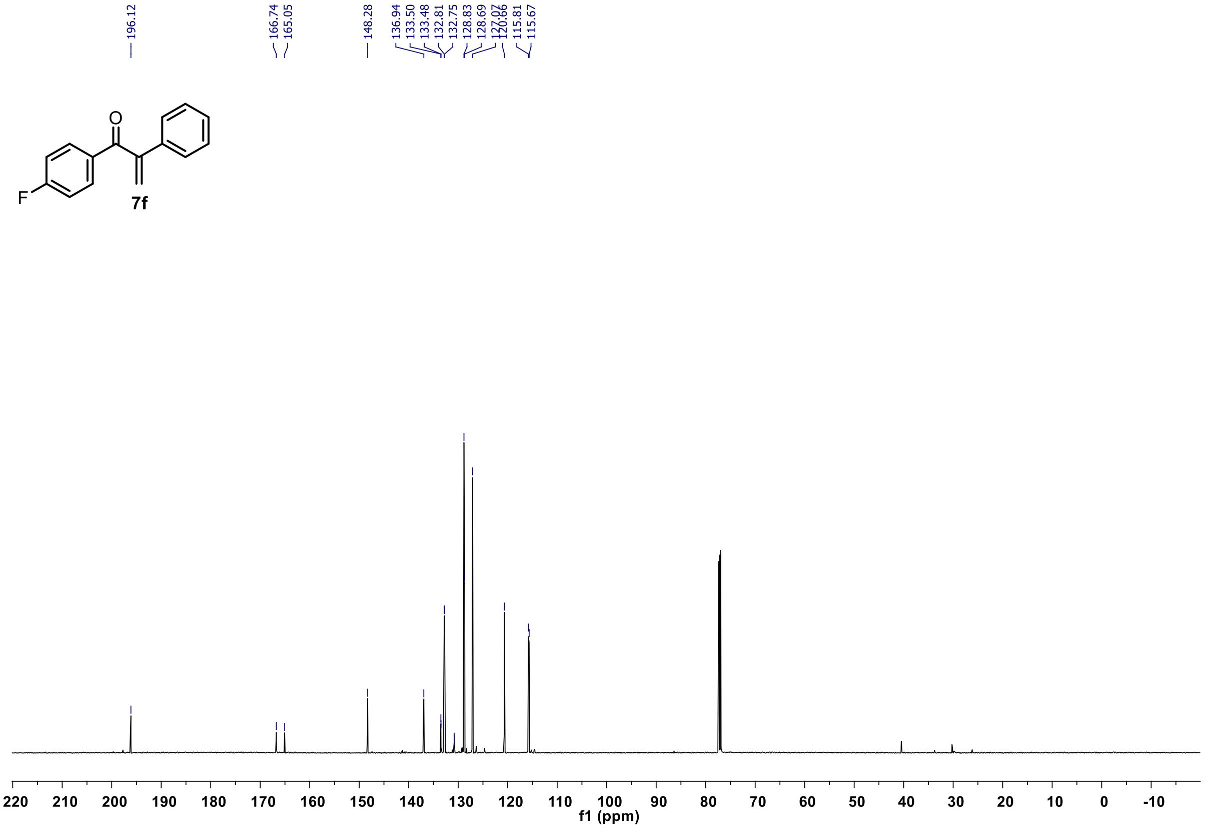

^{13}C NMR in $CDCl_3$ at 151 MHz

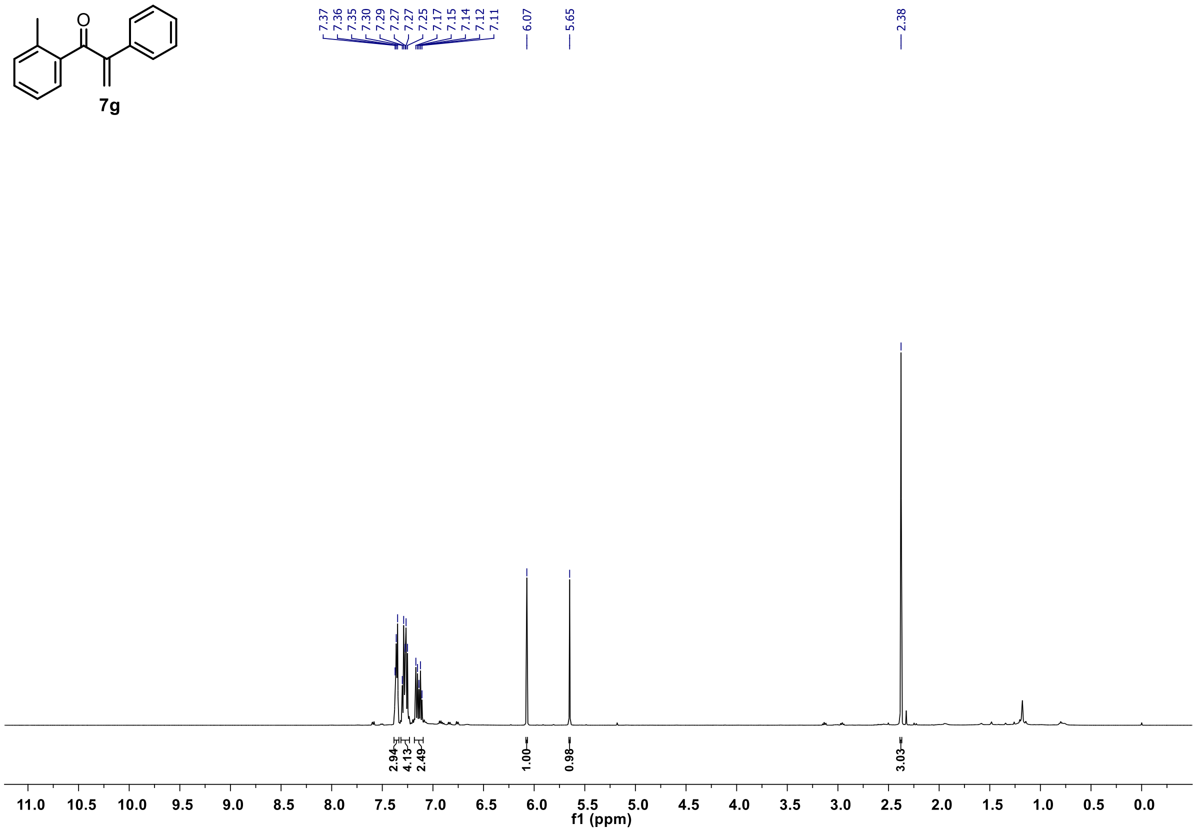

^{1}H NMR in $CDCl_3$ at 600 MHz

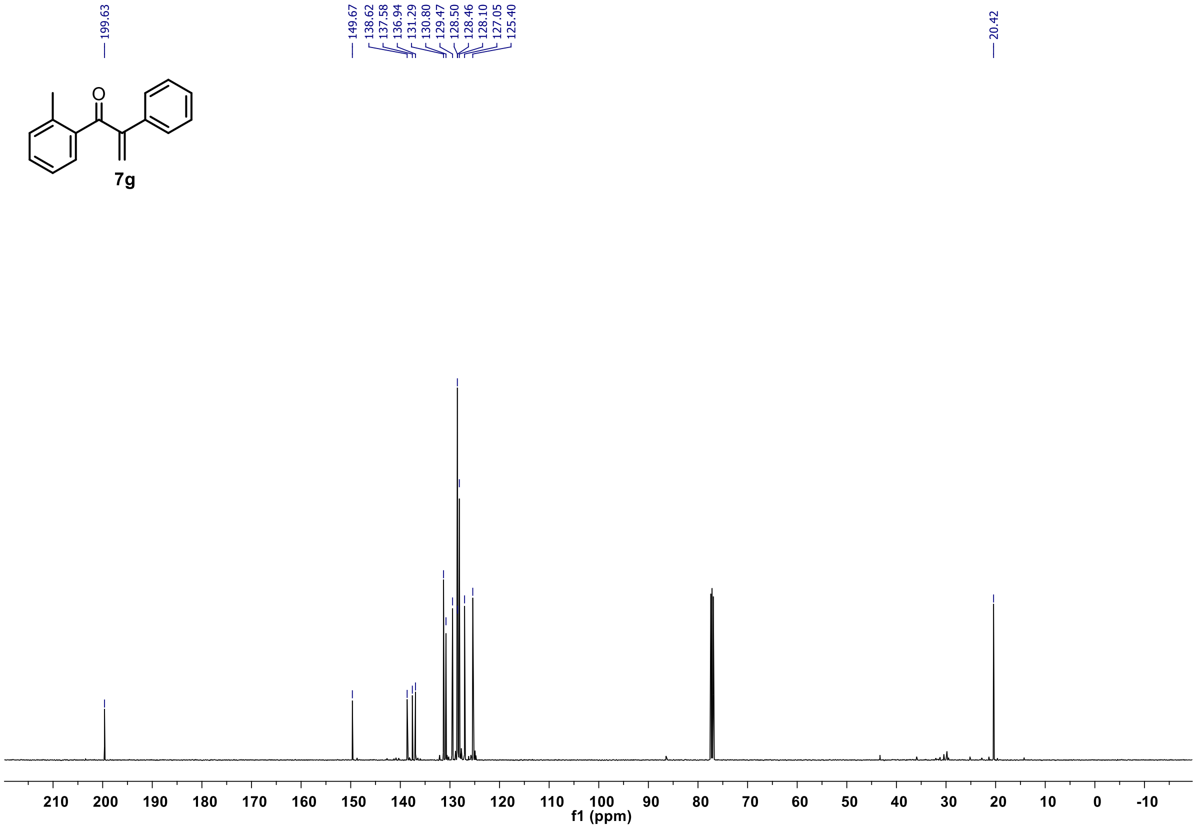

^{13}C NMR in $CDCl_3$ at 126 MHz

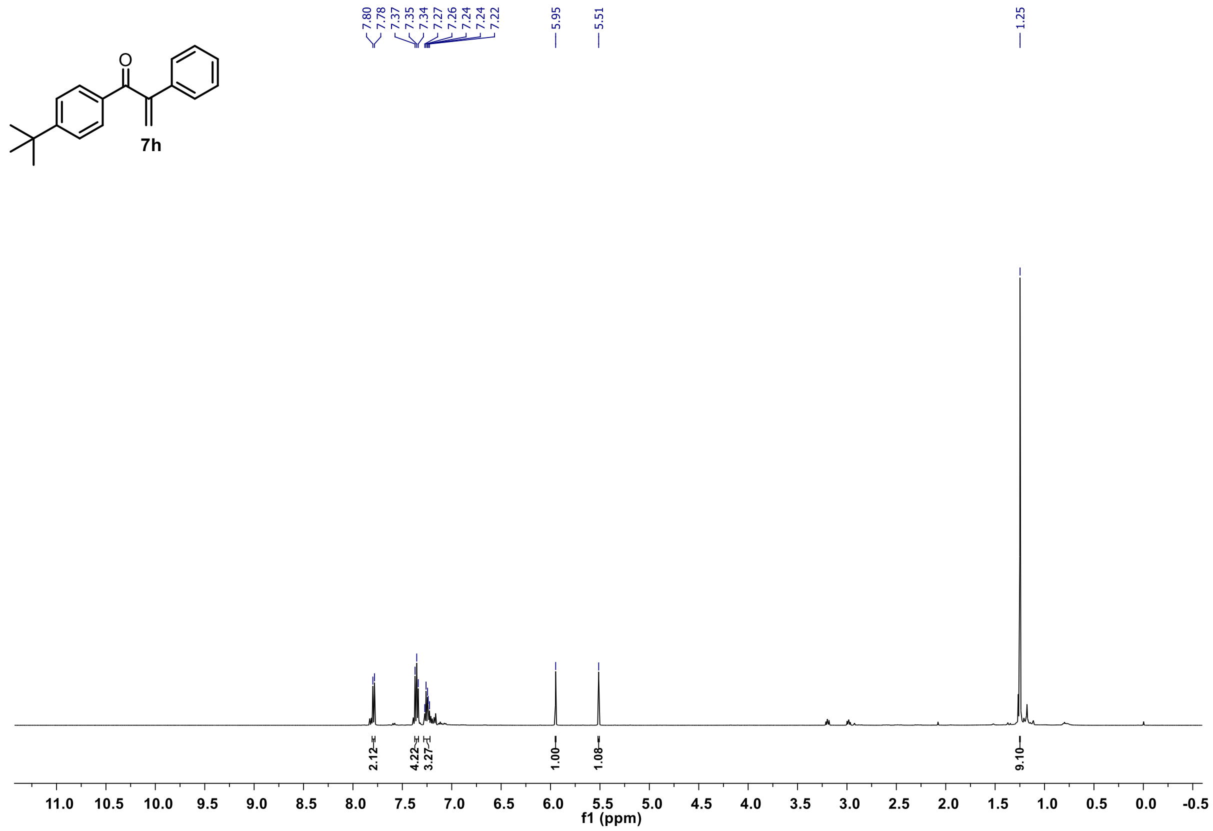

^{1}H NMR in $CDCl_3$ at 600 MHz

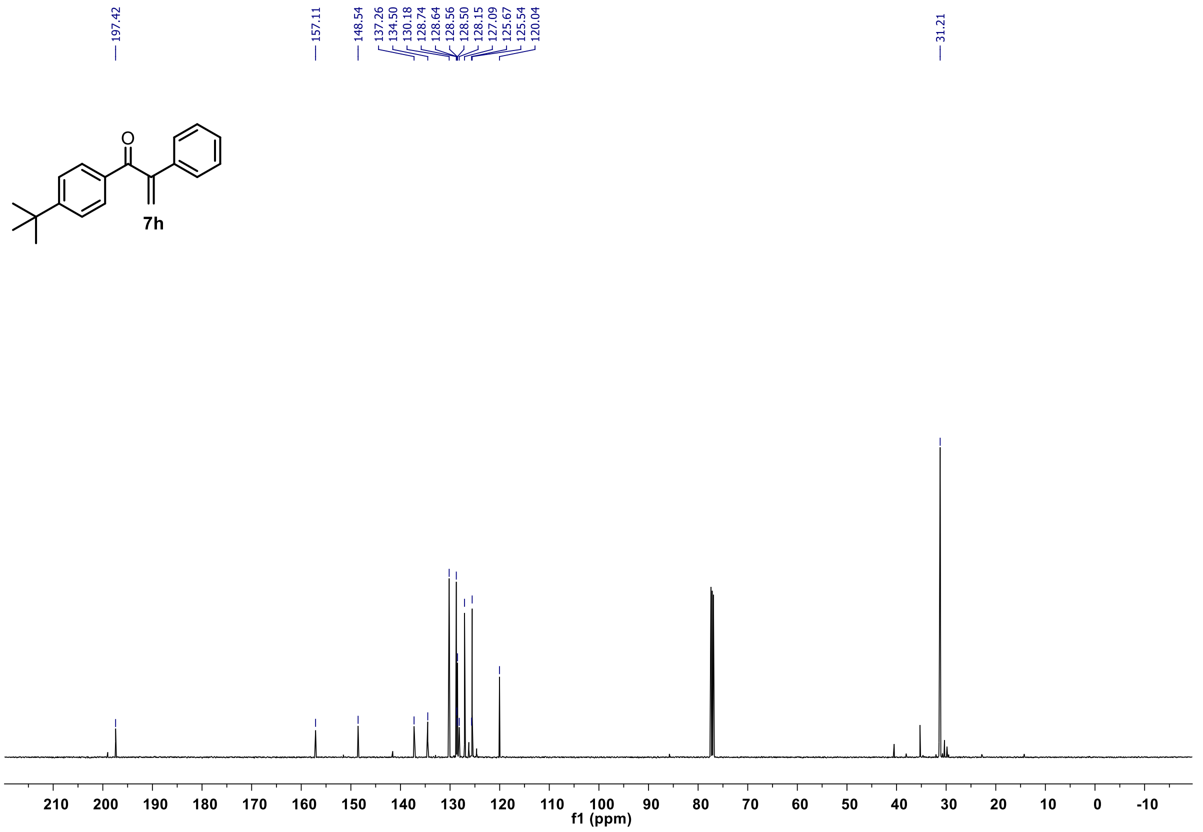

^{13}C NMR in $CDCl_3$ at 151 MHz

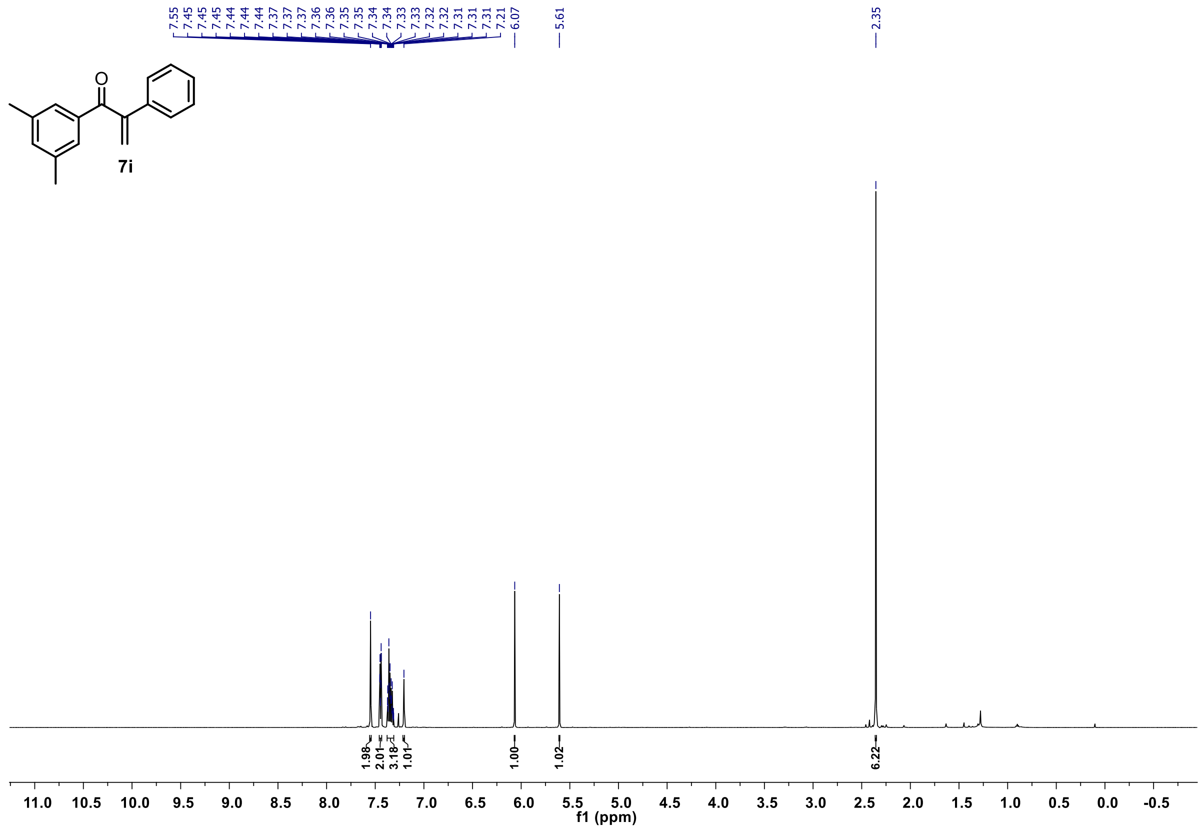

^{1}H NMR in $CDCl_3$ at 700 MHz

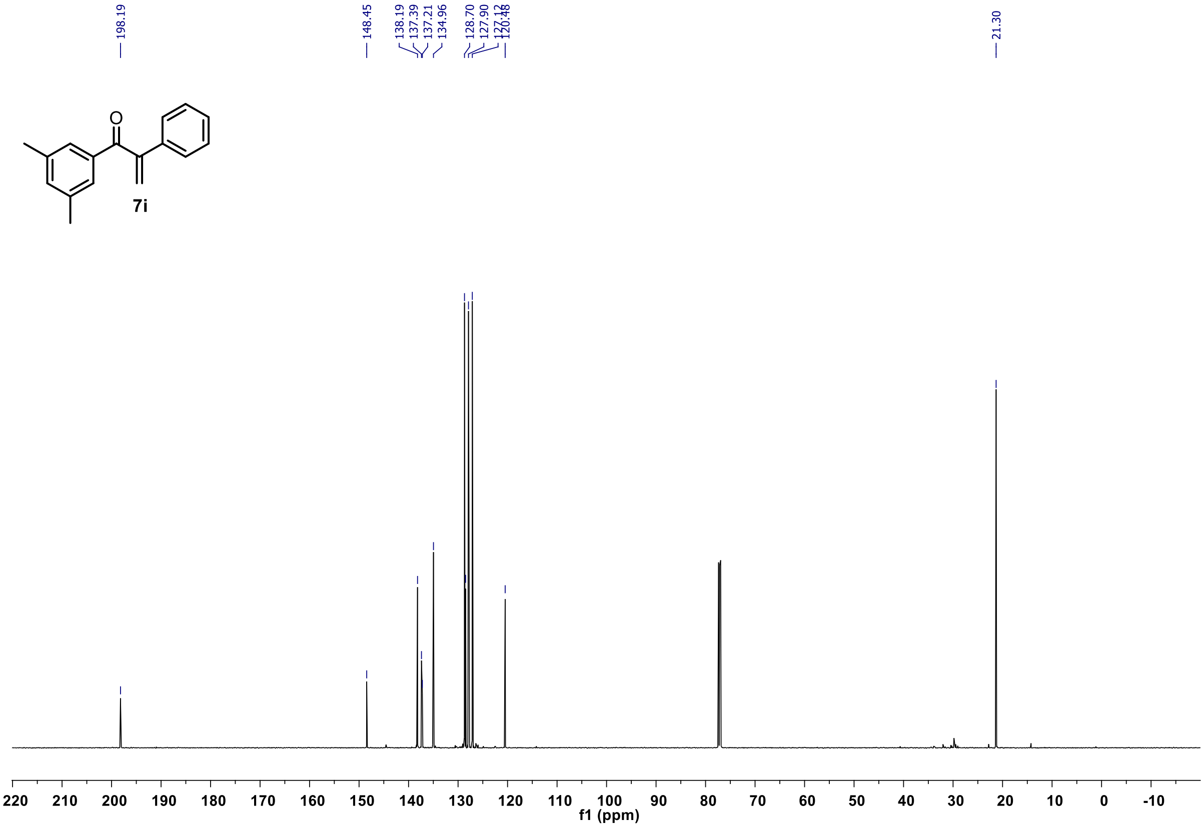

^{13}C NMR in $CDCl_3$ at 176 MHz

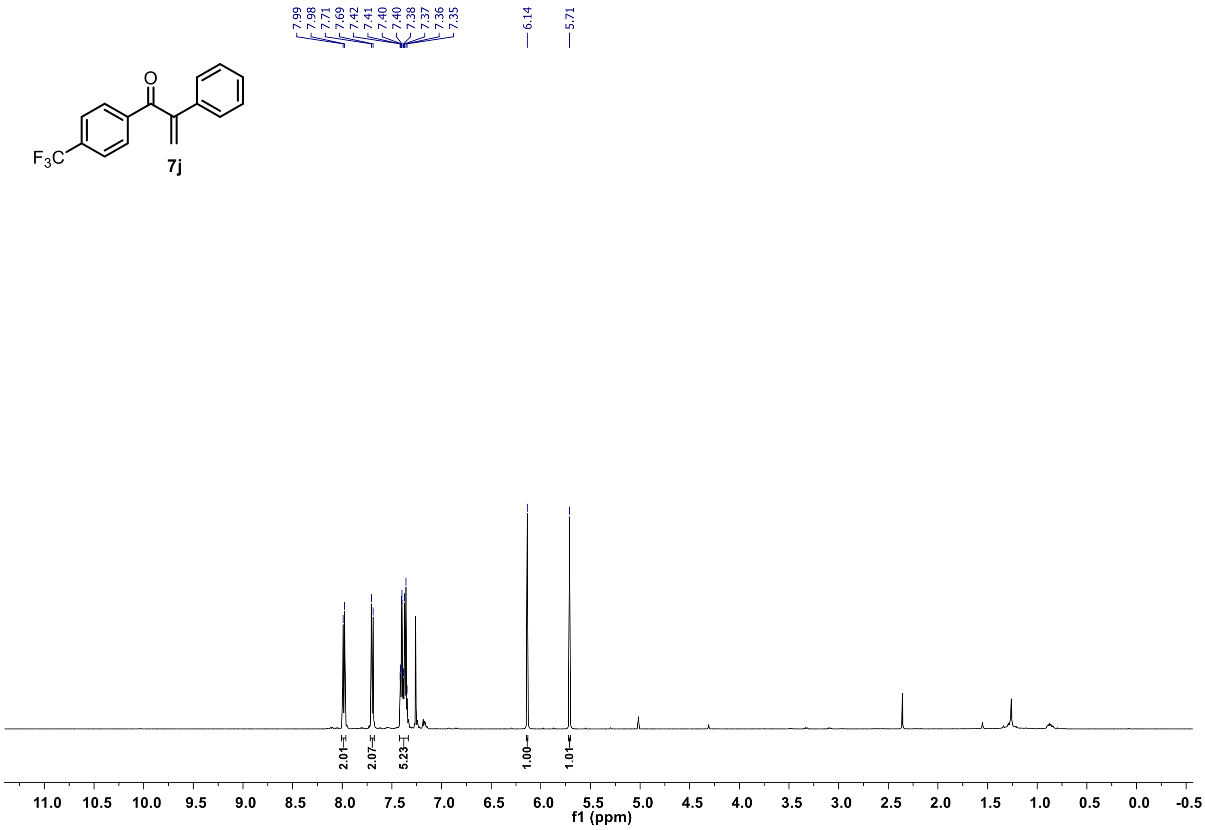

^{1}H NMR in $CDCl_3$ at 500 MHz

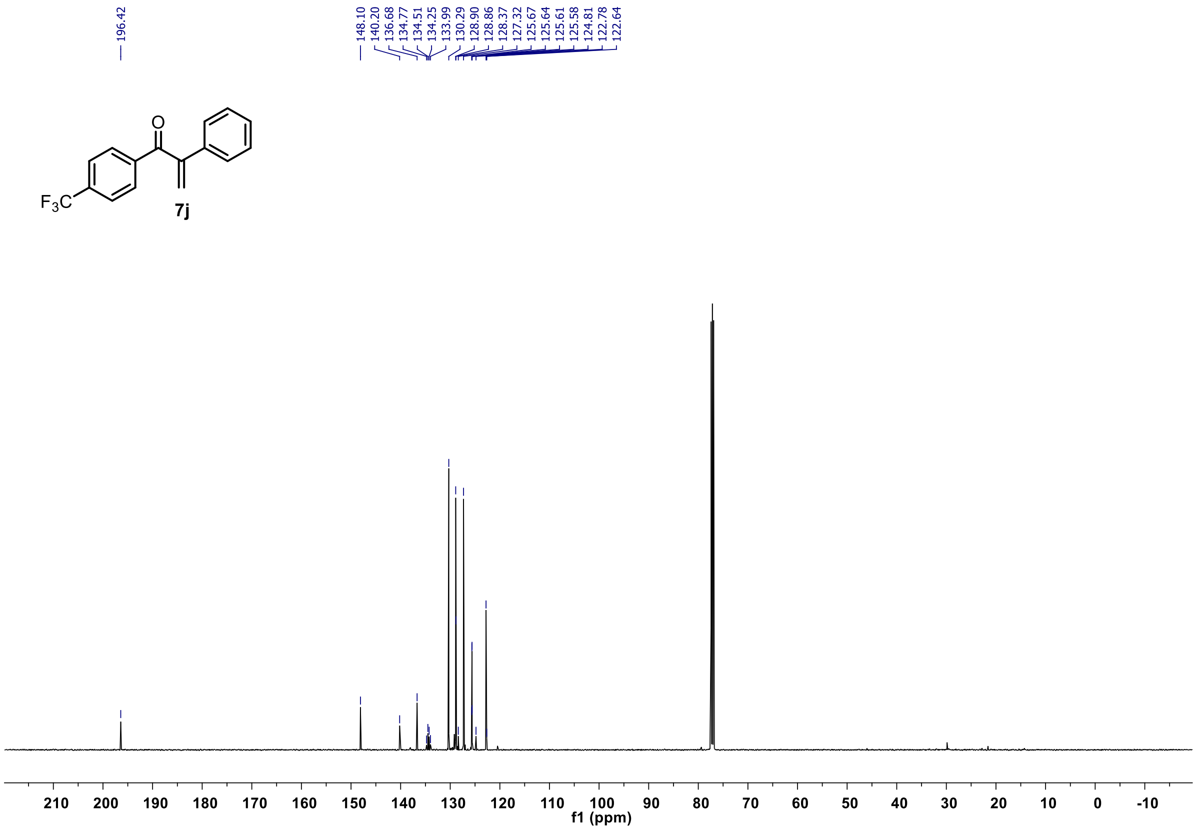

^{13}C NMR in $CDCl_3$ at 126 MHz

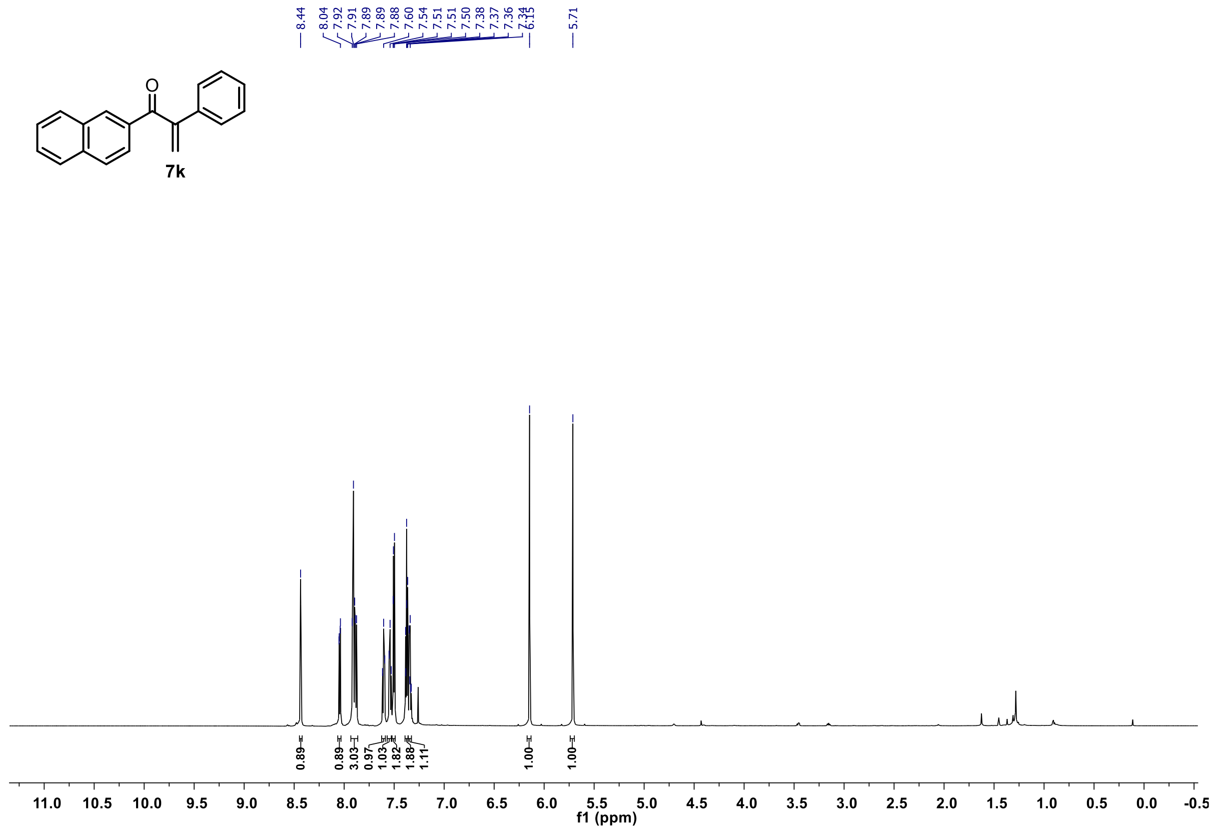

^1H NMR in $CDCl_3$ at 700 MHz

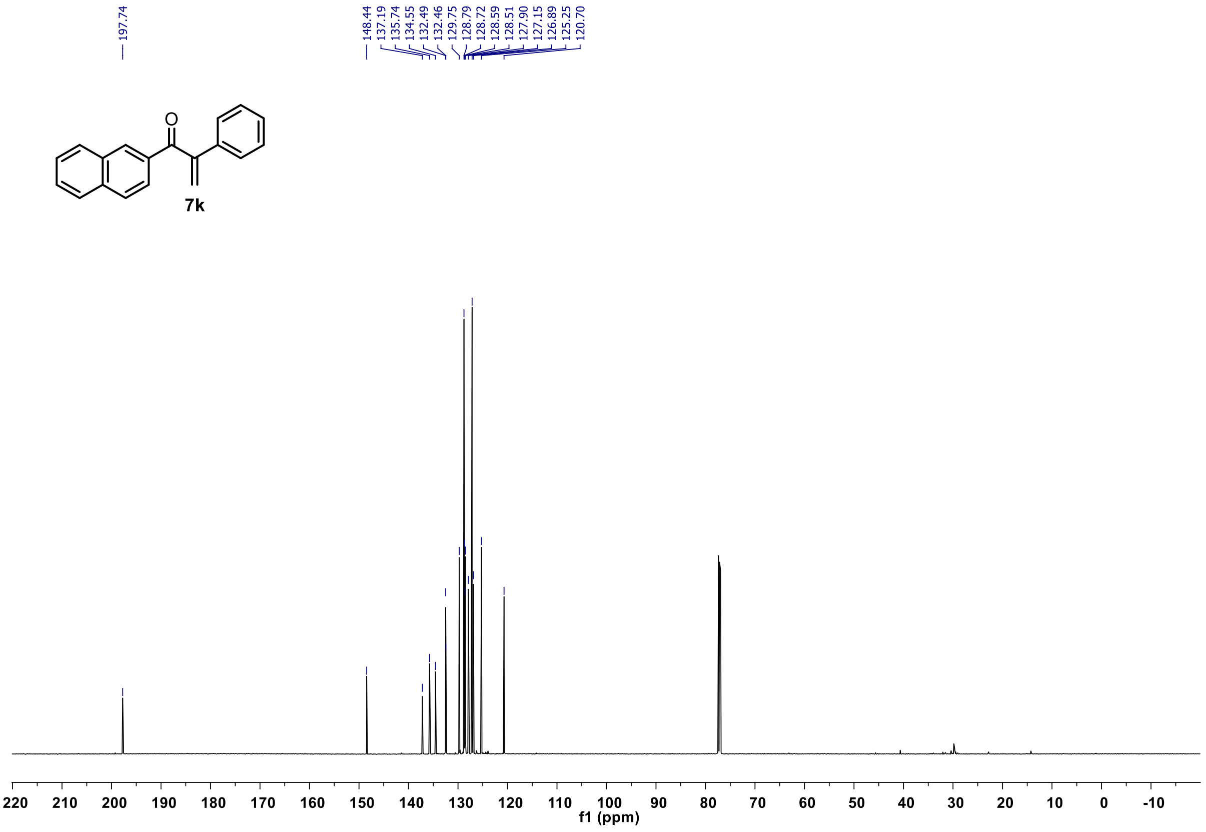

^{13}C NMR in $CDCl_3$ at 151 MHz

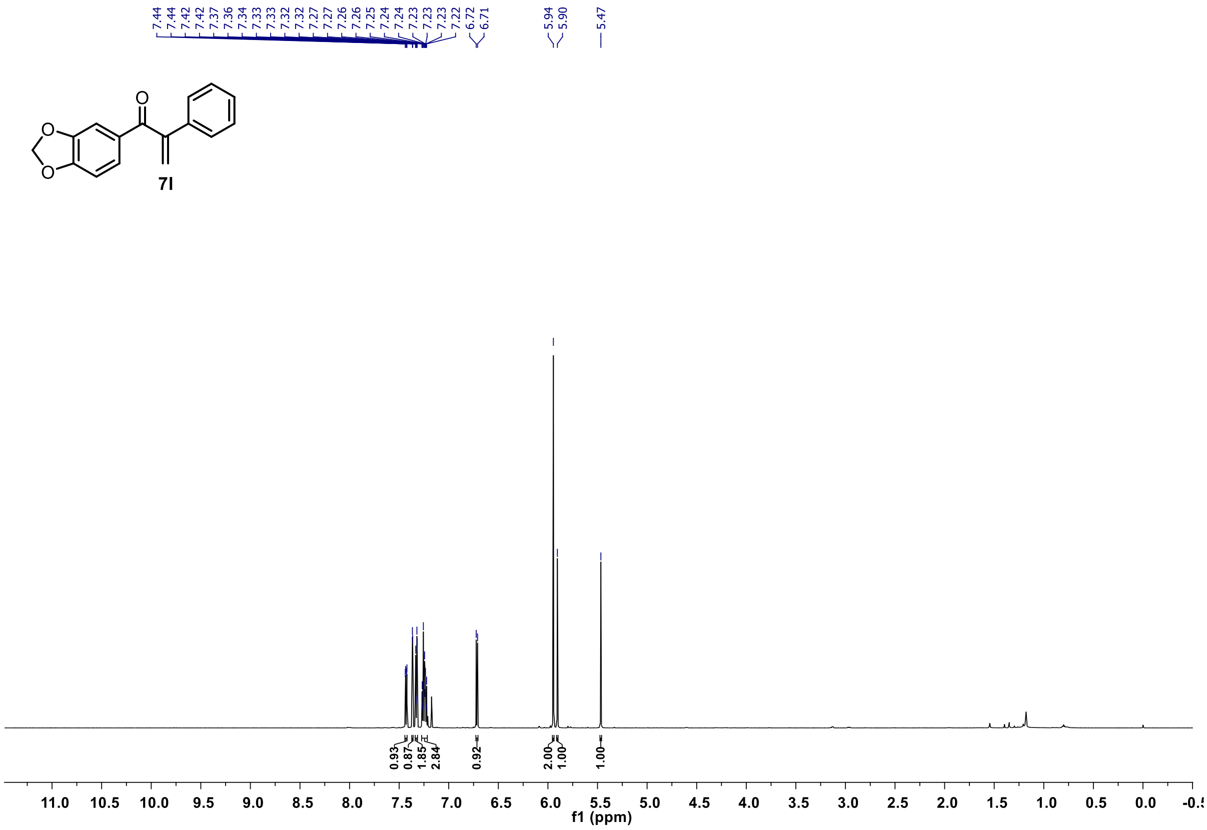

^{1}H NMR in $CDCl_3$ at 600 MHz

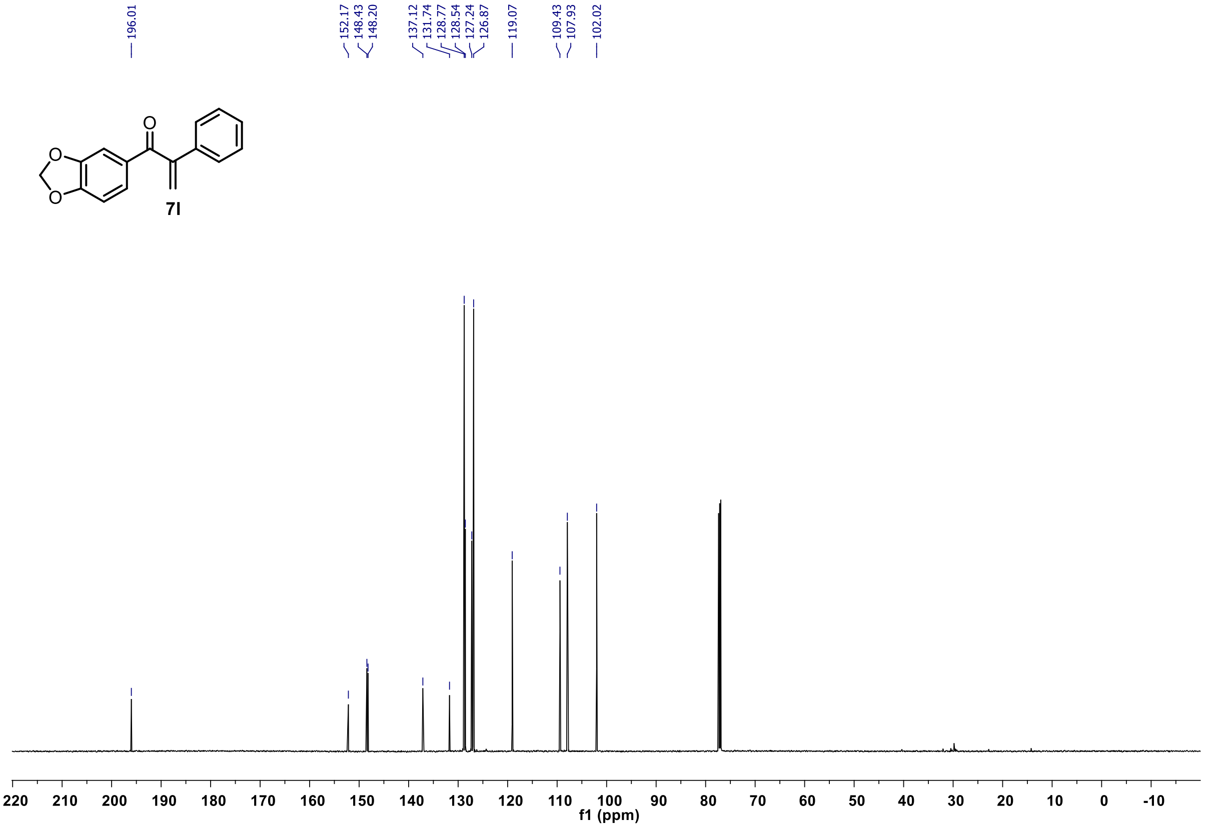

^{13}C NMR in $CDCl_3$ at 151 MHz

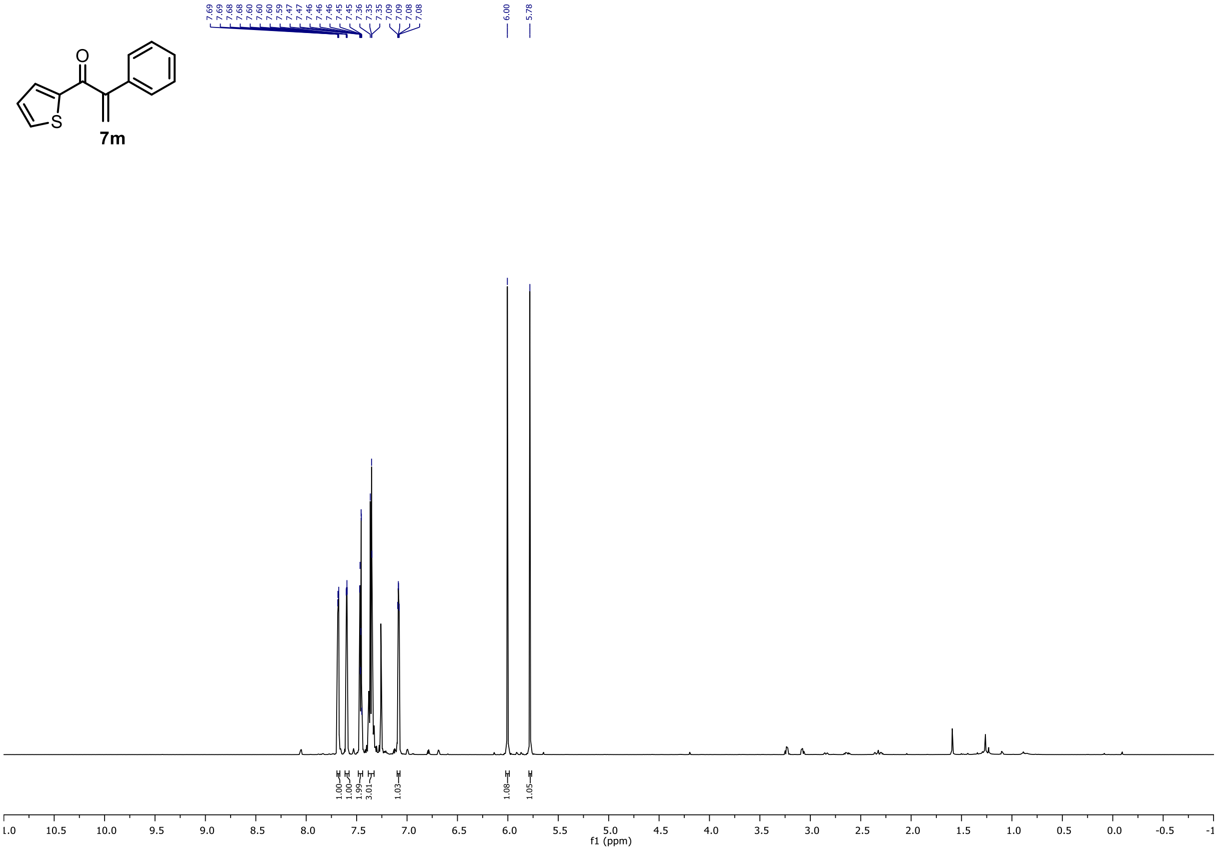

^{1}H NMR in $CDCl_3$ at 600 MHz

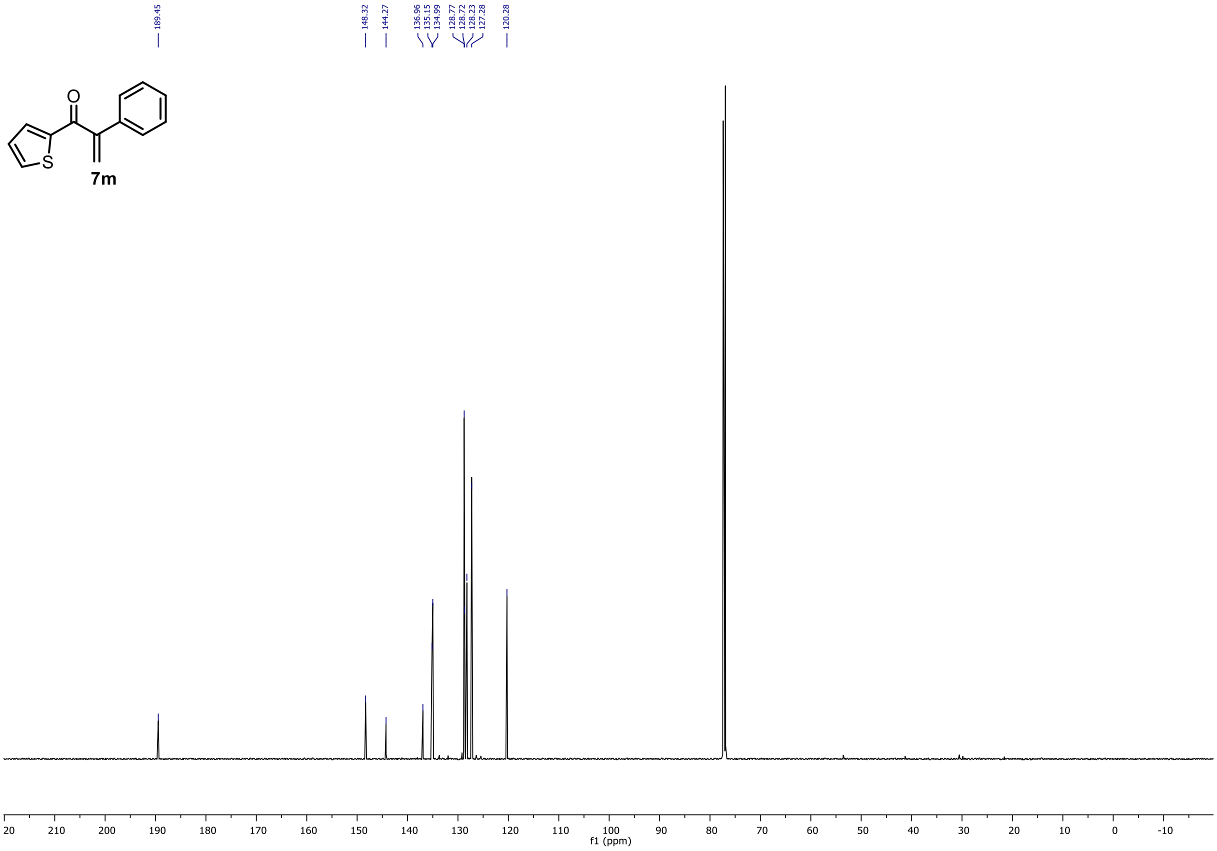

^{13}C NMR in $CDCl_3$ at 151 MHz

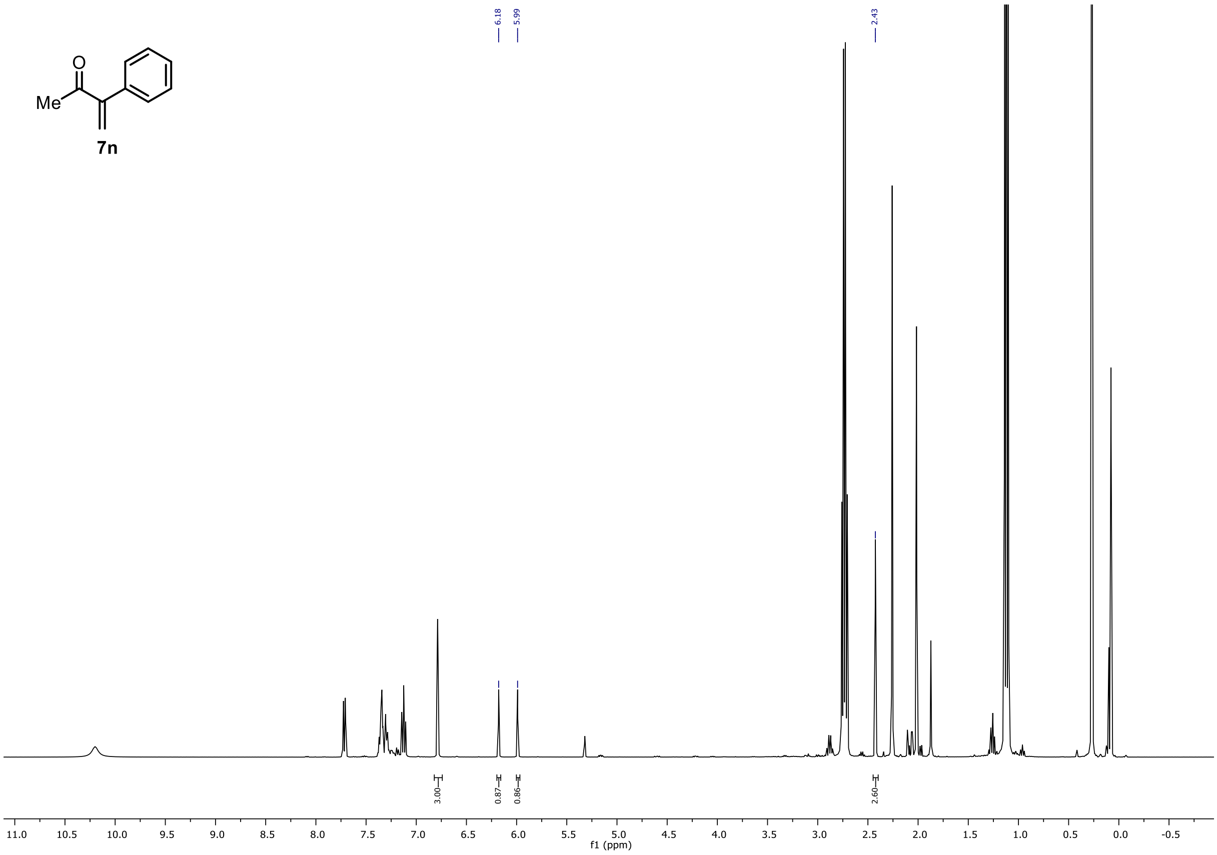

^{1}H q-NMR in $CDCl_3$ at 400 MHz

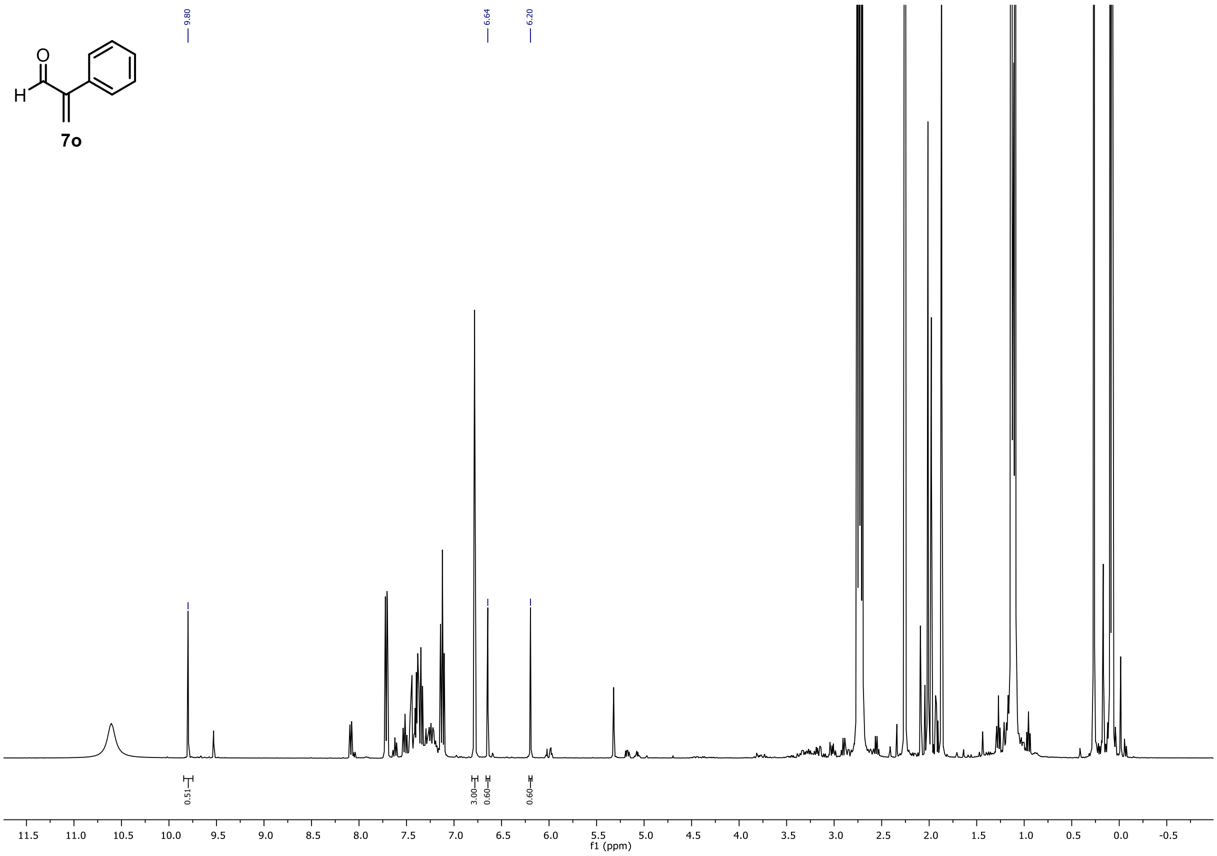

^{1}H q-NMR in $CDCl_3$ at 400 MHz

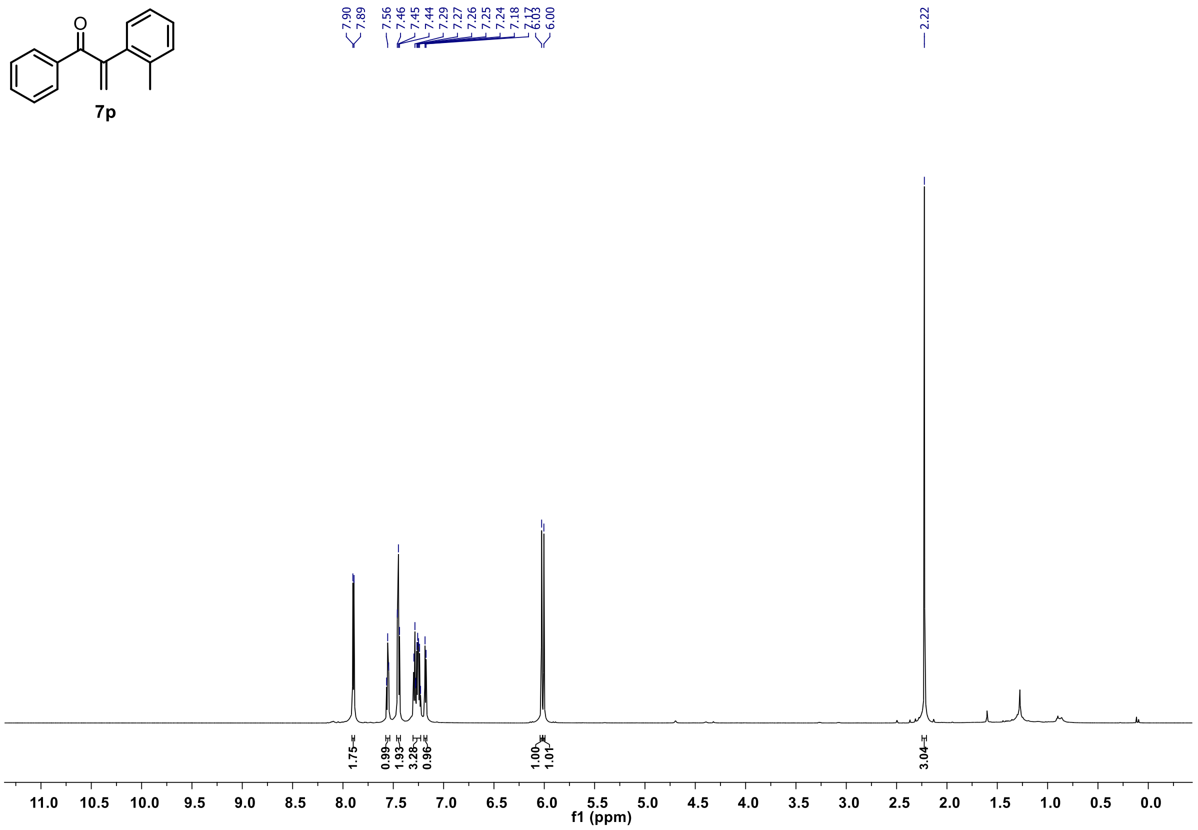

^{1}H NMR in $CDCl_3$ at 700 MHz

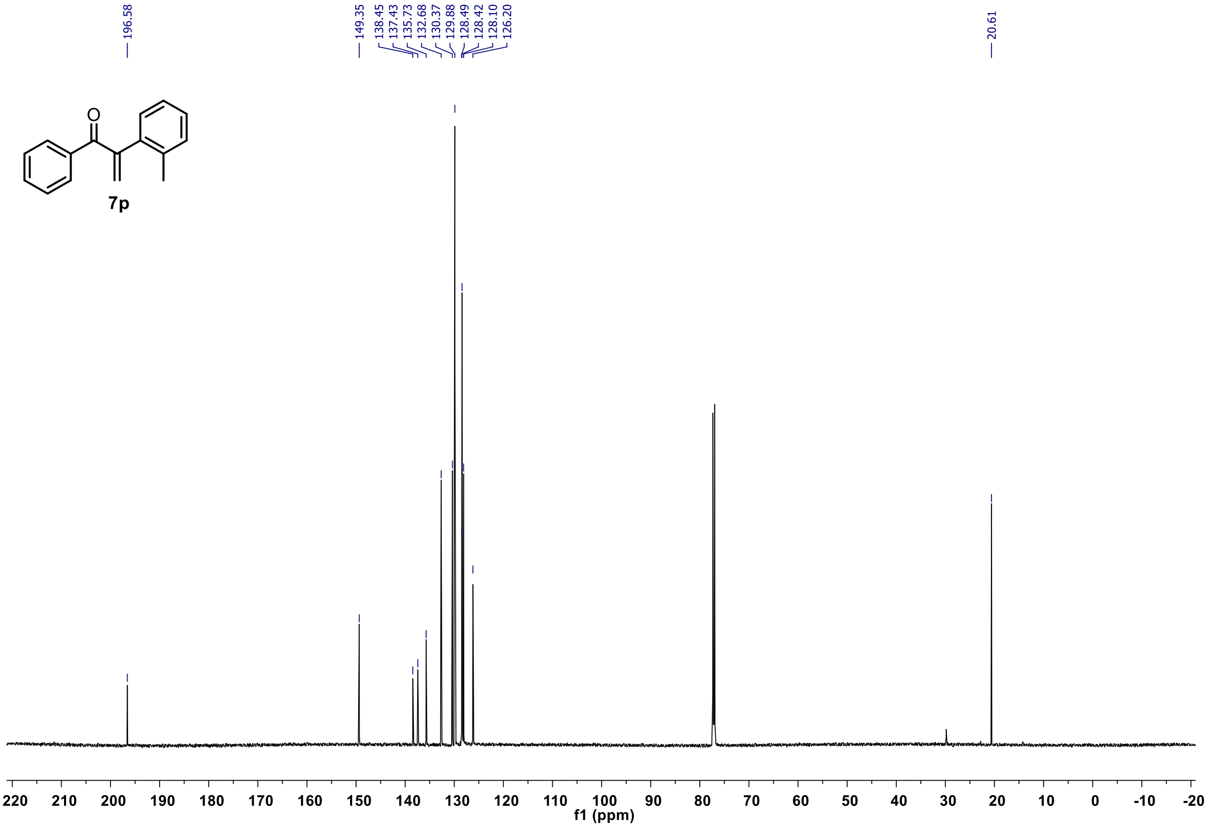

^{13}C NMR in $CDCl_3$ at 176 MHz

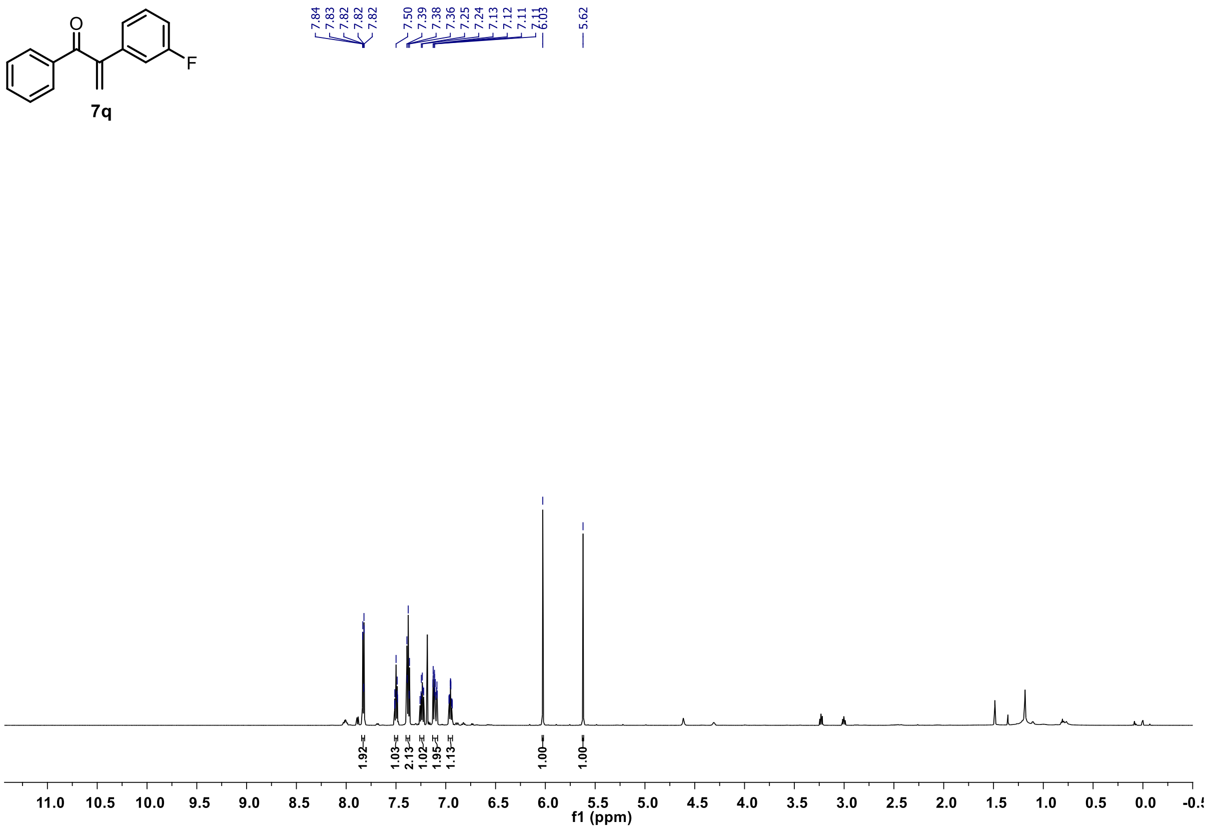

^{1}H NMR in $CDCl_3$ at 600 MHz

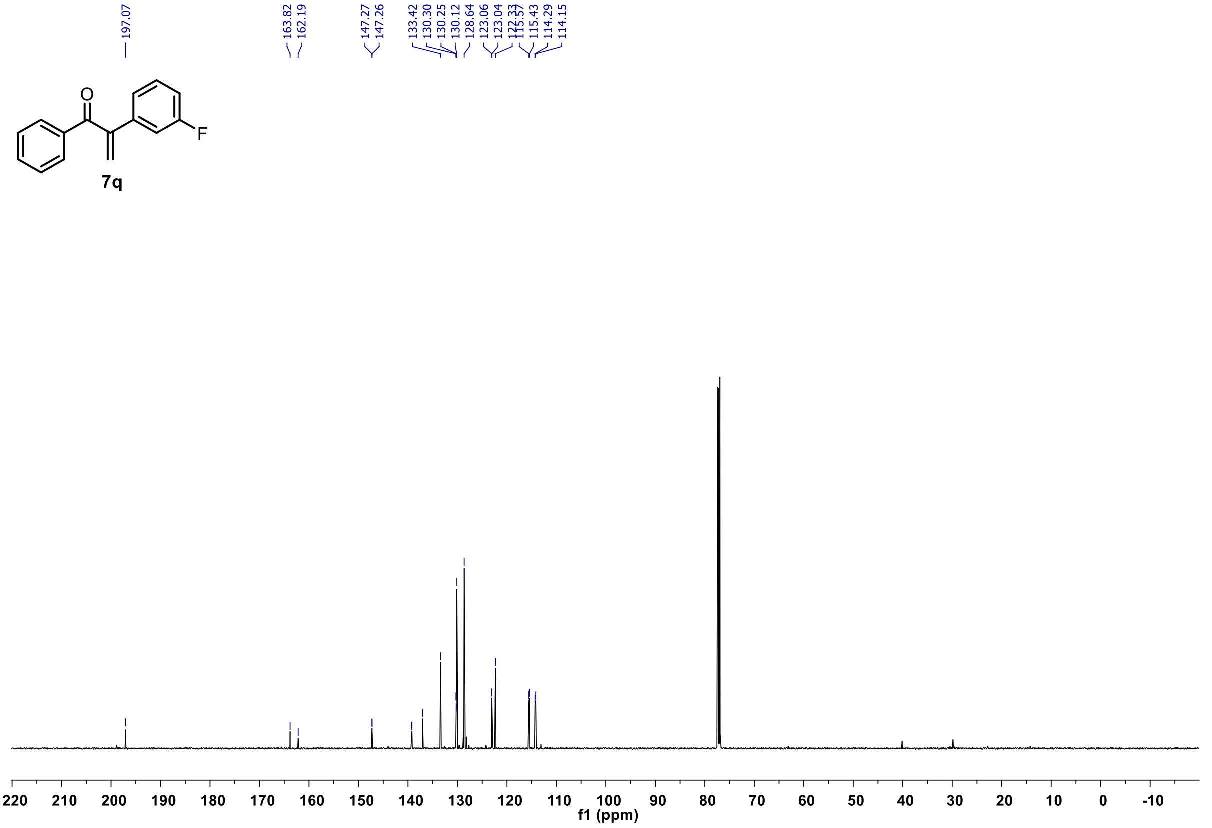

^{13}C NMR in $CDCl_3$ at 151 MHz

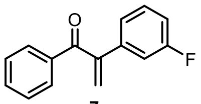

7q

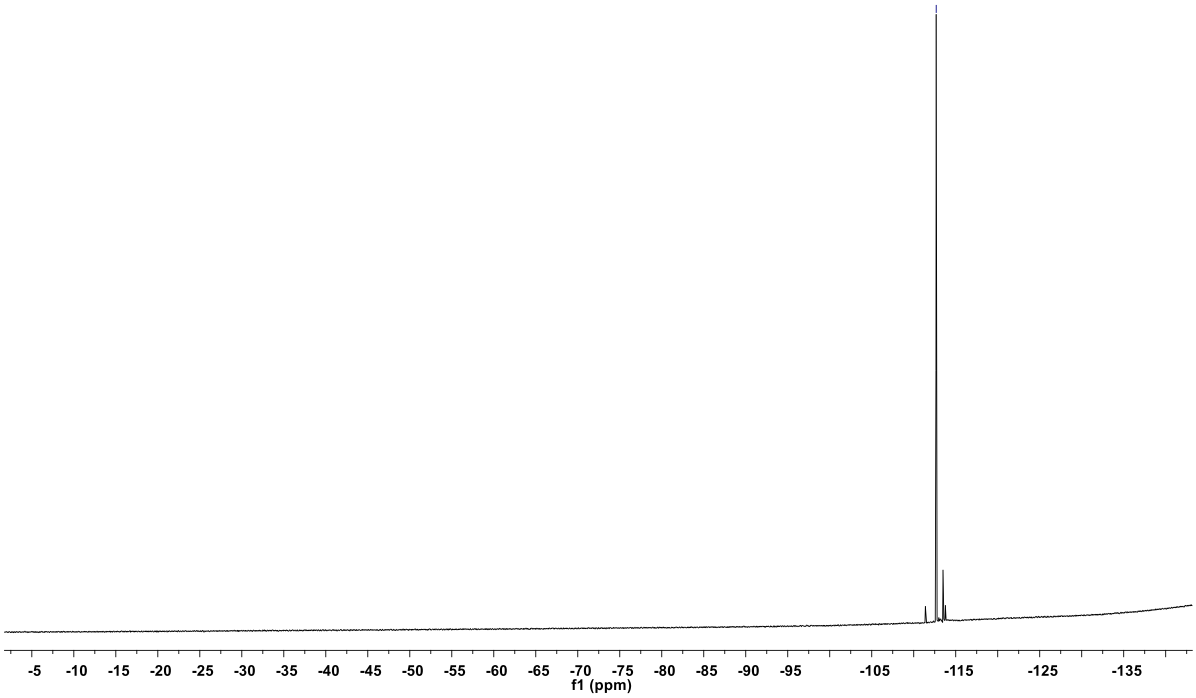

^{19}F NMR in $CDCl_3$ at 565 MHz

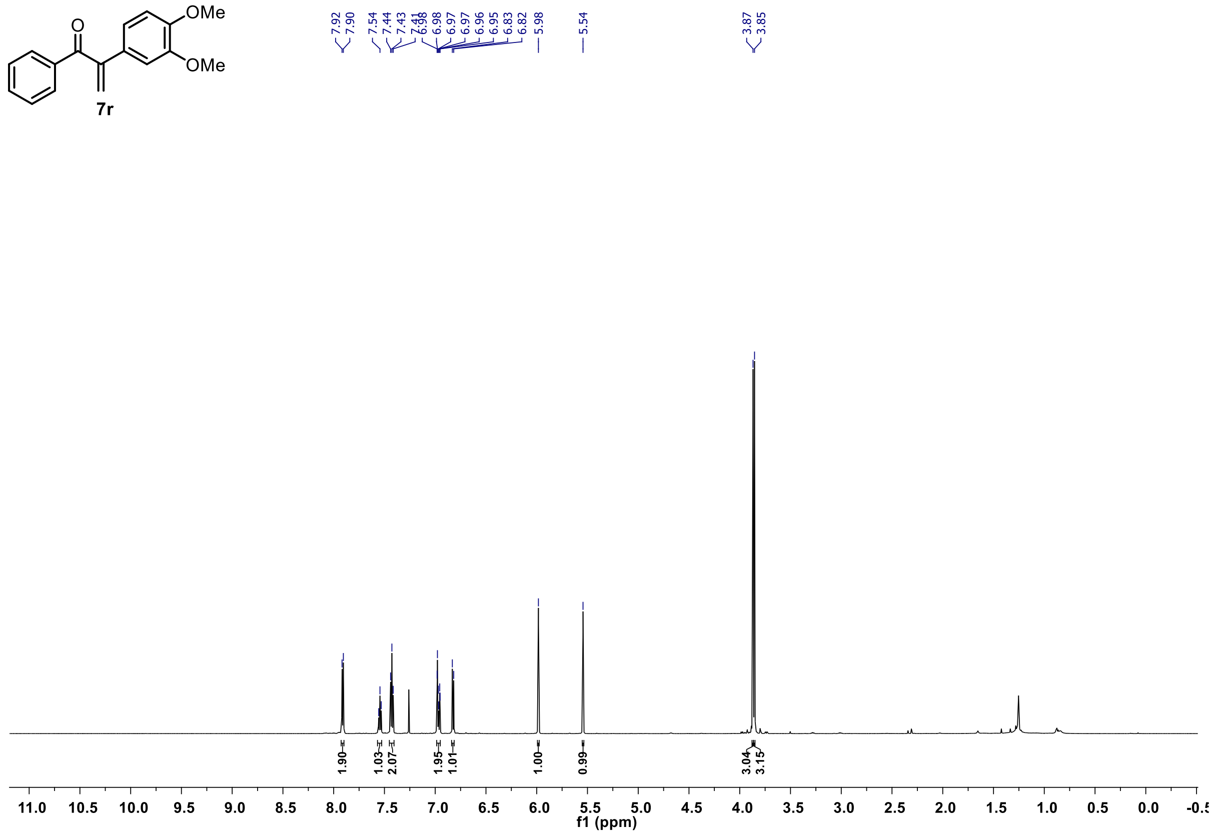

^{1}H NMR in $CDCl_3$ at 600 MHz

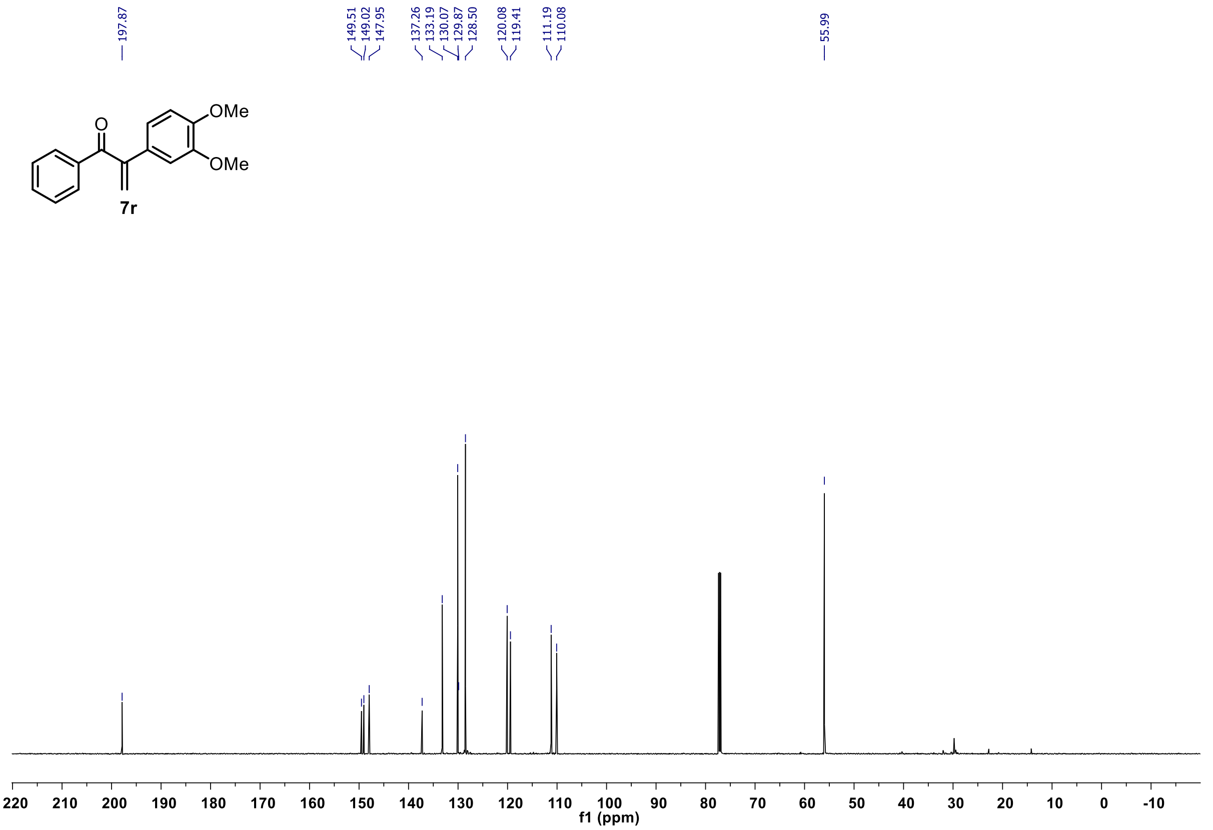

^{13}C NMR in $CDCl_3$ at 151 MHz

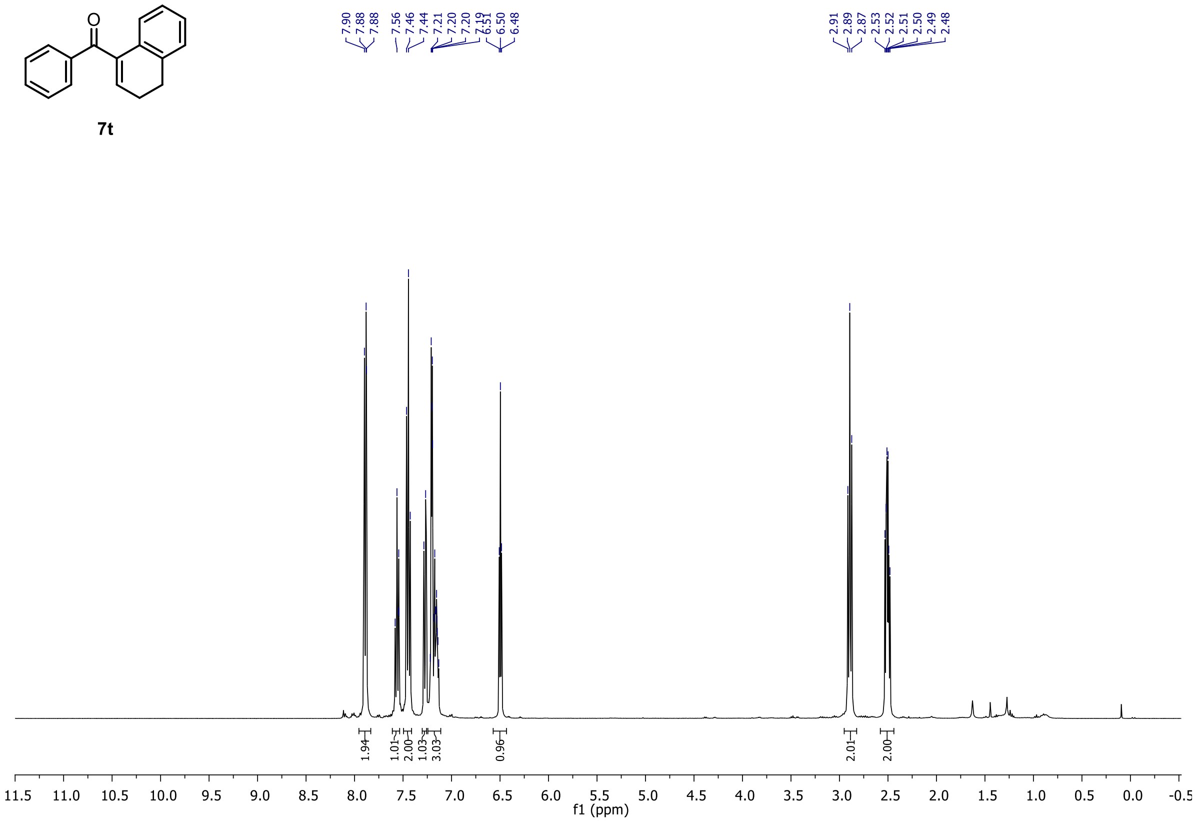

^{1}H NMR in $CDCl_3$ at 400 MHz

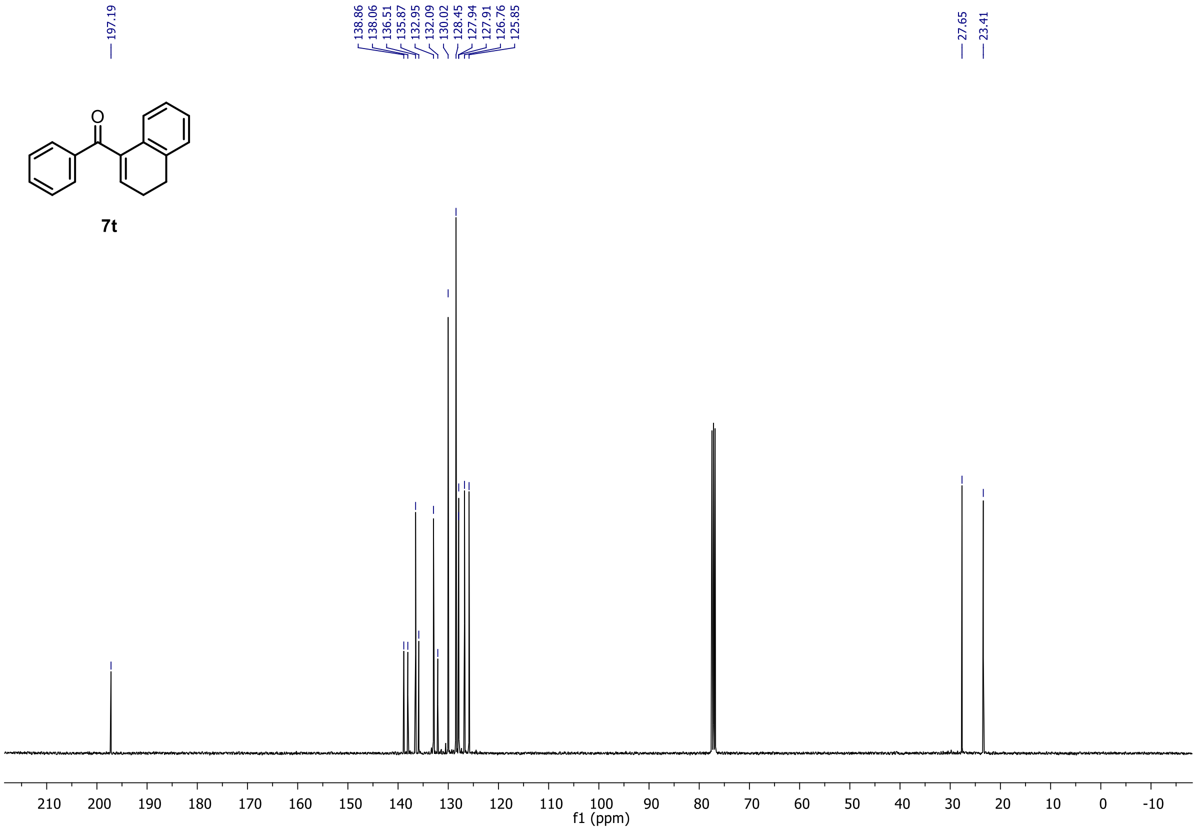

^{13}C NMR in $CDCl_3$ at 101 MHz

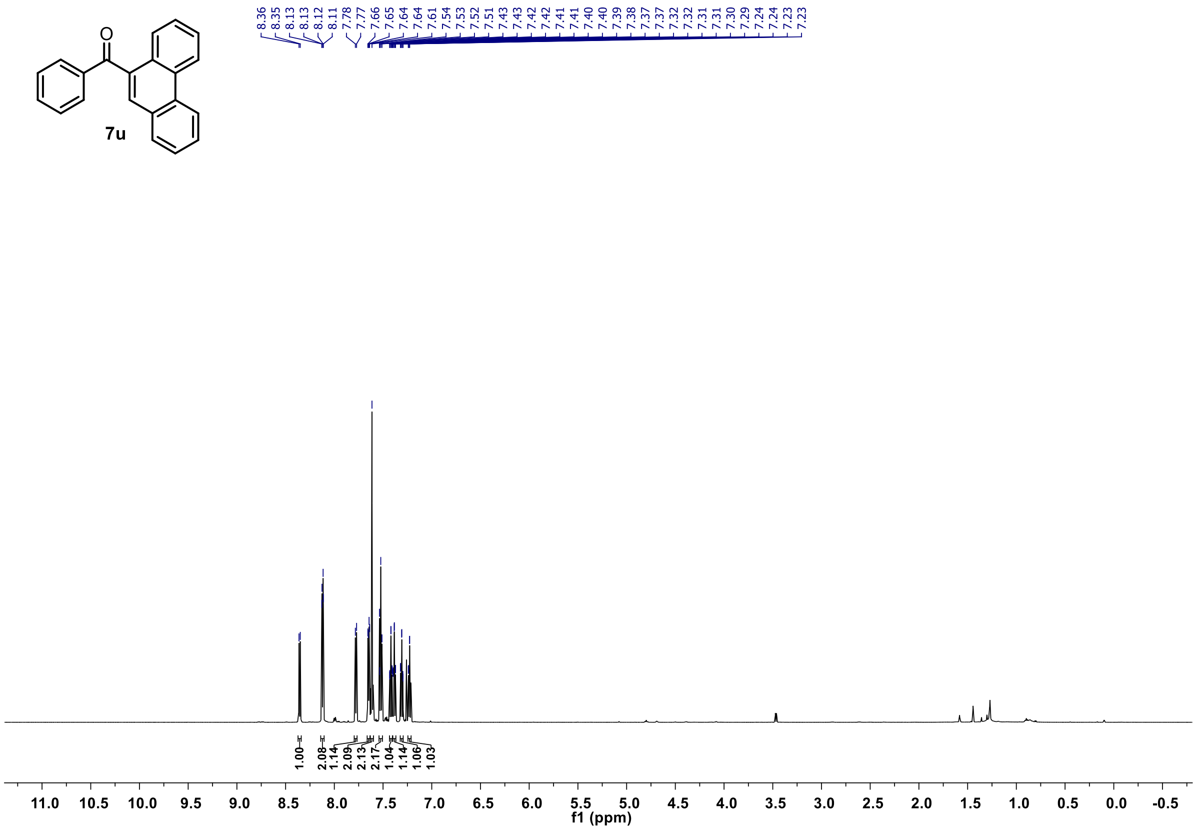

^{1}H NMR in $CDCl_3$ at 600 MHz

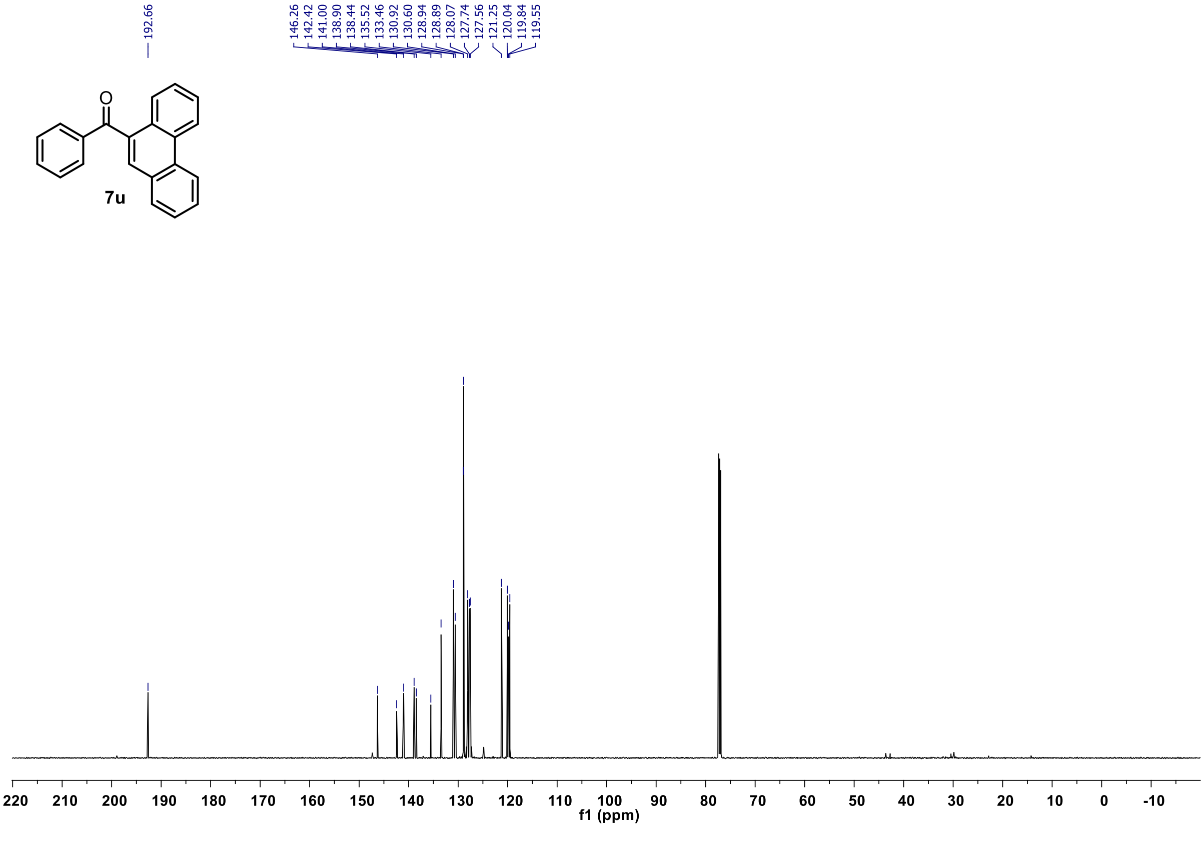

^{13}C NMR in $CDCl_3$ at 151 MHz

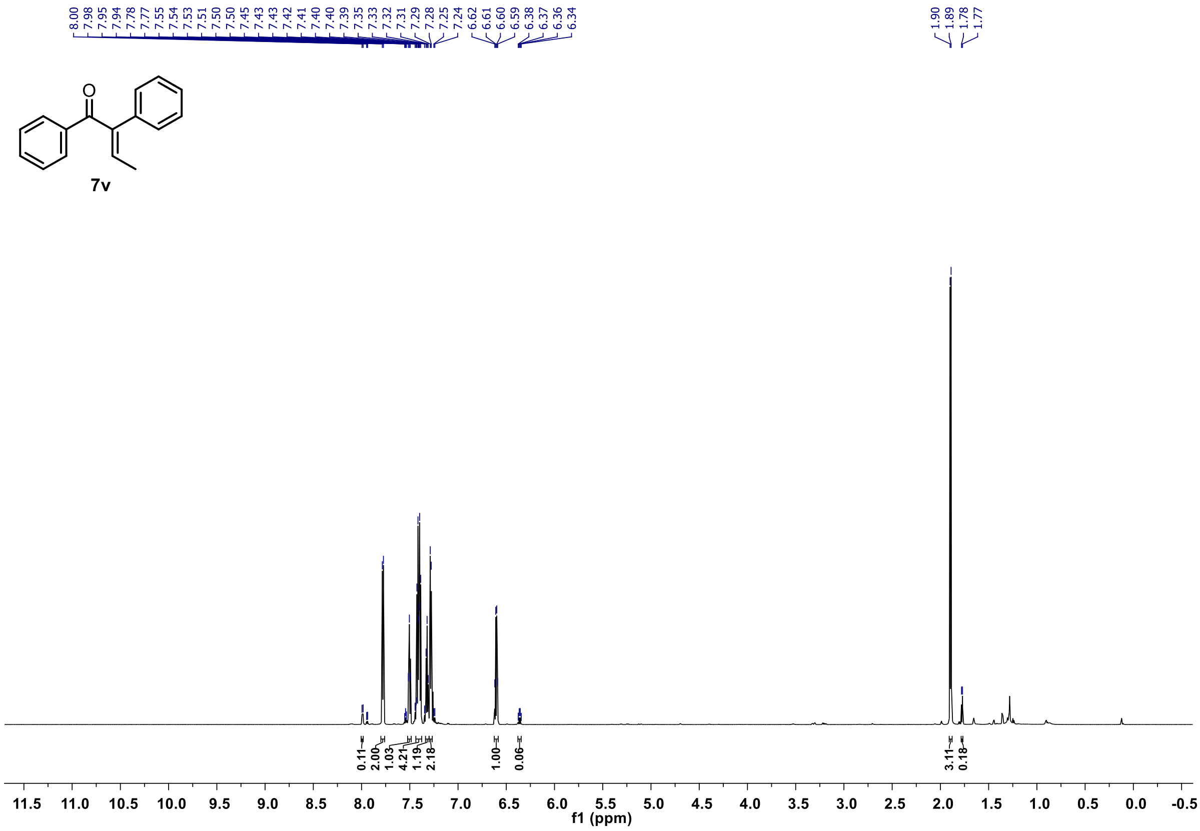

^{1}H NMR in $CDCl_3$ at 700 MHz

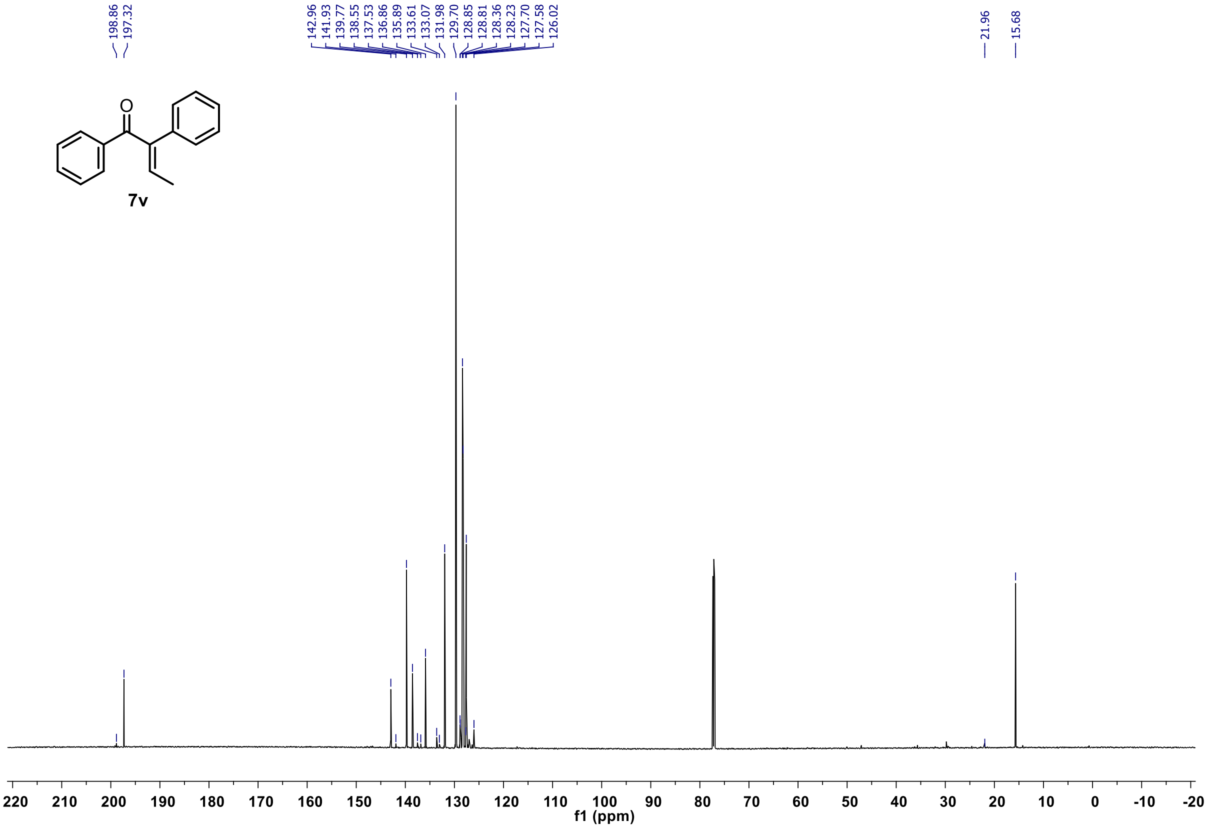

^{13}C NMR in $CDCl_3$ at 176 MHz

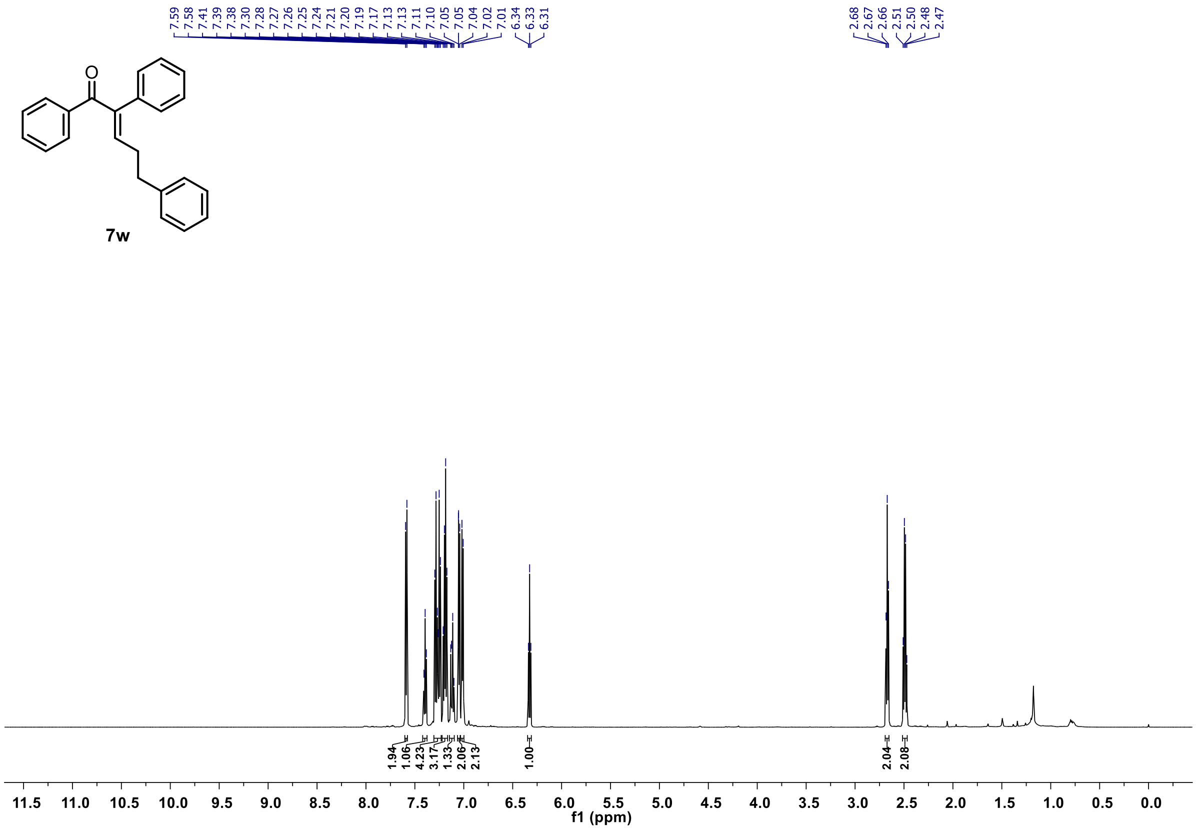

^1H NMR in $CDCl_3$ at 600 MHz

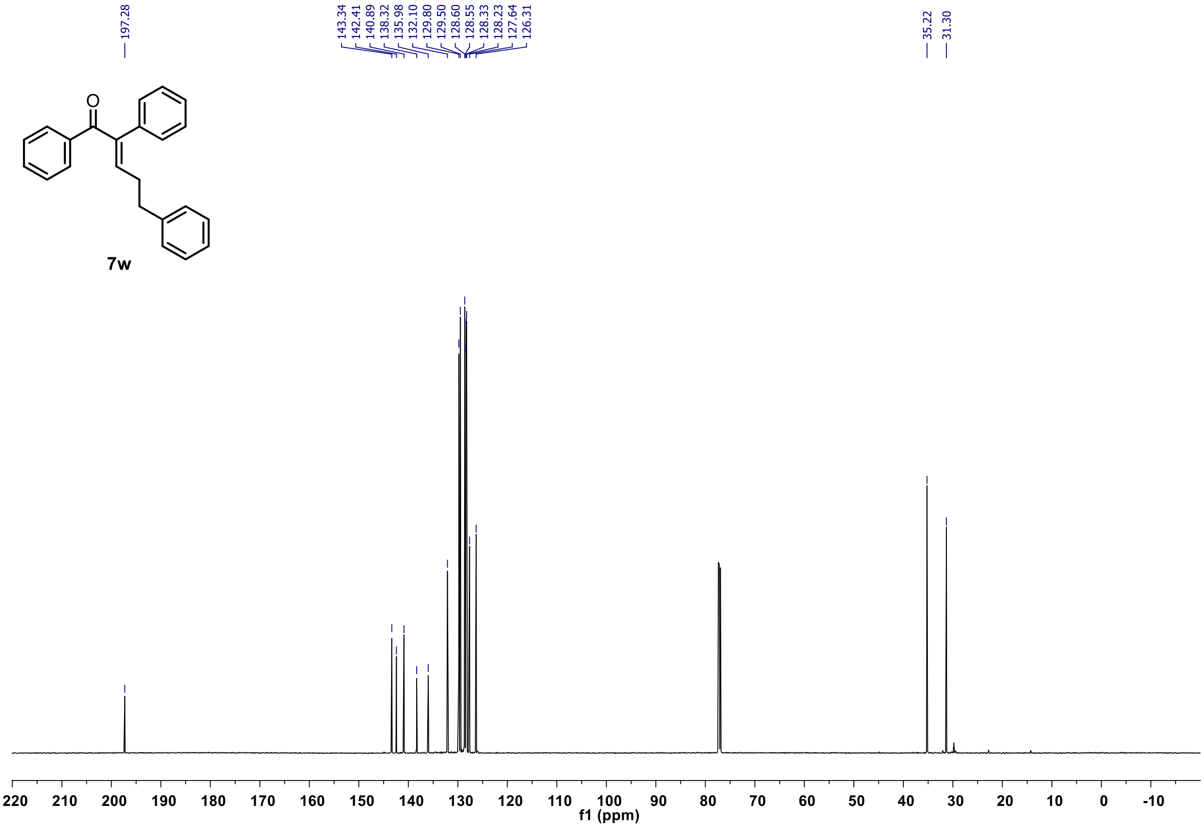

^{13}C NMR in $CDCl_3$ at 151 MHz

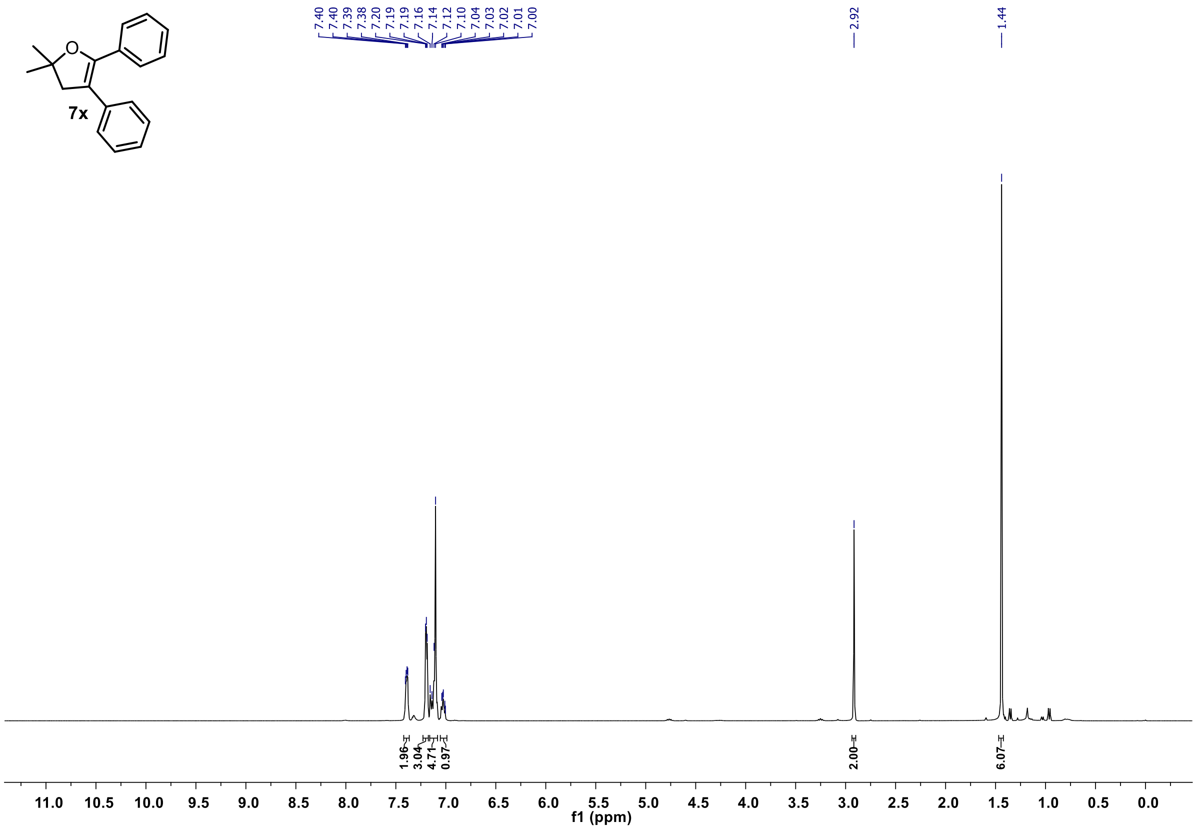

^{1}H NMR in $CDCl_3$ at 400 MHz

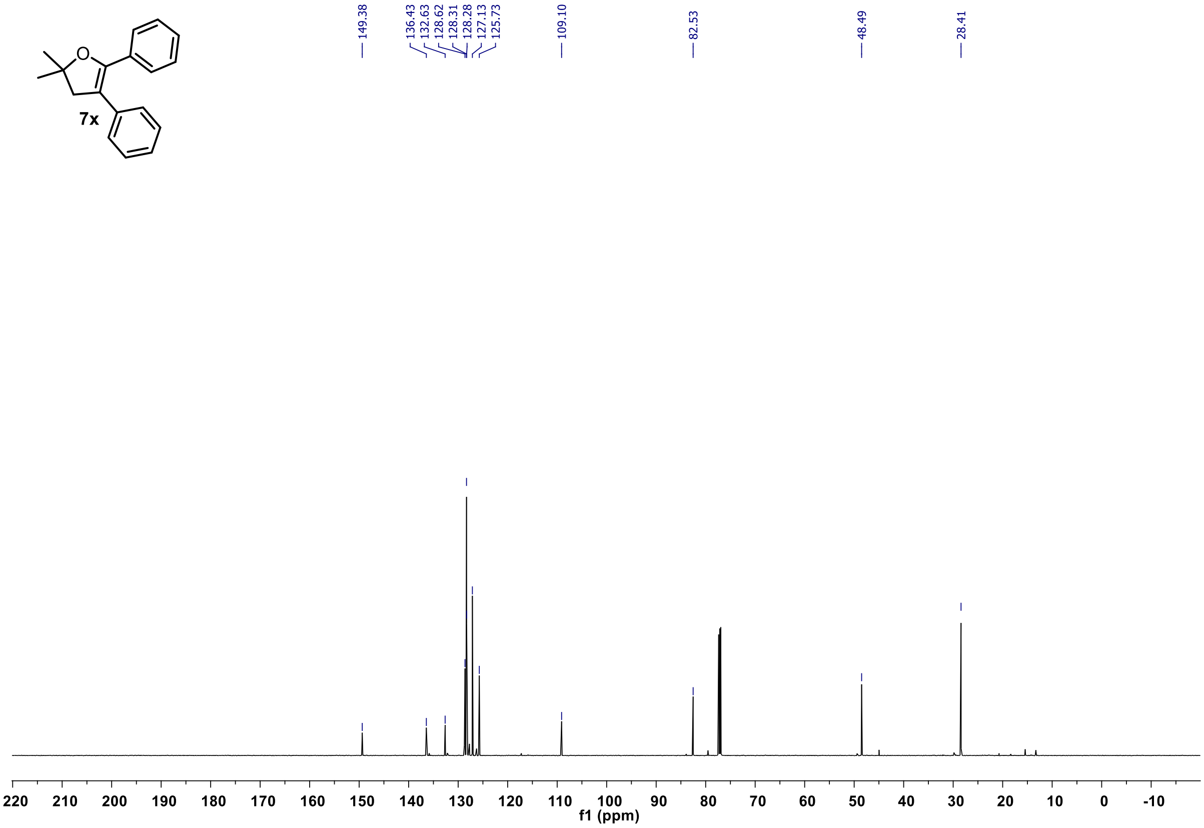

^{13}C NMR in $CDCl_3$ at 151 MHz

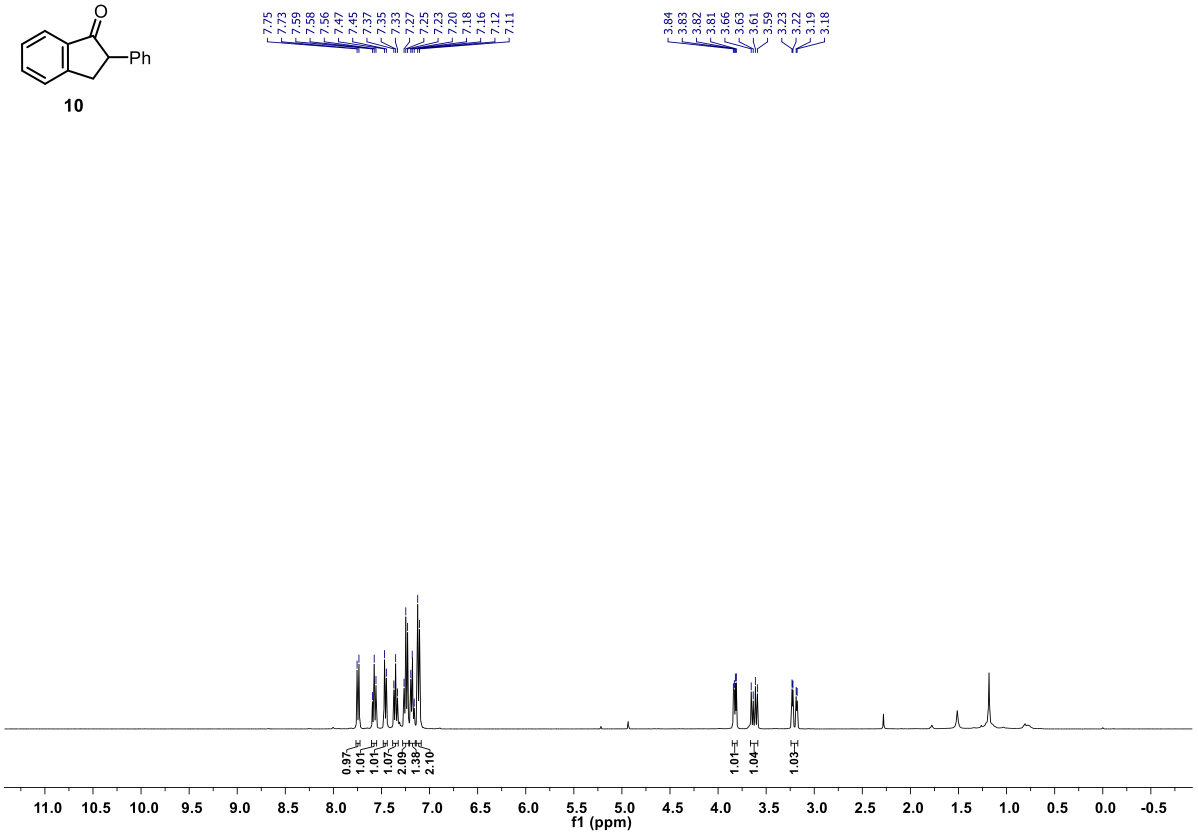

^{1}H NMR in $CDCl_3$ at 400 MHz

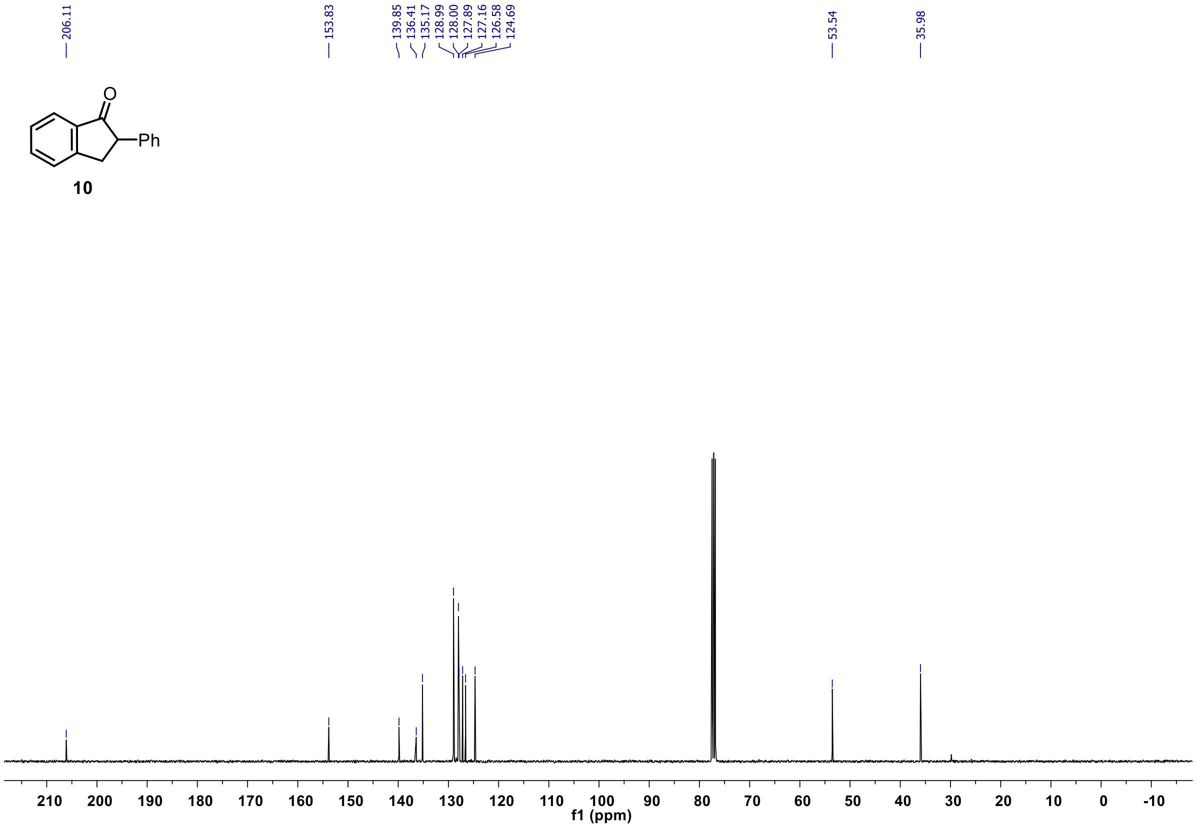

^{13}C NMR in $CDCl_3$ at 101 MHz

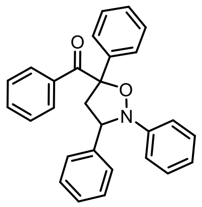

11

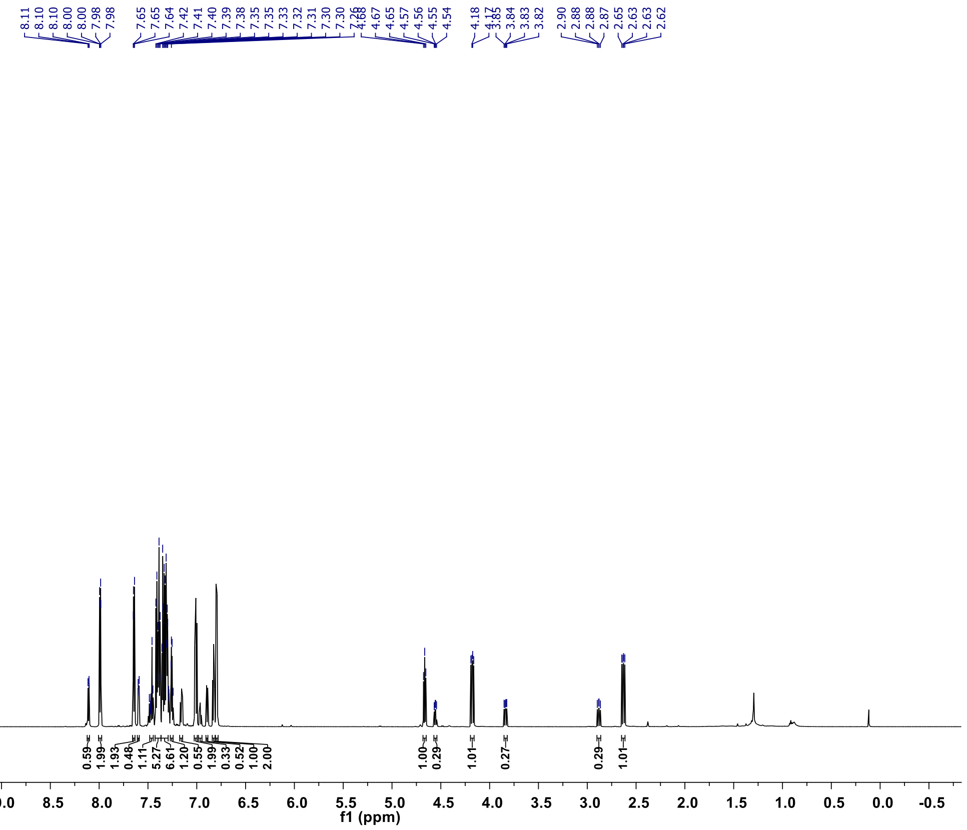

^{1}H NMR in $CDCl_3$ at 700 MHz

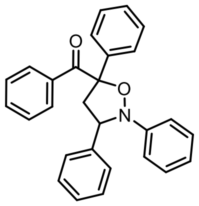

11

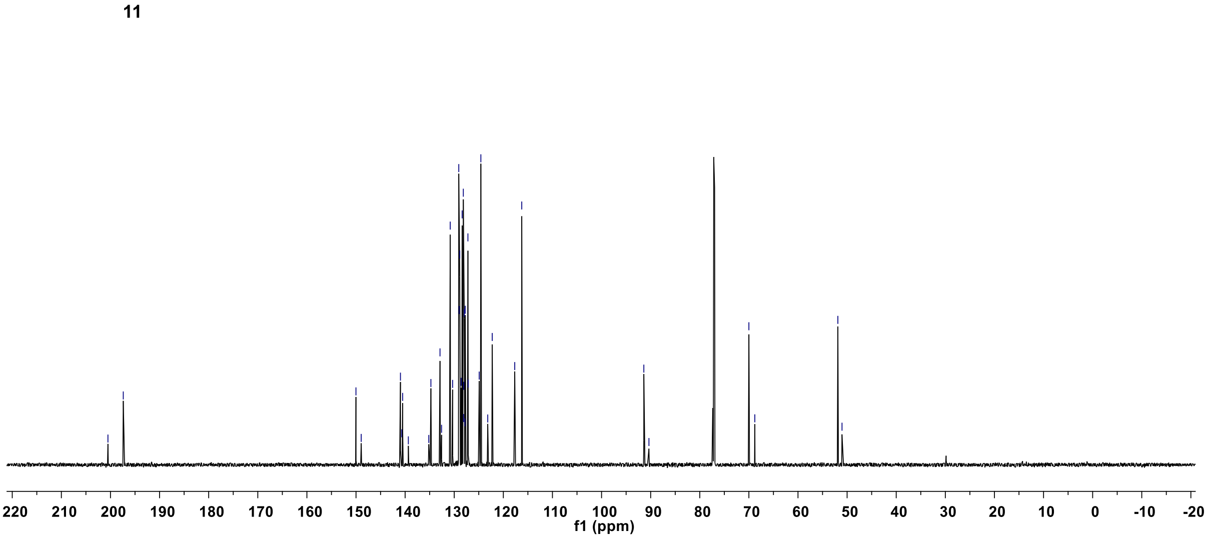

^{13}C NMR in $CDCl_3$ at 176 MHz

5. References

[1] Kubota, K.; Uesugi, M.; Osaki, S.; Ito, H. *Org. Biomol. Chem*. **2019**, *17*, 5680–5683.

[2] Reich, H. J.; Holtan, R. C.; Bolm, C. *J. Am. Chem. Soc*. **1990**, *112*, 5609–5617.

[3] Zhan, X.-Y.; Zhang, H.; Dong, Y.; Yang, J.; He, S.; Shi, Z.-C.; Tang, L.; Wang, J.-Y. *J. Org. Chem*. **2020**, *85*, 6578–6592.

[4] Li, J.; Bauer, A.; Di Mauro, G.; Maulide, N. *Angew. Chem. Int. Ed.* **2019**, *58,* 9816–9819.

[5] Liao, Y.-X.; Hu, Q.-S. *J. Org. Chem*. **2010**, *75*, 6986–6989.

[6] Wang, R.; Ma, J.; Li, F. *J. Org. Chem*. **2015**, *80*, 10769–10776.

[7] Pandey, B.; Xu, S; Ding, K. *Org. Lett.* **2019**, *21*, 7420–7423.

[8] Cho, C. S.; Shim, S. C. *J. Organomet. Chem.* **2006**, *691*, 4329–4332.

[9] Tsuda, T.; Satomi, H.; Hayashi, T.; Saegusa, T. *J. Org. Chem*. **1987**, *52*, 439–443.

[10] Okoromoba, O. E.; Jang, E. S.; McMullin, C. L.; Cundari, T. R.; Warren, T. H. Okoromoba, Otome; Jang, Eun Sil; McMullin, Claire; Cundari, Thomas; Warren, Timothy H. *ChemRxiv. Preprint.* **2019**, doi: org/10.26434/chemrxiv.11407116.v1.

[11] Lautens, M.; Hiebert, S. *J. Am. Chem. Soc*. **2004**, *126*, 1437–1447.

[12] Peng, C.; Wang, Y.; Wang, J. *J. Am. Chem. Soc.* **2008**, *130*, 1566–1567.

[13] Mitsudo, T.; Kadokura, M.; Watanabe, Y. *J. Org. Chem.* **1987**, *52*, 3186–3192.

[14] Kim, T.; Kim, K. *Tetrahedron Lett*. **2002**, *43*, 3021–3024.

[15] Hickman, D. N.; Hodgetts, K. J.; Mackman, P. S.; Wallace, T. W.; Wardleworth, J. M. *Tetrahedron*, **1996**, *52*, 2235–2260.

[16] Lee, B.; Chirik, P. J. *J. Am. Chem. Soc.* **2020**, *142*, 2429–2437.

[17] Cao, J.; Miao, M.; Chen, W.; Wu, L.; Huang, X. *J. Org. Chem*. **2011**, *76*, 9329–9337.

[18] Filho, E. P. S.; Rodrigues, J. A. R.; Moran, P. J. S. *Tetrahedron: Asymmetry* **2001**, *12*, 847–852.

[19] Lee, D.; Ruy, T.; Park, Y.; Lee, P. H. *Org. Lett*. **2014**, *16*, 1144–1147.

[20] Hu, W.; Sun, S.; Cheng, J. *J. Org. Chem*. **2016**, *81*, 4399–4405.

[21] Chen, P.-H.; Sieber, J.; Senanayake, C. H.; Dong, G. *Chem. Sci*. **2015**, *6*, 5440–5445.

[22] Gunawardene, P. N.; Luo, W.; Polgar, A. M.; Corrigan, J. F.; Workentin, M. S. *Org. Lett.* **2019**, *21*, 14, 5547–5551.
